# A resource-efficient structure-based workflow for fragment progression enables parallel hit discovery and validation of functionally diverse modulators of NCS-1 protein–protein interactions

**DOI:** 10.1039/d6sc02031c

**Published:** 2026-09-02

**Authors:** Daniel Muñoz-Reyes, Kate K. Fieseler, Max Winokan, Mathew Golding, Eda Capkin, Matteo Ferla, Sara Pérez-Suárez, Charlie W. E. Tomlinson, Peter G. Marples, Celia Miró-Rodríguez, Lorena Aguado, Alicia Mansilla, Daren Fearon, Warren Thompson, Frank von Delft, María José Sánchez-Barrena

**Affiliations:** a Department of Crystallography and Structural Biology, Institute of Physical-Chemistry “Blas Cabrera”, CSIC Serrano 119 Madrid 28006 Spain xmjose@iqf.csic.es; b Department of Statistics, University of Oxford Oxford OX1 3LB UK; c Research Complex at Harwell, Harwell Science and Innovation Campus Didcot OX11 0FA UK frank.von-delft@diamond.ac.uk; d Centre for Medicines Discovery, University of Oxford Oxford OX3 7DQ UK; e Diamond Light Source Ltd, Harwell Science and Innovation Campus Didcot OX11 0QX UK; f Department of Neurobiology, Instituto Ramón y Cajal de Investigación Sanitaria, Hospital Universitario Ramón y Cajal Madrid Spain; g Department of Systems Biology, Universidad de Alcalá Madrid Spain; h Department of Biochemistry, University of Johannesburg Johannesburg 2006 South Africa

## Abstract

Efficient drug discovery workflows ideally generate data that directly address key translational milestones, including confirmation of target engagement and binding pose, structure–activity relationships (SARs), and biological relevance, within rapid and resource-efficient experimental cycles. Here, we describe a data-driven, automation-assisted, customizable framework for fragment-to-hit progression that directly delivers structurally validated hit series primed for rapid SAR exploration by exploiting the high-throughput crystallography available at synchrotrons. This recently evolved direct-to-biology approach combines X-ray crystallographic fragment screening with algorithmically-guided fragment merging and reagent prioritization; low-cost robotic array synthesis and reaction production assessment by LC-MS; and finally orthogonal biophysical evaluation of crude reaction mixtures for binding assessment and 3D binding pose using grating-coupled interferometry and crystallography, respectively. We demonstrated the effectiveness of the strategy on a challenging target class by collectively progressing a large set of fragment hits through a single DMTA cycle comprising over 250 synthetically diverse compounds, enabling rapid, resource- and cost-effective exploration of the off-catalogue chemical space. This led to the discovery of protein–protein interaction modulators of Neuronal Calcium Sensor 1 (NCS-1), a key regulator in the central nervous system with therapeutic relevance, which contains a large interaction pocket capable of accommodating multiple protein partners. We advanced fragments into scaffold series that selectively engage biologically validated subpockets and, importantly, revealed allosteric and cryptic binding sites, critical for achieving specificity in target modulation and subsequent hit-to-lead generation. The approach is general, engineerable and scalable and provides proof of principle for how to expand the scope of fragment-based hit discovery.

## Abstract 2

A data-driven, automation-assisted, customizable framework for fragment-to-hit progression that directly delivers structurally validated hit series primed for rapid SAR exploration and exploits high-throughput crystallography at synchrotrons.

## Graphical abstract

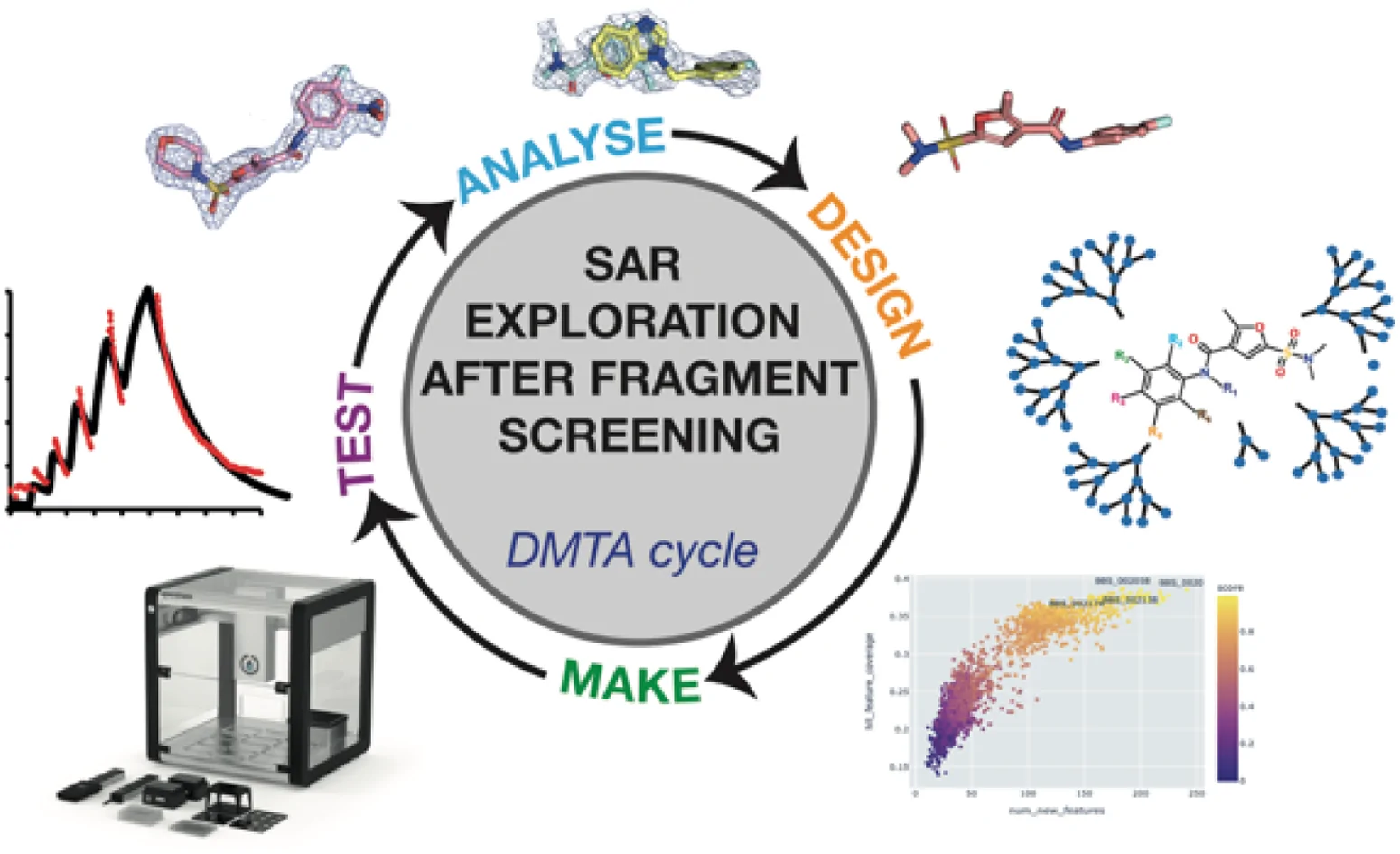

## Introduction

The Neuronal Calcium Sensor 1 (NCS-1) is a calcium signaling protein with multiple functions in the central nervous system and in other tissues.^1^ NCS-1 interacts with several G-protein-coupled receptors (GPCRs) (adenosine A_2A_, cannabinoid CB_1_ and dopamine D_2_ receptors) and proteins implicated in regulating G-signaling, among others, the molecular chaperone and guanine exchange factor (GEF) Ric-8A^2^ or the kinase GRK2.^3^ In addition, it also interacts with calcium channels such as the InsP_3_ receptor, which regulates cytosolic calcium concentrations.^4^ The physiological consequences of some of these protein–protein interactions have been studied. NCS-1 modulates the activity of Ric-8A, and the protein complex is implicated in synaptogenesis and neurotransmitter release.^2,5^ NCS-1 modulates the trafficking of the dopamine D_2_ receptor (hereinafter D_2_R) to the plasma membrane.^6^ In addition, NCS-1 directly interacts with the cannabinoid CB_1_ receptor (hereinafter CB_1_R)^7^ through a helical C-terminal region rich in phosphorylatable residues that is implicated in the modulation of the activity of the GPCR.^8^

NCS-1 interacts with its binding partners through a large solvent-exposed hydrophobic crevice, a structural feature shared across the Neuronal Calcium Sensor (NCS) protein family. The 16 protein members display up to 60% sequence identity, adopt similar overall topologies, and conserve key hydrophobic residues within the binding pocket that are critical for partner recognition. Structural analyses of several NCS-protein complexes indicate that binding specificity is dependent on the shape and dimensions of the cavity. The cavity is influenced by (i) the Ca^2+^ content of the protein, (ii) the presence of hydrophilic residues at the border of the crevice, which serve as interaction hotspots, and (iii) the C-terminal and dynamic helix H10, which can insert into the cavity. Helix H10 contains an unstructured C-terminal tail that allows this region to adopt multiple conformations, thereby contributing to the shape of the crevice.^5,9,10^

NCS-1 has been implicated in disease pathogenesis.^11^ In the past few years, small-molecule modulators of the interaction between NCS-1 and Ric-8A or the dopamine D_2_ receptor have been reported, supporting the druggability of the NCS-1 protein–protein interaction (PPI) interface and their therapeutic interest in neurodegeneration, psychiatric and neurodevelopmental disorders.^6,9,10,12,13^ Given the large size of the PPI interface and the multiple binding partners of the calcium sensor, it would be of value to systematically explore the chemical space of this PPI and generate structurally validated chemical scaffolds that engage different regions of the crevice, enabling selective regulation of individual NCS-1 PPIs while avoiding a global effect on the NCS-1 interactome (Fig. 1).

**Fig. 1:**
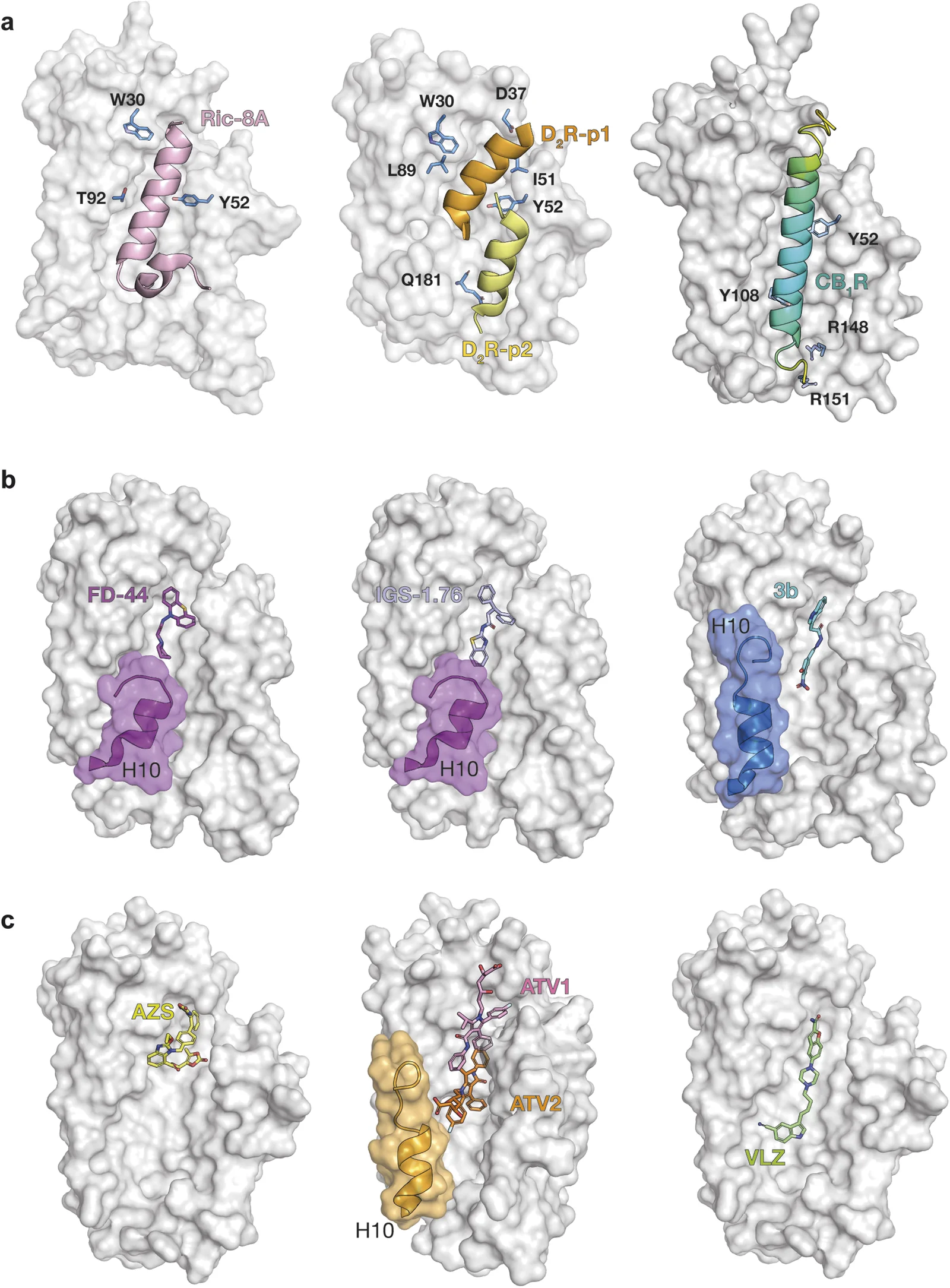
The structure of NCS-1 bound to target proteins and ligand complexes. (a) Ribbon representation of NCS-1 protein structures bound to the molecular chaperone and GEF Ric-8A (PDB: 8AHY^5^), a dopamine D_2_ receptor C-terminal peptide (PDB: 5AER^14^), and an AlphaFold2-Multimer^15,16^ model of the NCS-1/CB_1_R complex. Key NCS-1 residues involved in the interaction with each of the targets are labelled and shown as sticks. (b) and (c) Structures of NCS-1 in complex with PPI regulatory ligands: (b) the NCS-1/Ric-8A PPI inhibitors FD-44 (PDB: 5AAN^9^) and IGS-1.76 (PDB: 6EPA^10^) and the PPI stabilizer 3b (PDB: 6QI4^12^); (c) the NCS-1/D_2_R PPI inhibitors azilsartan medoxomil (AZS, PDB: 9GTO^6^), atorvastatin (ATV, PDB: 9GU6^6^) and vilazodone (VLZ, PDB: 9GU8^6^).

Over the past decade, the development of high-throughput crystallographic fragment screening facilities at synchrotrons has emerged as a powerful methodology for mapping protein binding sites and exploring the chemical space of these targets. This approach enables the identification of novel hits with significant biomedical and therapeutic potential. The XChem facility at Diamond Light Source has enabled screening of over 1000 fragments per week, thanks to advances in crystal identification, automated crystal soaking, crystal mounting, automated data collection and crystallographic data processing.^17,18^ This strategy requires a robust crystal system with highly reproducible crystallization, tolerance to the organic solvent used to dissolve the fragments, and, ideally, diffraction at a resolution better than 2.5 Å.^17^

A persistent bottleneck in turning fragment hits into biologically relevant derivatives relies on the efficient exploitation and exploration of the structural information contained in protein–fragment complexes to guide structure-based drug design. Few existing procedures are both efficient and fully automated.^19–21^ This work builds on a previous XChem fragment-progression exemplar that utilized multi-step robotic array chemistry and direct-to-biology readout, specifically a binding-bite purification of actives (B-SPA) premise with crystallographic readout from leveraging protein–ligand affinity to extract signal from crude reaction mixtures.^22,23^ To make this fragment-progression approach routine and rapid, XChem has been developing algorithmic Design and digitized Make approaches that aim to lower the Design (medicinal/computational chemistry expertise) and Make (synthetic chemistry) barriers, to complete the fragment-progression loop in a single high-value DMTA (Design–Make–Test–Analyze) cycle. The ambition is to generate hit series from a single HT-DMTA cycle that goes “wide”, generating diverse scaffolds from inspiration fragments, and “deep” with SAR around scaffolds.

This XChem fragment-progression workflow was implemented to rapidly (3 months) and cost-efficiently explore the chemical space of the NCS-1 protein–protein interaction interface. Efficient reactant prioritization enabled the low-cost development of fragment-merged derivatives under a limited budget (€11k for reactant purchasing and synthesis and €2.6k for purchasing pure compounds). This approach yielded hundreds of chemically diverse molecules that were evaluated using orthogonal biophysical techniques and crystallography. As a proof of concept, we have demonstrated the PPI modulatory activity of one of the optimized hits and, more importantly, we have established a robust foundation for the design of chemically diverse and selective PPI modulators for this pharmacologically relevant target (Fig. 2). The collective technologies used in this work are collectively called Fast Forward Fragments (FFF) with an emphasis on combining scaffold-design (Fragmenstein),^24^ scaffold-enumeration (Syndirella),^25^ down-selection (HIPPO) (https://github.com/mwinokan/HIPPO) and digitized chemistry (CAR) tools that are adaptable to iterative improvement, engineerable and scalable.

**Fig. 2:**
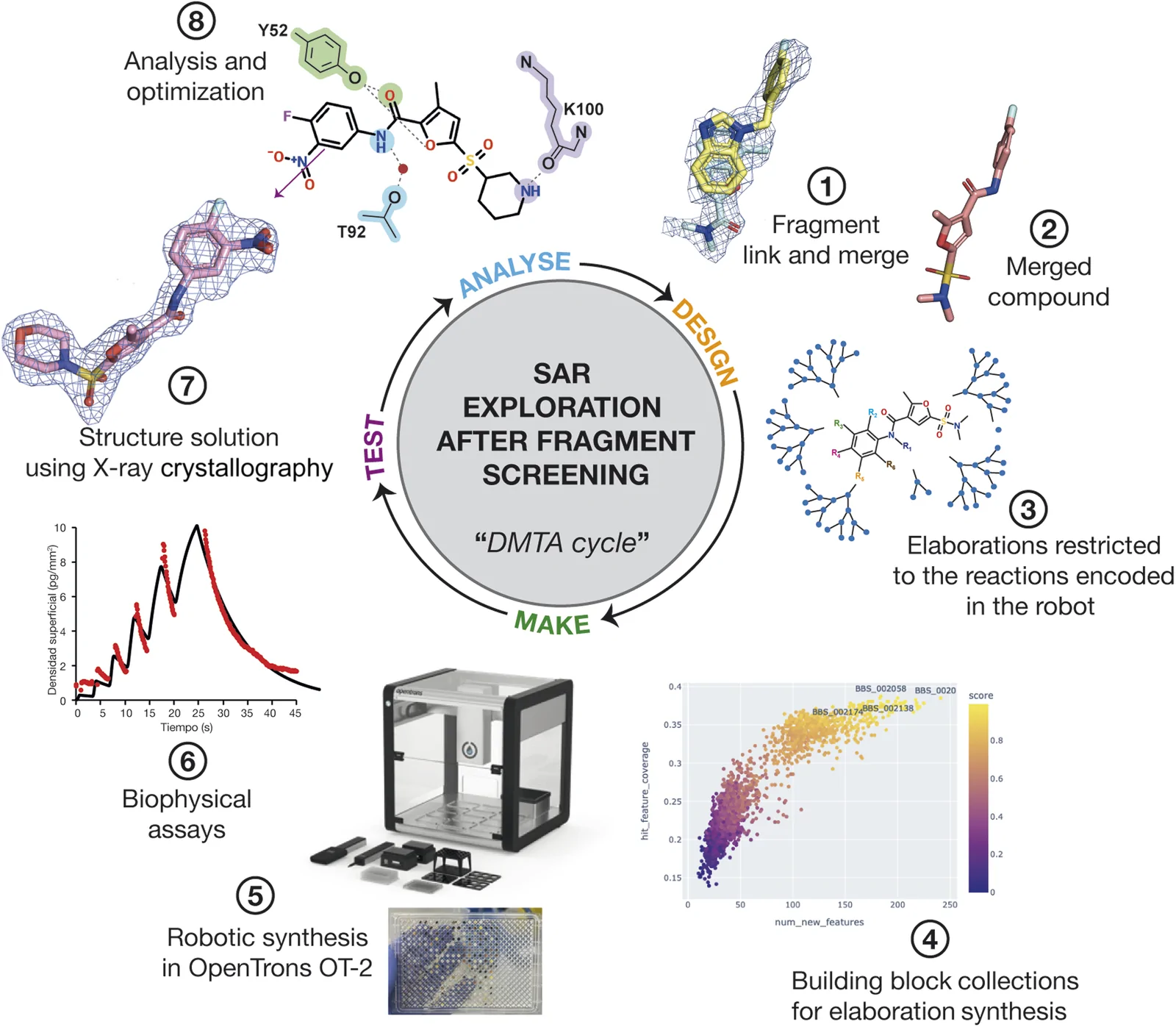
Implementation of the DMTA cycle in NCS-1. The DMTA cycle consists of four distinct phases: design (orange), make (green), test (purple) and analyze (blue). The different steps^1–8^ implemented in this study for the search of new modulators of the interaction between NCS-1 and its target proteins are shown.

## Results

### Crystallographic fragment screening productively samples all subpockets

A previously reported full-length hNCS-1 crystal form^26^ diffracts beyond 2 Å and tolerates DMSO, making it suitable for large-scale fragment screening. Furthermore, its soakability was demonstrated in the structure of NCS-1 bound to the NCS-1/Ric-8A PPI stabilizer 3b (PDB: 6QI4^12^). In the crystal, the NCS-1 hydrophobic crevice is exposed to the solvent, and the C-terminal helix H10 is positioned laterally, allowing compound entry into the cavity where the NCS-1 target proteins bind (Fig. 1c).

Four fragment libraries were selected: DSi-Poised (rapid progression and saturated heterocycles, 876 compounds),^27^ York 3D (substituted aliphatic heterocycles, 106 compounds),^28^ FragLites (halogenated, 31 compounds),^29^ and MiniFrags (Astex pharmacophores, 80 molecules),^30^ comprising a total of 1093 different fragments. The fragment libraries were selected for their chemical diversity and the presence of chemotypes compatible with the hydrophobic crevice of NCS-1. The York 3D library was especially attractive as it contains substituted aliphatic heterocycles, moieties that are contained in previous regulators of the NCS-1/Ric-8A and NCS-1/D_2_R PPI (Fig. 1c and d).^9,10,12^

A total of 1258 crystals were mounted from 1600 crystal drops, and automated data collection was carried out on the I04-1 beamline (SI, Materials and methods). Of the 937 datasets collected, only 540 were of sufficient quality and resolution to solve the corresponding protein–fragment complex structures (Fig. 3). Hit identification was performed with PanDDA.^31^ Initial hit identification had limited success for NCS-1, as several structural elements, including the unstructured N-terminal region and the dynamic C-terminal H10 helix, exhibited conformational changes upon soaking and fragment binding, making the maps difficult to interpret. 135 datasets showed additional electron density in the NCS-1 hydrophobic crevice or changes in the dynamic C-terminal helix H10. They were selected for additional manual building to improve electron density. Once the N- and C-terminal ends were rebuilt and long PEG chains were modelled and refined, ligands could be identified unambiguously in 49 datasets. Several datasets showed more than one binding event, resulting in a total of 82 modelled fragments (Fig. 3a). Bubble refinements focused on the fragment's environment (10 Å distance) were carried out. The final resolution of the 49 datasets ranged from 1.5 to 2.3 Å, with the majority falling between 1.7 and 1.9 Å (Fig. 3d).

**Fig. 3:**
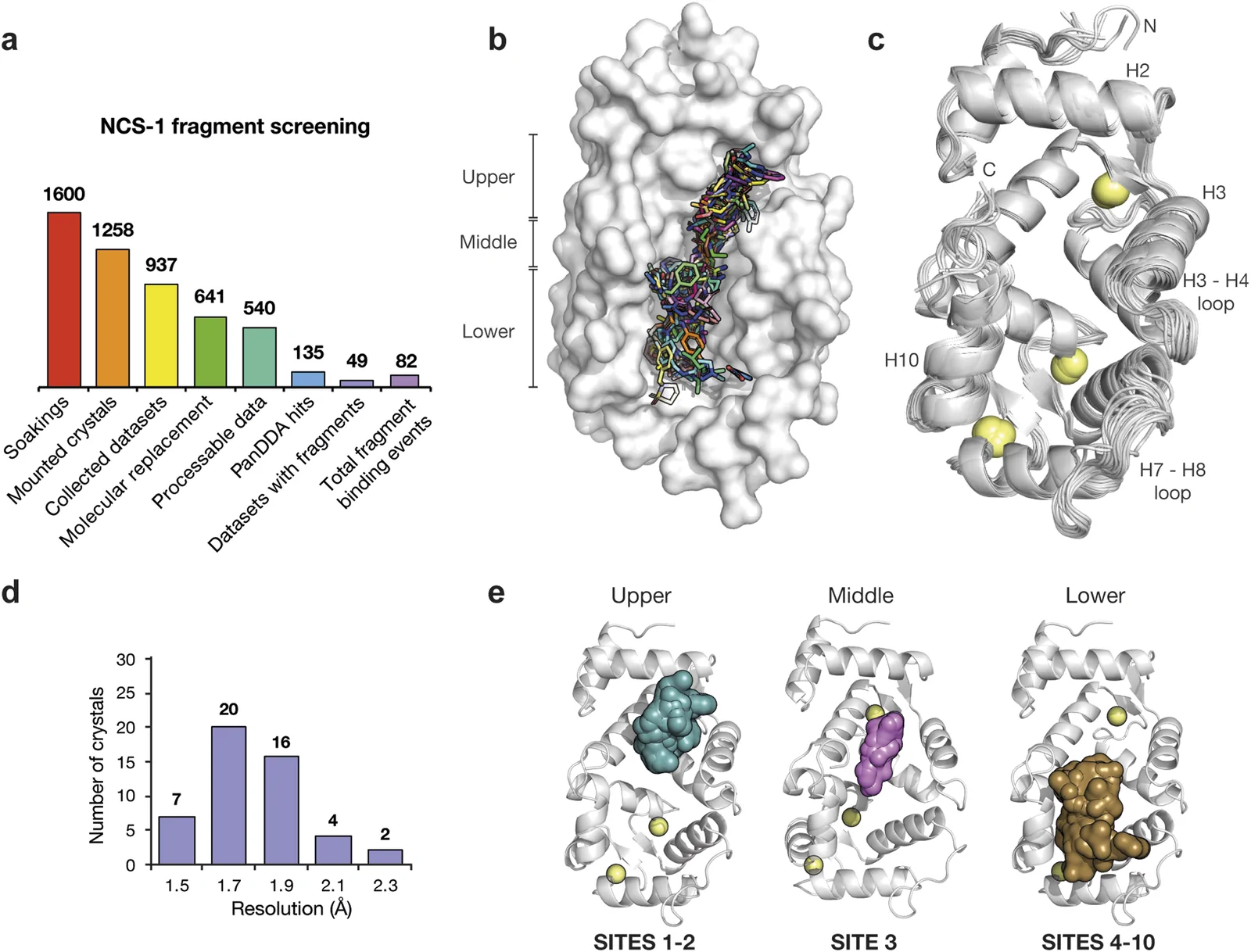
Fragment screening by X-ray crystallography identifies 82 fragments bound to NCS-1. (a) Histogram of the NCS-1 fragment screening process. (b) Representation of the molecular surface of NCS-1 and the superposition of identified fragments (shown as sticks). (c) Superposition of NCS-1 fragment-bound structures. Ca^2+^ ions are shown as yellow spheres. Variable regions are indicated. (d) Histogram displaying the maximum resolution of crystals where fragments were identified. (e) Distribution of fragments along the NCS-1 crevice. Fragments occupying the different regions of the crevice (upper, middle and lower parts) are represented as molecular surfaces in green, pink and brown, respectively. Fragment sites belonging to each region are indicated.

### The analysis of fragment distribution reveals the chemical space of the NCS-1 PPI hydrophobic crevice

An overlay of all fragment hits shows that the entire hydrophobic crevice is occupied by ligands (Fig. 3b). Some datasets revealed up to four distinct binding events along the NCS-1 crevice, while others showed only one. An overlay of the ligand-bound NCS-1 chains highlights regions with the greatest conformational variability upon ligand binding (Fig. 3c). These include the N-terminal region of the protein, the dynamic helix H10 and its C-terminal unstructured tail, the Ca^2+^ binding loop of EF-hand 1 (EF-1), which is insensitive to Ca^2+^ binding, and the loops connecting EF-hands EF-2 and EF-3 (H3–H4 loop) and EF-3 and EF-4 (H7–H8 loop) (Fig. 3c).

Fragments were observed to bind along three main regions of the hydrophobic crevice: the upper, middle, and lower regions (Fig. 3c and e). Based on the residues involved in fragment recognition, ten distinct binding sites were identified. Sites 1 and 2 are in the upper region of the cavity, Site 3 is in the middle region, while Sites 4 to 10 are in the lower region (Fig. 3e).

### Fragments bound to the upper region of the NCS-1 cavity

A total of 20 unique fragments bind in the upper part of the cavity (SI Fig. 1). Site 1 contains eleven unique fragments that interact with helix H2 (SI Fig. 1a). All fragments establish contacts with residue W30 through π–π interactions or hydrophobic van der Waals contacts. Additionally, four fragments form hydrogen bonds with D37, while the remaining ten interact with D37 through van der Waals contacts (SI Fig. 1e). The fragment Z86416893 is of particular interest, as it is strongly anchored to helix H2 through two hydrogen bonds with D37. It also forms π–π interactions with W30 and further van der Waals contacts with nearby residues (SI Fig. 1b). These interactions make this fragment a promising hit for further development. Site 2 contains nine unique fragments that interact with key residues on helices H3 (Y52) and H5 (F85 and T92) (SI Fig. 1c and d). These fragments can be divided into three subgroups based on their interactions: those that form only hydrophobic interactions with F85, those that form a direct hydrogen bond with Y52, and those that establish a direct hydrogen bond with T92 (SI Fig. 1f).

### Fragments bound to the middle region of the NCS-1 cavity

A total of five fragments bind to the middle part of the NCS-1 cavity, which represents Site 3 (SI Fig. 2). In all cases, H-bonds are established with residues Y52 or T92 (SI Fig. 2c), which are considered hotspots in the modulation of the NCS-1/Ric-8A complex.^9,10^ The fragment Z55290386 is considered highly promising as it is strongly stabilized with three hydrogen bonds, one direct bond with Y52 and two water-mediated bonds with T92 (SI Fig. 2b).

### Fragments bound to the lower region of the NCS-1 cavity

A significant proportion of the fragments, a total of 42 fragments, bind to the lower part of the NCS-1 cavity (SI Fig. 3 and 4). This is due to the orientation of helix H10, which generates a deeper cavity in this region. To date, no modulators have been reported to bind to this region. This lower part of the crevice has been subdivided into seven binding sites based on the relative positions of the fragments within this region (SI Fig. 3a–c and 4a–d). Fragments in Site 4 feature an aromatic group that forms hydrophobic contacts or π–π interactions with W103, located in the lower part of the cavity. A total of twenty-eight unique fragments span the cavity (SI Fig. 3a and g). In addition, some fragments form hydrogen bonds with Y52 (Z31723394) or with various residues of helix H10. In contrast, the seven fragments in Site 5 are accommodated laterally; they do not establish H-bonds with helix H10 but make van der Waals contacts with W103 (SI Fig. 3b and h). The six fragments in Site 6 do not interact with W103 (SI Fig. 3c and i), but some form hydrogen bonds with Y108 or R148. The remaining fragments are positioned in Sites 7 to 10, in the deepest region of the NCS-1 cavity (SI Fig. 4). Fragments in Site 7 are placed between Y108 and R148 (SI Fig. 4a and e). Most of these fragments feature a protein-interaction aromatic group and contain amine groups or oxygen atoms to establish H-bonds with Y108 or R148, at the very end of the hydrophobic crevice. Fragments in Site 8 overlap with those in Site 7 (SI Fig. 4b and f); however, these five fragments displace the R148 side chain, opening the lower part of the cavity (SI Fig. 4b). Their interactions are similar to those observed in Site 7. The three fragments in Site 9 are located even deeper in the cavity (SI Fig. 4c and g), while Site 10 is represented by a single fragment that interacts with the dynamic loop connecting EF-3 and EF-4 (SI Fig. 4d and h). This fragment is a thiophene-2-carboxylate, which forms π–π interactions with Y129 and establishes hydrogen bonds, mediated by a water molecule, with residues N134 and V136 (SI Fig. 4d).

### Fragment merging guided by structural analysis of protein–protein interactions generates potential NCS-1 PPI modulators

Before modifying, linking, and merging ligands, it is important to consider the structure and dynamics of NCS-1, as well as protein–protein recognition mechanisms. In this crystal form, NCS-1 positions helix H10 outside the crevice, participating in crystal contacts, which leaves the hydrophobic pocket fully exposed (PDB: 6QI4^12^). However, in solution, helix H10 is dynamic, moving in and out of the cavity, and its position is critical for NCS-1 recognition of its targets.^5,14^ Therefore, the positioning of helix H10, and thus modulation of protein–protein interactions, could be regulated by fragment binding.^6,9,10,12^ Additionally, the structural determinants of NCS-1 target recognition are well-known, such as in the case of D_2_R^14^ and Ric-8A.^5^ Therefore, compounds could be designed to target key interaction sites or hotspots with the aim of designing potential PPI modulators.

The 82 unambiguously modelled fragments were used for a fragment progression strategy based on merging and linking fragment–hit combinations using Fragmenstein,^24,32^ along with the placement of commercially available analogs sourced from multiple vendors (SI, Materials and methods). The high number of fragment hits and their spatial proximity enabled the generation of over 25 000 merged compounds. Given the potential therapeutic application of the resulting compounds in central nervous system disorders, a molecular weight cut-off of 500 Da was applied to the merged compound designs. No additional filters related to drug-like characteristics were applied at this stage. To further prioritize suitable optimized hits, the compounds were filtered, ranked and classified using the following criteria: (i) the root mean square deviation (RMSD) between each merged compound and its progenitor fragments; (ii) the Tanimoto coefficient, to evaluate chemical similarity between merged compounds and fragments; and (iii) the change in theoretical binding free energy (ΔΔ*G*_bind_), calculated using Fragmenstein (SI, Materials and methods).

Next, we analyzed the overlap of the protein–ligand interactions with the protein–protein interactions found in the complexes of NCS-1 with its targets, Ric-8A, the dopamine D_2_ and cannabinoid CB_1_ receptors. For Ric-8A and the dopamine D_2_ receptor, the crystallographic structures of protein–peptide complexes were available,^5,6,14^ while in the case of the cannabinoid CB_1_ receptor, an AlphaFold2-Multimer^15,16^ model was generated using available information on the CB_1_R region that is involved in NCS-1 recognition (Fig. 1a).^7,8^

Key NCS-1 amino acids involved in the recognition of D_2_R, Ric-8A and CB_1_R were identified (Fig. 1a). For potential NCS-1/D_2_R modulators, hydrophobic contacts with residues W30, L89 and I51 were considered, as well as interactions with D37, Y52 and Q181 for their ability to establish H-bonds.^14^ The structures of previous NCS-1/D_2_R PPI modulators were also considered, and K100, involved in the recognition of vilazodone and atorvastatin, was selected as a potential residue to target.^6^ For the NCS-1/Ric-8A interaction, classification criteria included hydrophobic contacts with W30 and hydrogen bonds with Y52 and T92 in the middle part of the crevice.^5^ Additional interactions with the C-terminal helix H10 were also evaluated to identify potential Ric-8A inhibitors as previously reported.^2,10^ In the case of Ric-8A stabilizers, polar groups introduced in the middle of the crevice were considered essential for enhancing the PPI, as previously shown.^12^ For potential NCS-1/CB_1_R modulators, residues Y52, Y108, R148, and R151, located in the middle and lower parts of the crevice were considered. Although the predicted NCS-1/CB_1_R complex suggested that CB_1_R recognition spans from the top to the bottom of the hydrophobic crevice, it was decided to concentrate on the interactions observed in the lower part of the crevice as a strategy to explore a region that is less represented in the compounds prioritized for Ric-8A and D_2_R modulation. We hypothesized that this may also contribute to achieving greater selectivity among different NCS-1 PPIs (Fig. 1).

Following these criteria in an iterative manner, the compounds were ultimately shortlisted through visual inspection. The fragments that bound to the upper region of the NCS-1 crevice (Sites 1 and 2) were classified as potential regulators of the NCS-1/D_2_R interaction. Modulators of the NCS-1/Ric-8A interaction were hypothesized to bind to the upper and middle parts of the crevice (Sites 1 to 5). For the regulation of the NCS-1/CB_1_R interaction, the fragments placed at Sites 5 to 10 were selected, since they occupy the lower part of the NCS-1 crevice and interact in the surroundings of Y108 and R148 (Fig. 1). Furthermore, fragments that bound to the loop connecting EF-hands EF-3 and EF-4 or in the surrounding area were selected, as this region is relevant for target recognition, is not conserved in the family of Neuronal Calcium Sensors and has not been explored before.

This structural analysis not only enabled the selection of sites for fragment pairing but also established a priority list of 66 prioritized mergers. Of these, 17 could be potential D_2_R modulators, 16 Ric-8A inhibitors, 16 Ric-8A stabilizers, and 13 CB_1_R modulators. Finally, there were 4 ligands that bound to the dynamic loop connecting EF-hands EF-3 and EF-4 that were selected.

17 out of the 66 compounds were commercially available and were purchased directly from Enamine using a €2.6k budget (SI Table 1). They were chemically diverse and, based on their predicted positions within the binding pocket, could potentially modulate different NCS-1 targets. Five, eight and four potential modulators of D_2_R, Ric-8A, and CB_1_R, respectively, were chosen (SI Table 1 and Fig. 5).

### Algorithmic methods generate combinations compatible with robotic synthesis and SAR studies

The FFF pipeline consists of several algorithmic and digitized tools to enable fragment progression:

First, “Synthetically Directed Elaborations” (Syndirella) (https://github.com/oxpig/syndirella) enumerates the SAR space around designed scaffolds.^25^ Syndirella's modular design allows the user to restrict elaborations to reactions within their own synthetic repertoire and to specify preferred reactions (*e.g.* amidation over ester amidation) and reactants (*e.g.* boronic esters over boronic acids), making it adaptable to any high-throughput synthesis platform and to future synthetic approaches. Syndirella also ensures that the selected compounds are suitable for CAR (Chemist Assisted Robotics) robotic synthesis.

Second, “Hit Interaction Profiling for Procurement Optimization” (HIPPO) (https://github.com/mwinokan/HIPPO) is a down-selection tool that helps select the next “best” experiment as a set of elaborated compounds generated by Syndirella. HIPPO is a multi-parameter sampling tool. In this work, the percentage of recapitulated fragment interactions and the number of additional interactions were used as scoring parameters. HIPPO has been intentionally engineered to be parameter agnostic, allowing different parameters and sampling approaches to be accommodated in future fragment progression projects.

Third, “Chemist Assisted Robotics” (CAR) is a digitized and database-driven chemistry platform that facilitates multi-step high-throughput synthesis of the compounds down-selected by HIPPO. Digitized machine and human readable chemistry protocols are the heart of CAR; these protocols are flexible and allow the chemist to use different reaction parameters and configure the order of protocol execution. The digitized protocols are separated into human and machine executable “action sessions” (reaction, stir, workup, and analyze) and, within each action session, a set of “actions” (add, extract and mix) that enable the chemist to configure the desired protocol sequence. A database is CAR's engine that converts digitized protocols into reactions grouped, depending on the reactor labware available, by total reaction volume and temperature into reactor plates.

The recently developed algorithmic method Syndirella^25^ was employed to generate synthetically tractable elaborations from mergers or “scaffolds” that could be used for SAR (Structure–Activity Relationship) studies of the NCS-1 PPI.

Initially, the previously selected 66 base compounds were considered. Based on factors such as reaction selectivity, molecule growth vectors, and the cost of starting materials, 12 base compounds were ultimately selected as scaffolds for design space and input into Syndirella. These 12 scaffolds were processed using Syndirella and produced elaborated compounds designed to broadly explore the chemical and structural space of the NCS-1 hydrophobic crevice, while limiting the chemical synthesis to a single step. The proposed reactions included amidation, Schotten–Baumann sulfonamidation, Williamson synthesis, Suzuki reactions, and Chan–Lam coupling (SI, Materials and methods).

Over 5000 elaborations were analyzed with HIPPO, and a set of compounds was down-selected that maximized the yield from the starting materials within a fixed budget and maximum lead time of four weeks for receiving reactants. Elaborations from six scaffolds selected were named D2R-5, D2R-12, CB1R-5, Ric8inhib-1, Ric8inhib-3, and Ric8enhan-3; based on the PPI they were hypothesized to modulate (Fig. 4, SI Fig. 6 and 7). The synthesis of all compounds was designed to proceed *via* a single synthetic step, primarily through an amidation reaction. An exception is Ric8enhan-3, which was synthesized using Schotten–Baumann sulfonamidation with amines (Table 1). Fig. 4 illustrates the chemical space of the NCS-1 PPI explored by the proposed base compounds and the elaborations designed, within a budget of €8000 to purchase the starting materials. The selected collection of starting materials included the generation of 590 products from 280 starting materials. While the six base compounds cover 38.5% of the fragment screening interactions, the 590 products recapitulate 100% of the fragment interactions and introduce 241 new interactions (Fig. 4).

**Fig. 4:**
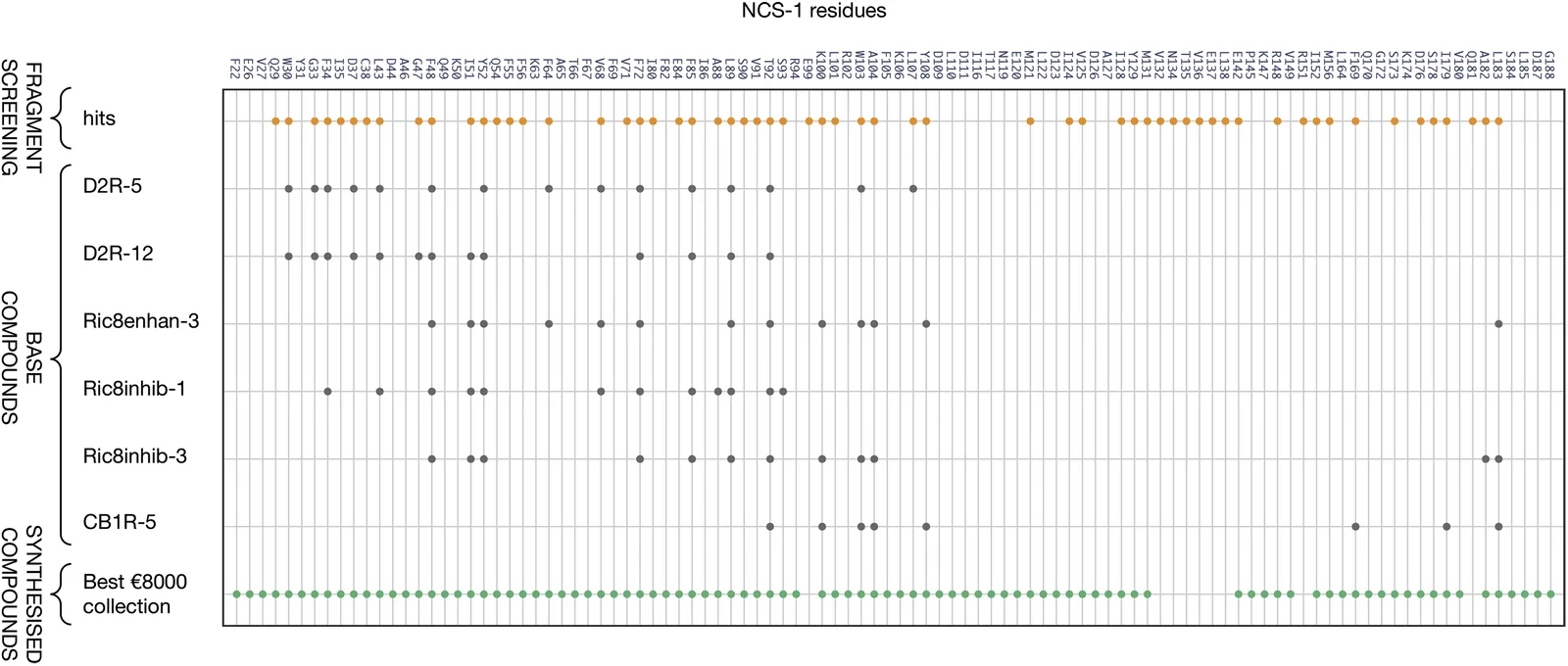
Exploration of the chemical space of the NCS-1 PPI. Observed interactions in the NCS-1 fragment screening (hits; orange circles) with each NCS-1 residue. Predicted interactions for the six base compounds and the chosen collection of elaborations designed around the base compounds, suggested by Syndirella and adjusted to the €8000 reactant budget using HIPPO.

**Table 1:** Selected base compounds for elaboration and synthesis *via* CAR. The table shows the fragments that inspired the elaboration, predicted data from Fragmenstein and HIPPO, the synthesis summary, the products detected using MS and the number of crystal structures solved from crude extracts. (S) indicates the site where the fragments of inspiration are found in the fragment screening

	Base compound (Syndirella)					
	D2R-5	D2R-12	CB1R-5	Ric8inhib-1	Ric8inhib-3	Ric8enhan-3
Fragment inspiration	Z86416893 (S1) + Z239127534 (S3)	Z53834613 (S1) + Z198195770 (S2)	Z1265813904 (S4) + Z1272480091 (S6)	Z26333448 (S2) + Z1265813904 (S3)	Z1265813904 (S3) + Z1198177230 (S4)	Z802824098 (S3) + Z1269220427 (S4)
Synthesis reaction	HATU amidation	HATU amidation	HATU amidation	HATU amidation	HATU amidation	Schotten–Baumann sulfonamidation with amine

**Fragmenstein**						
ΔΔ*G* (kcal mol^−1^)	−11.0	−9.5	−11.7	−9.2	−8.2	−11.7
RMSD (Å)	0.99	0.83	0.45	0.95	0.83	0.45
Tanimoto	0.19	0.22	0.46	0.25	0.28	0.46

**Prediction of key interactions**						
W30	✗	✗				
D37	✗	✗				
Y52		✗		✗	✗	
T92		✗		✗		✗
K100			✗		✗	✗
W103			✗		✗	✗
Y108			✗			✗
R148			✗			
Predicted elaborations (HIPPO)	2	60	21	450	18	39
Synthesized elaborations (CAR)	0	27	14	383	14	16
Automated detection of products (MSCheck)	—	5	0	222	10	15
Synthesis success (%)	—	18.5	0	58.0	71.4	93.8
Crystal structures	—	0	0	9	2	3

### New fragment-based compounds are efficiently generated *via* robotic array synthesis and validation

The automated synthesis of compounds was carried out in four different batches (Fig. 5). Of the 590 reactions initially selected by HIPPO, 454 reactions were attempted, representing 76.9% of the collection (Table 1). Very importantly, robotic array synthesis represented a limited investment: €8000 to purchase the starting materials and an additional €3053 to cover the in-house synthesis costs (including technician salary, lab utilities, consumables, and solvent), as estimated by Fieseler *et al.*^25^ Therefore, bringing the total cost to approximately €11 000.

**Fig. 5:**
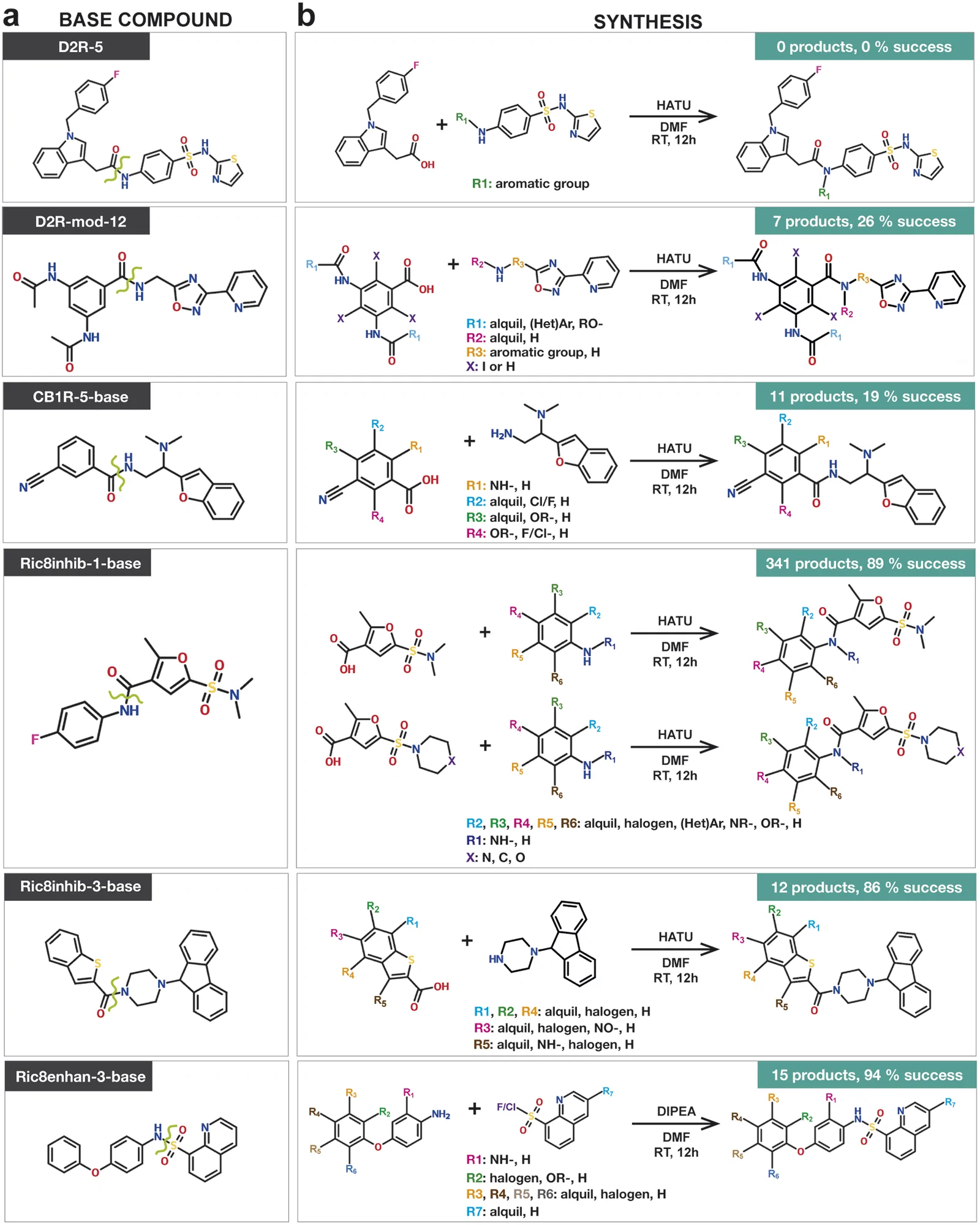
Fragment-based compound synthesis *via* CAR. (a) 2D structures of the base compounds used for chemical elaboration. The green curve shows the bond created after merging the two starting molecules. (b) Synthesis and reaction conditions for each base compound. The starting materials are displayed along with the various functional groups (R*n*, *n* = 1–7). For each panel, the number of elaborated products and the success rate validated *via* LC-MS are indicated in the green box.

Among the executed reactions, elaborations based on Ric-8A-targeting series (Ric-8inhib-1, Ric8inhib-3 and Ric-8enhan-3) dominated the library, accounting for 95.3% of the total elaborations. This prevalence is due to two main factors: first, the starting materials for Ric-8A series elaborations were more affordable, and second, there was a broader commercial availability of starting compounds, allowing more combinatorial pairing. In contrast, the design space size that was used by the amidation reactions (CB_1_- and D_2_R-targeting series) was constrained as D_2_R-targeted base designs required starting compounds that were unavailable at the time of purchase.

After synthesis, mass spectrometry was used to analyze the crude reaction mixtures. To facilitate the time-consuming task of analyzing hundreds of chromatograms, an automated LC-MS analysis tool called MSCheck^33^ was employed. In summary, MSCheck detected the presence of 15 sulfonamides (93.8% matches) and 237 amides (54.1% matches) by matching masses detected by scanning both positive and negative modes to the appropriate parent ion (Table 1 and Fig. 5). Generalized reaction protocols were applied for both the sulfonamide and amidation reactions, where sample preparation for scheduled beamtime was the overriding driver *vs.* exploring more reaction conditions to improve synthesis success rates.

### Grating-coupled interferometry facilitates high-throughput characterization of compound binding in solution

Grating-coupled interferometry (GCI) has been established at XChem as the orthogonal method for high-throughput determination of binding affinities. We aimed to establish a protocol to select the best binders from crude reaction mixtures, avoiding time-consuming purification step of hundreds of synthesized products and speeding up hit optimization stages. To detect the binding from impure and complex mixtures, a robust protocol was established first with purchased pure compounds (E1–E17) that served as controls. Six compounds (E6, E10, E12, E14, E16, and E17) were detected as NCS-1 binders, representing 35% of the total compounds tested (Fig. 6a and SI Table 1). Under the experimental conditions, these compounds exhibited affinities in the micromolar range (≈60–140 µM; Fig. 6a and SI Table S12). Compounds E3 and E4 exhibited non-specific binding to the NTA surface, and the remaining compounds showed no detectable response. An orthogonal intrinsic tryptophan emission fluorescence assay was used to cross-validate the GCI results (SI Fig. 10). Agreement between the two techniques demonstrates that the immobilization of NCS-1 through a N-terminal 6xHis tag has no penalty on affinity measurement (SI Table 2).

**Fig. 6:**
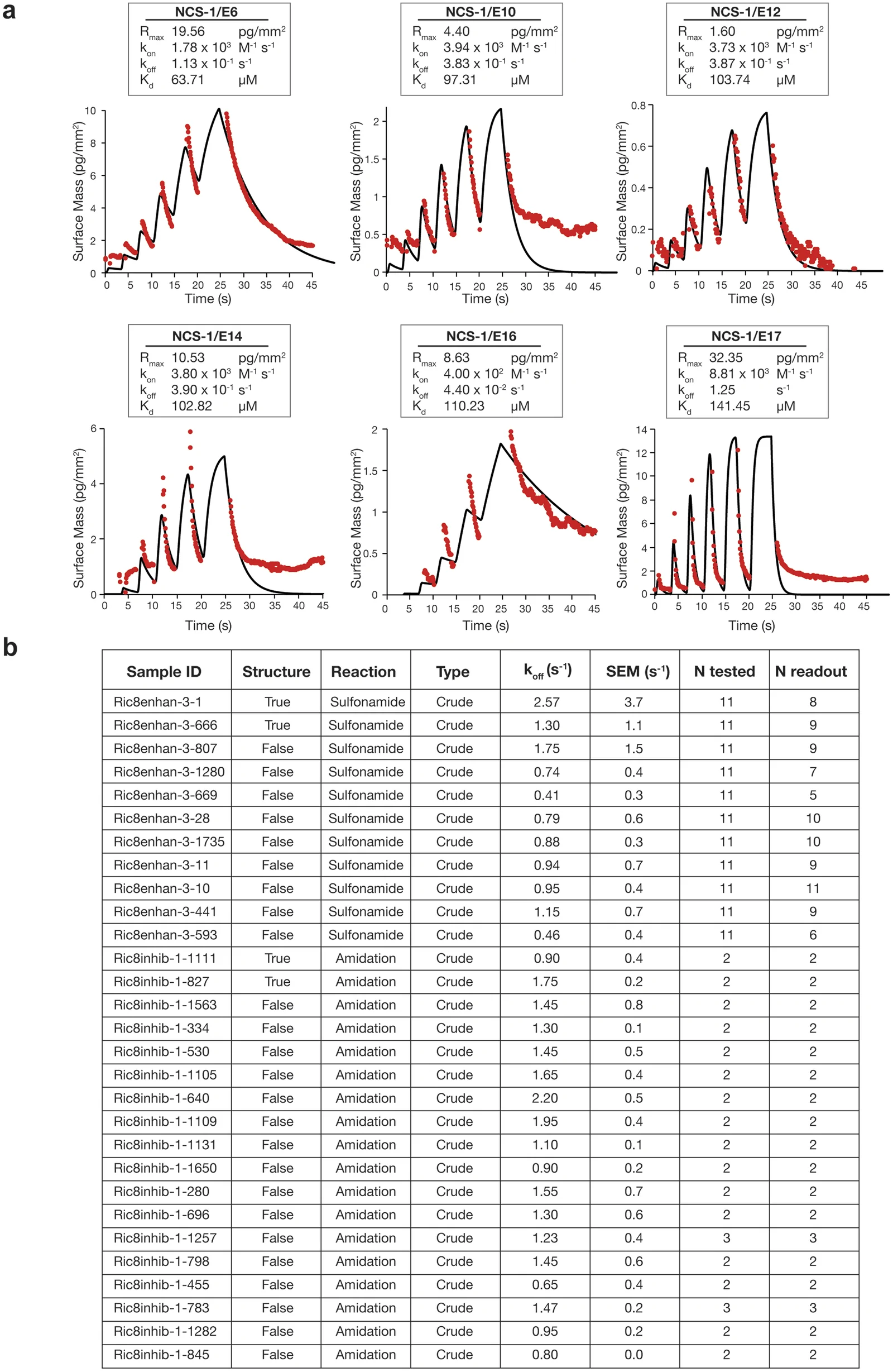
Study of the interactions between pure compounds and NCS-1 using grating-coupled interferometry (GCI). (a) Sensograms for pure compounds. Raw data are represented as red circles, with their respective fits in black. The binding kinetics were fitted to a 1 : 1 binding model. The tables summarize the kinetic parameters for each ligand: *R*_max_ (surface density of captured protein), *k*_on_ (association rate constant), *k*_off_ (dissociation rate constant), and *K*_d_ (dissociation constant). (b) Measurement of compounds from crude reaction mixtures. For each ligand, sample ID, crystal structure, reaction, assay, *k*_off_ data and SEM (standard error of the mean) are indicated.

As previously mentioned, the primary goal of implementing this technique is to detect interactions of impure compounds from reaction mixtures. The sample concentration is needed to obtain affinity/kinetic information through the SPR or GCI strategy. Since the concentration in these mixtures is unknown, dissociation rate constants (*k*_off_) can be used for the detection of binders.^34,35^ The off-rate screen is evaluated by fitting the dissociation phase for the ligand and sample interaction. The dissociation decay is independent of the sample concentration. The adjustment step was applied similarly to that used for the pure Enamine samples. Fitting parameters were adjusted based on the strength of the interaction between the sample and the target. Compound E17, with an affinity (*K*_D_) of 140 µM and a dissociation rate constant (*k*_off_) of 1.25 s^−1^, was used as a control (Fig. 6a). In this study, the control compound E17 and other crude reaction mixtures have a fast dissociation profile. For this reason, the dissociation fit point was adjusted to 26.5 seconds. Among the 252 crude reaction mixtures tested, 15 samples indicated a repeatable binding response and *k*_d_ values in the 1–2 s^−1^ range (Fig. 6b). Of these, two compounds from the amidation series (Ric8inhib-1-1111 and Ric8inhib-1-827) and two from the sulfonamide series (Ric8enhan-3-1 and Ric8enhan-3-666) yielded structure-binding response pairs (Fig. 6b).

### Crystallographic screening of elaborated compounds from pure and crude reaction mixtures

Previous studies have demonstrated that crude reaction mixtures containing both starting materials and products can be directly soaked into crystal drops, enabling structure determination of synthetic products bound to the target protein without prior purification.^22^ Building on this premise, CAR crude reaction mixtures were solvent-exchanged into DMSO and adjusted to a nominal concentration of 100 mM (SI, Materials and methods). Soaking experiments were performed using the same protocol as for fragment screening, reaching a final DMSO concentration of 10%, corresponding to a theoretical maximum ligand concentration of 10 mM, assuming complete product formation and solubilization. An initial 2 h soak yielded a single ligand-bound structure; therefore, longer incubation times (8–18 h) were subsequently employed, resulting in 13 ligand-bound structures from 252 LC-MS-positive crude mixtures. In addition, soaking of pure compounds yielded two structures of NCS-1 in complex with E1 and E14.

In total, the soaking campaign produced 16 structures: 14 derived from CAR synthesis products and 2 from the pure compounds (Fig. 7 and SI Fig. 8). Both 2*F*_o_ − *F*_c_ and *F*_o_ − *F*_c_ electron density maps indicated the presence of ligands corresponding to the synthesized products *via* CAR occupying the NCS-1 hydrophobic crevice (SI Fig. 9). With the exception of Ric8inhib-1-1111, where two ligands were modelled within a single NCS-1 molecule, all structures showed 1 : 1 stoichiometry. In some datasets, the quality of the density only allowed modelling of the ligand in one of the independent NCS-1 molecules; where density was present in both molecules, the corresponding poses were essentially identical, except for E1, which adopted distinct poses in different regions of the elongated crevice (Fig. 7 and SI Fig. 8 and 9).

**Fig. 7:**
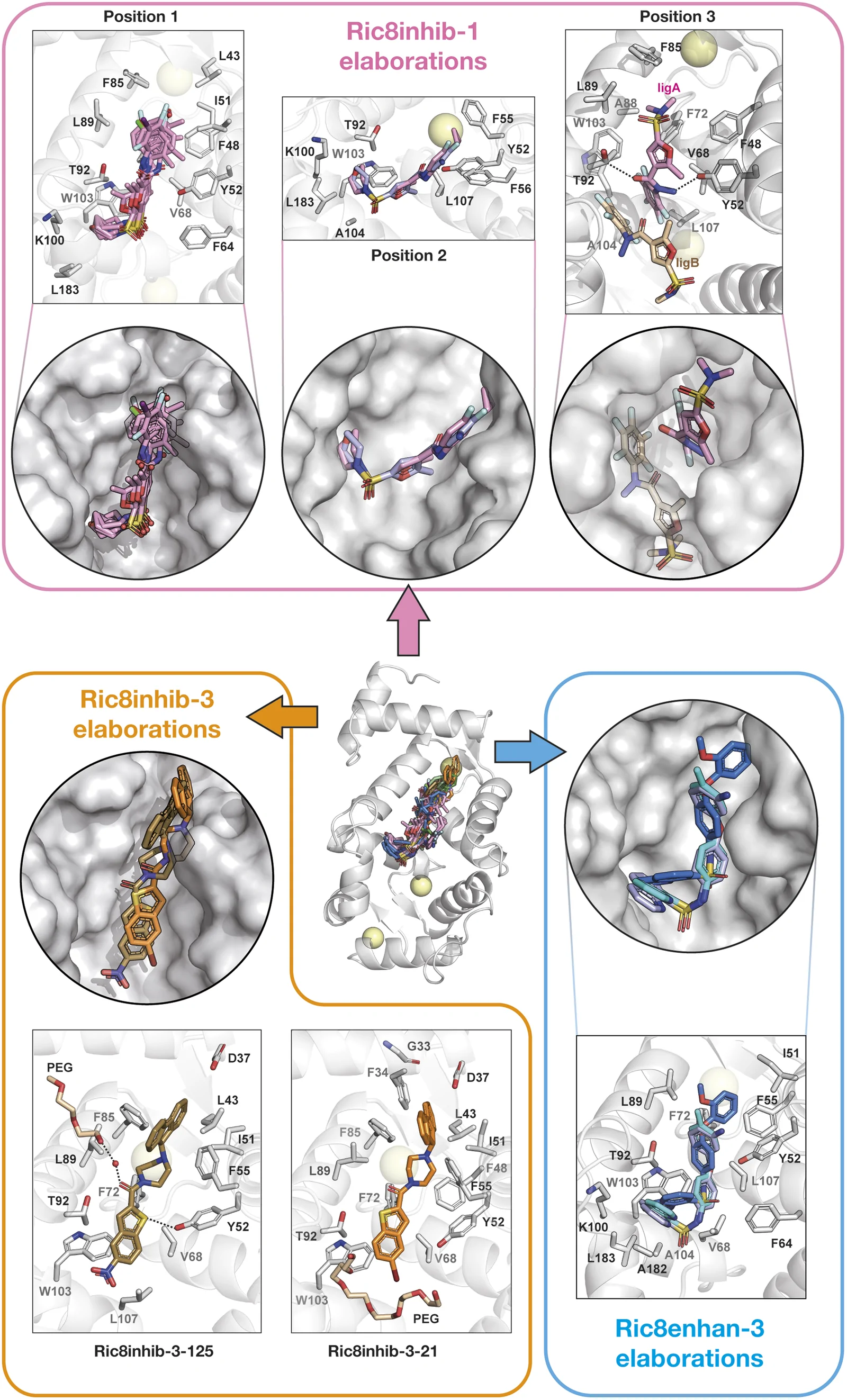
Screening of larger compounds *via* X-ray crystallography. In the center, the structure of NCS-1 (ribbons) in complex with all the crystallized compounds (sticks), which have been superimposed. A magnification of the crystallized compounds Ric8inhib-1 (pink), Ric8inhib-3 (orange) and Ric8enhan-3 (blue) is displayed in each box. The NCS-1 cavity where ligands are located, together with details on the interacting residues, is also shown.

The CAR-derived structures fall into three scaffold series designed to modulate the interaction between NCS-1 and Ric-8A (SI Fig. 7 and Table 1). The elaborations are located at the top and central region of the hydrophobic crevice of NCS-1 (Fig. 7 and SI Fig. 8), distributed across the fragment Sites 1 to 5 (SI Fig. 1, 2 and 3).

### Structural analysis of elaborated compounds

#### Ric8inhib-1-based elaborations

Nine structures were obtained (Table 1 ,Fig. 7 and SI Fig. 8). All elaborations share a 4-fluorophenyl-2-methyl-1-sulfonyl-3-furamide core (SI Fig. 8b), differing in (i) substituents on the 4-fluorophenyl ring at one end of the ligand and (ii) the sulfonyl substituent, which is either a dimethylamine or a non-aromatic nitrogen heterocycle (SI Fig. 8b). Six elaborations adopt a pose in the intermediate region of the NCS-1 cavity (Position 1; Fig. 7), where the amide group establishes key contacts with Y52 and T92 (SI Fig. 8b). These six ligands feature a heterocycle attached to the sulfonyl group at the lower part of the molecule. By contrast, no structures were obtained for dimethylamine-substituted analogs in the same orientation (Fig. 7). This trend is consistent with the heterocycle contributing to productive binding. At the top, the aromatic ring carries *ortho*-, *meta*-, or *para*-substituents, forming hydrophobic contacts with neighboring residues in the cavity (Fig. 7). The different substituents confer chemical diversity and allow the exploration of interactions with residues in the NCS-1 crevice. In addition, three elaborations were found to occupy other sites, termed Positions 2 and 3 (Fig. 7). Two ligands, Ric8inhib-1-1463 and Ric8inhib-1-518, were located at Position 2, adopting a more solvent-exposed pose and engaging predominantly hydrophobic contacts with aromatic residues toward the base of the cavity (Fig. 7). A third ligand, Ric8inhib-1-1111, adopts “Position 3” and displays a distinct chemical pattern: it features a dimethylamine group and a substituted amine on the furamide ring. Notably, two molecules were found in the cavity, establishing contact between them. The upper molecule establishes interactions in the central region and adopts an orientation approximately opposite to that observed for Position 1 elaborations, while the lower molecule sits deeper in the cavity. The two molecules contact each other *via* face-to-face interactions consistent with π–π stacking between their furamide rings (Fig. 7). Among the Ric8inhib-1 elaborations, Ric8inhib-1-1287 is of particular interest, as it forms H-bonds with Y52 and T92 (Table 2), two residues that constitute key hotspots in the NCS-1/Ric-8A interface. This ligand also exhibits a comparatively large interaction footprint (BSA and contact metrics; Table 2). Conversely, the substitution of morpholine or piperidine groups with a pyrrolidine (Ric8inhib-1-655) results in a lower Δ*G* value, indicating the importance of these groups for the interaction (Fig. 7 and Table 2).

**Table 2:** Analysis of the interactions found in the NCS-1 elaboration-bound structures. Buried surface area (BSA) and estimated solvation free-energy gain upon complex formation (Δ*G*) were calculated using the PISA server.^36^ Protein–ligand contacts and interacting residues were identified using NCONT from the CCP4 package with a 4.2 Å distance cutoff^37^ and LigPlot+.^38^ The number of residues establishing hydrophobic contacts, H-bonds and water-mediated H-bonds are indicated for each follow-up crystal structure. High-interest compounds are marked in green

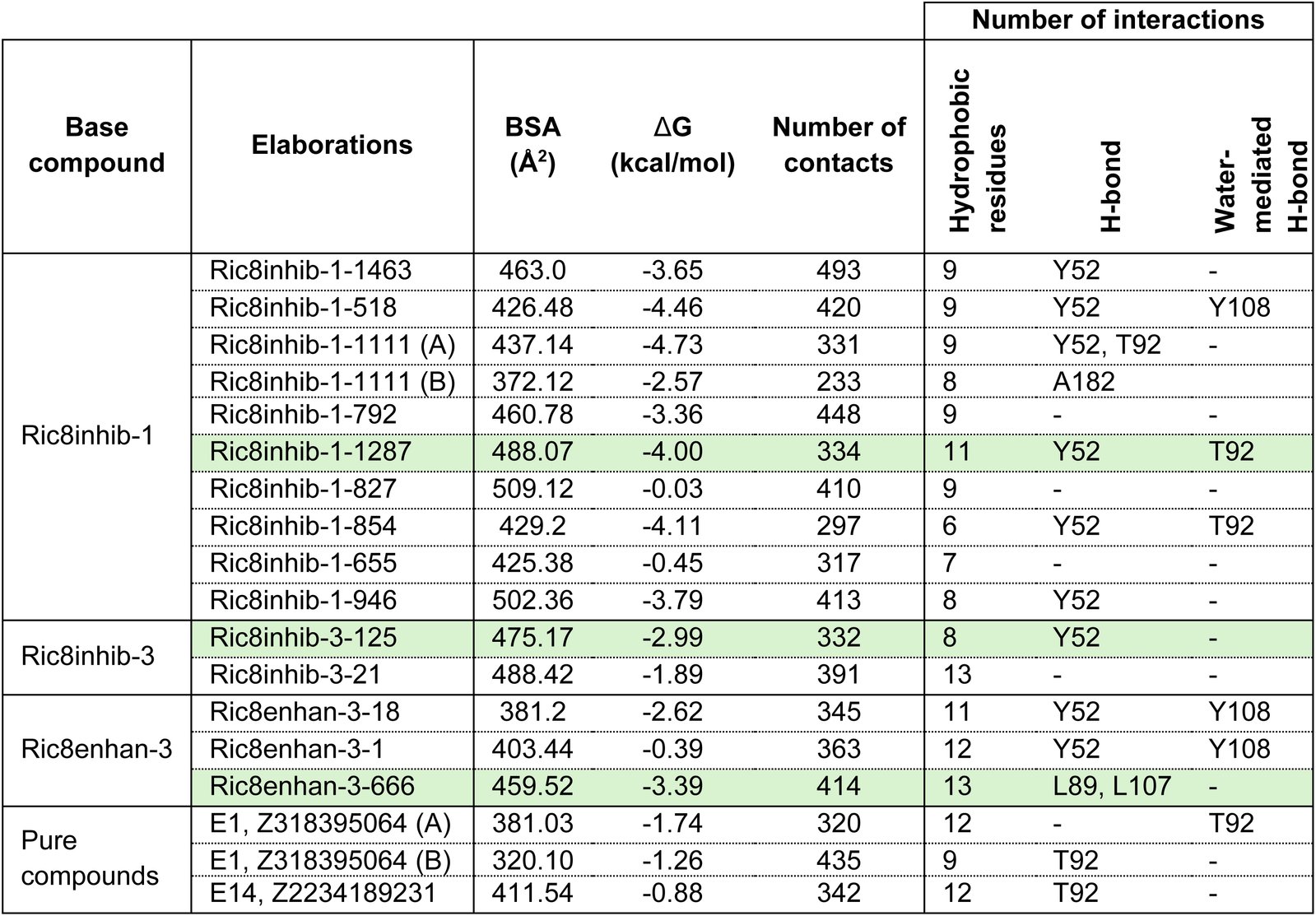

#### Ric8inhib-3-based elaborations

Two Ric8inhib-3-derived structures were solved, with both ligands bound in the upper half of the NCS-1 crevice (Fig. 7). These elaborations share a common architecture comprising a tricyclic fluorene moiety, a piperidine group and a benzothiophene substituent (Fig. 7). One molecule is the enantiomer of the other, suggesting that NCS-1 does not differentiate between them. In Ric8inhib-3-125, the benzothiophene sulfur forms a H-bond with Y52, whereas in Ric8inhib-3-21, the sulfur points toward the opposite side of the cavity, establishing van der Waals contacts with T92 (Fig. 7). Both ligands are stabilized by extensive aromatic interactions, including π–π contacts with neighboring aromatic residues in the upper crevice (Fig. 7). Consistent with these interaction patterns, Ric8inhib-3-125 shows more favorable interaction metrics in Table 2 and emerges as a promising starting point for further optimization.

#### Ric8enhan-3-based elaborations

Three structures were solved. Ligands are located in the middle region of the NCS-1 cavity (Fig. 7). These elaborations share a sulfonamide-based scaffold with a quinoline group on one side (Fig. 7). Elaborations feature a benzene group with substituents at *ortho*-, *meta*-and *para*- positions at the upper part of the cavity (Fig. 7). In all cases, ligands form H-bonds with Y52 in the middle of the crevice. Notably, Ric8enhan-3-666 forms an additional H-bond with L89 at the top of the crevice. It also displays the largest protein–ligand contact area within the series, with a higher number of interactions. This is consistent with its more favorable Δ*G* (Table 2). It is noteworthy that similar compounds containing an extra methyl group bound to the quinoline moiety were synthesized (Fig. 5). None of these derivatives yielded structures, suggesting that the presence of the methyl group may destabilize the interaction with NCS-1.

#### Pure elaborations

The structures of NCS-1 in complex with the E1 and E14 purchased compounds were solved. These molecules bind in the upper half of the NCS-1 crevice, establishing H-bonds with residues in the middle part of the crevice and van der Waals contacts (SI Fig. 8 and Table 2). E14 showed an affinity in the range of tens to hundreds of micromolar (Fig. 6a and SI Table 2). In the case of E1, while no affinity was detected under the experimental conditions tested, different binding modes were found when analyzing each independent NCS-1 molecule. The variable poses identified for E1 may suggest certain lack of specificity due to the weak binding, which was not detected with any of the biophysical techniques. The high concentrations used in the soakings allow the structural resolution of weak binders, as in the case of fragments. Lastly, it is important to note that, although only two structures of pure compounds were solved, these results come from a single crystal soaking experiment. Given the affinity shown for E6 and E12, it would have been advisable to repeat soaking with these compounds.

### Analysis of the pose divergence between fragment hits and larger elaborations

Overall, the elaboration binding modes have partial overlap with the inspiration fragment binding modes and show limited agreement with the predicted binding modes (SI Fig. S13 and S14). The median RMSD to the predicted binding modes was 4.60 Å, indicating substantial divergence. To assess whether large RMSDs still preserve the same interactions, we compared ProLIF computed interaction fingerprints between predicted and experimentally resolved binding modes (SI, Materials and methods).^39^ Across the set, the majority of compounds, 9 out of 16 compounds, showed zero shared interactions, while 7 out of 16 retained ≥1 shared interaction. These large divergences can be due to multiple reasons. Upon analysis of the lowest and highest average RMSD values, the three compounds with the lowest values were all part of the Ric8enhan-3 series and strongly recapitulated the binding mode of the x0125-0A inspiration fragment. Notably, x0125 was resolved twice with highly similar poses, suggesting a consistent and reproducible binding mode that these compounds adopt. The three compounds with the highest average RMSD, E14, Ric8inhib-3-21 and Ric8inhib-3-125, were rotated ∼180° and shifted relative to their inspiration fragments. All three were inspired by x0119, a fragment that was resolved in three distinct binding modes across the site. Although E14 was placed by Fragmenstein relative to x0119-0B, the experimentally resolved structure showed better spatial overlap with x119-2B. Additionally, Ric8inhib-3-21 matched an interaction with an alternative x0119 mode (x0119-1B; Ile51 van der Waals contact), rather than any interaction with the x0119-2B pose used for placement. This suggests that compounds derived from fragments with multiple binding modes may resolve matching alternative fragment poses rather than the specific mode used during placement.

### The implemented workflow delivers compounds with PPI modulatory activity and translational relevance

To test whether the developed workflow can deliver biologically relevant compounds, we tested if compound E14, for which both affinity and structural data were obtained, can modulate NCS-1 mediated PPI interactions. Despite the different chemical nature, the interaction pattern of E14 and NCS-1 is similar to that found for a previously discovered NCS-1/Ric-8A enhancer called 3b (Fig. 1b and SI Fig. S15).^12^ Therefore, modulation of this PPI was tested. E14 stabilized the interaction of the NCS-1/Ric-8A complex in a co-immunoprecipitation-based protein–protein interaction assay in HEK cells (Fig. 8a). To assess PPI modulation under more physiological conditions, we used the Proximity Ligation Assay (PLA) technology. PLA allows the evaluation of protein–protein interactions *in situ* with high sensitivity;^40^ it has been used previously to study NCS-1 mediated PPI modulation.^6,13^ This technology is based on the specific antibody recognition of the two proteins of interest and takes advantage of DNA primers covalently linked to secondary antibodies. Only when the complex is formed do probe primers bind, and amplification and fluorescent detection are possible. Flow cytometry analysis of fluorescence intensity per cell revealed an increase in the number of cells with high fluorescent content due to E14, as shown in the uniparameter histograms (Fig. 8b). The average fluorescence from three independent flow cytometry PLA experiments revealed a 35% increase in the average fluorescent intensity in the cells treated with E14 compared with cells treated with DMSO, showing its PPI stabilizing properties in cellulo. This positions the compound as a candidate for hit-to-lead development in neurodegenerative diseases, since the stabilization of the NCS-1/Ric-8A complex restores synapse number in an Alzheimer's disease model.^12^

**Fig. 8:**
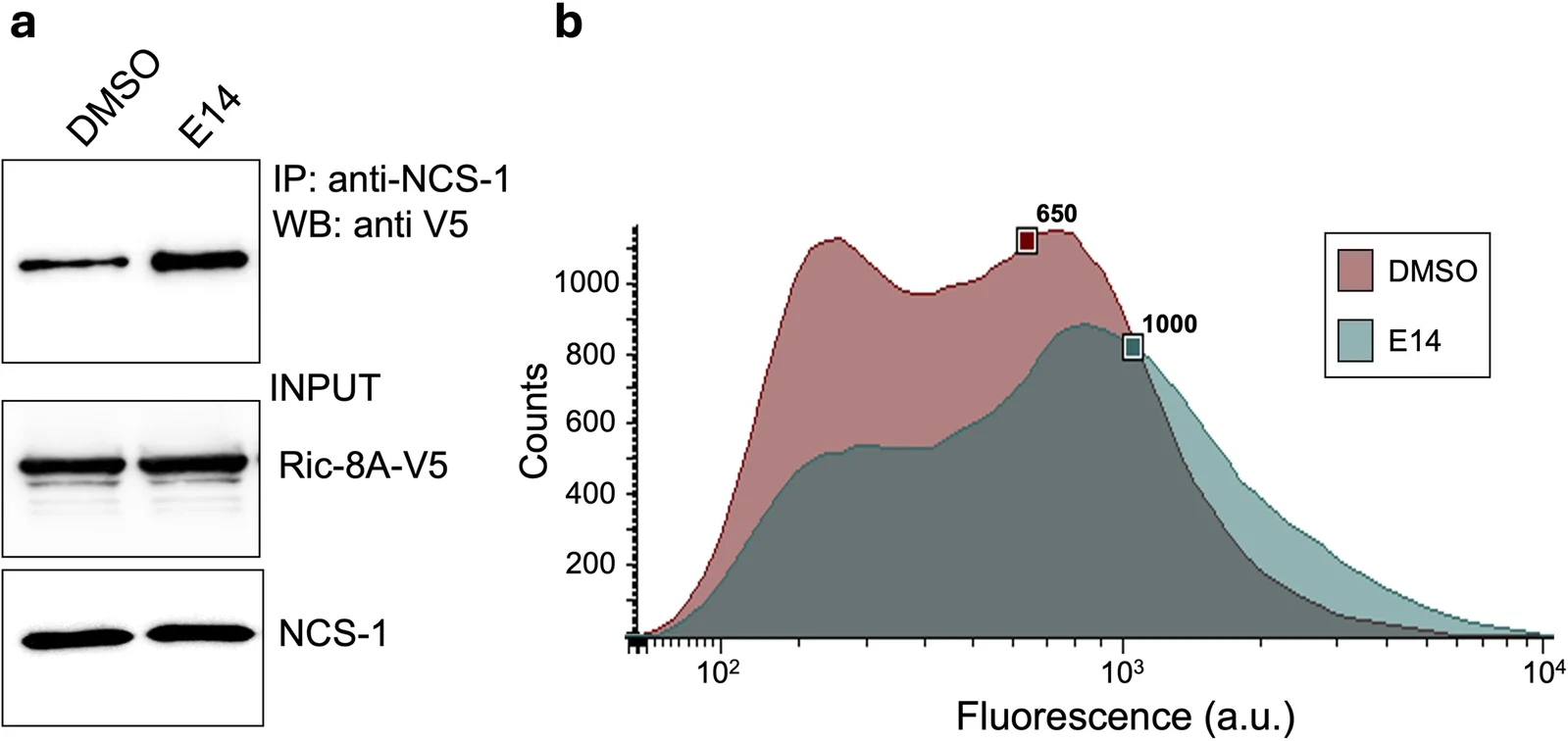
E14 is a modulator of the NCS-1/Ric-8A interaction. (a) Representative co-IP assay from HEK293 cells showing that the binding of E14 enhances NCS-1/Ric-8A formation. (b) PLA analysis of NCS-1/Ric-8A complex formation *in situ*. Representative uniparametric histogram (counts *vs.* green fluorescence). Mean fluorescence values (a.u.) are indicated for DMSO- and E14-treated conditions.

Beyond E14, several CAR-derived elaborations show interaction patterns that suggest potential PPI modulatory activity, since they directly interact with relevant residues implicated in Ric-8A, D_2_R and CB_1_R, such as Y52, T92 and the hydrophobic residues that surround these amino acids (Fig. 1a and SI Fig. S8), and they partially overlap with previously found Ric-8A and D_2_R modulators (Fig. 1b and c).^6,9,12,41^ In particular, the interaction pattern observed for Ric8inhib-1-1287, Ric8inhib-3-125, and Ric8enhan-3-666 (Table 2), justify follow-up studies with purified material to quantify binding affinity and PPI modulation of NCS-1 protein targets.

In addition, *in silico* property assessment of the 16 crystallized elaborations indicates an absence of PAINS substructures and general compliance with Lipinski's guidelines, supporting their suitability as starting points for hit-to-lead development. However, several compounds trigger Brenk alerts, which may reflect potential liabilities (*e.g.*, reactivity or toxicity) and should be considered during optimisation: Ric8inhib-1-1287, Ric8inhib-1-854, Ric8inhib-1-1111, Ric8enhan-3-1, and Ric8inhib-3-125. Blood–brain barrier (BBB) penetration predictions suggest that only Ric8inhib-3-21 is likely to cross the blood–brain barrier.

Because NCS-1 is a pharmacologically relevant target of the central nervous system, effective modulators require BBB penetration and therefore typically show a molecular mass below 400–500 Da.^42^ Beyond the fragment merging/linking strategy that we have implemented, some fragments already exhibit molecular mass and interactions that suggest the feasibility of exploring vectorial growth strategies from a single fragment (SI Fig. 1b and 2b). For example, fragments located at the upper part of the NCS-1 crevice are particularly promising for modulating the interaction between NCS-1 and D_2_R in a specific manner, as the structural analysis of the protein–protein complexes and protein–PPI modulators suggest (Fig. 1a and c).^6^ This is the case of fragment Z86416893 (SI Fig. 1b). Another interesting fragment that deserves consideration is Z55290386, which binds to the central region of the NCS-1 crevice and establishes three hydrogen bonds with residues Y52 and T92 (SI Fig. 2b), hotspots in the recognition of Ric-8A, D_2_R and CB_1_R. Its pose suggests two plausible growth vectors: (i) towards the upper part of the cavity by inserting hydrophobic groups and (ii) growth toward the lower part of the cavity by inserting polar or hydrophobic groups, thus modulating the dynamics of helix H10 and consequently enabling inhibition or stabilization of the binding partners.^2,12^ Notably, fragment Z55290386 complies with Lipinski's rule, and according to prediction algorithms, is predicted to cross the BBB.

## Discussion

In this work, we used a data-driven, automation-assisted and resource-efficient fragment-to-scaffold progression platform that operates entirely at the synchrotron, delivering structurally validated and direct-to-biology hit series. The platform is inherently generalizable and readily adaptable to diverse biological targets, enabling the exploration of the off-catalogue chemical space of the targets. By combining high-resolution structural analysis, compound design, synthesis prioritization, crude-mixture triage, and orthogonal biophysical validation into a single streamlined environment, this platform constitutes a new framework for testing fragment progression approaches. The complete DMTA cycle was completed in three months: one month was dedicated to the fragment's structural analysis, computational design and compound prioritization; a second month for sourcing and receiving the required building blocks, and the third month for automated synthesis, LC-MS/MSCheck validation, structure solution of follow-up elaborations and biophysical validation. This rapid, data-rich, and experimentally self-contained process lowers the barrier between fragment identification and chemically informed hypothesis generation of hit series for future hit-to-lead development.

Applied to the challenging context of protein–protein interaction modulation, this strategy enabled the maturation of fragment hits against the pharmacologically relevant target NCS-1, generating scaffolds with biological relevance and chemical diversity for further development and optimization (Fig. 8 and 9). This strategy intentionally prioritizes SAR exploration first and property optimization later to deliver higher interaction productivity, chemical diversity and allow an in-depth exploration of the chemical space of the NCS-1 PPI. These scaffolds are distinct from previously reported PPI modulators that have not progressed beyond preclinical stages.^6,9,10,12,13^ Moreover, given the sequence homology and structural conservation within the NCS protein family, the fragments and scaffold series identified here may serve as a foundation for the development of modulators targeting other pharmacologically relevant members of this family.^1,43^

**Fig. 9:**
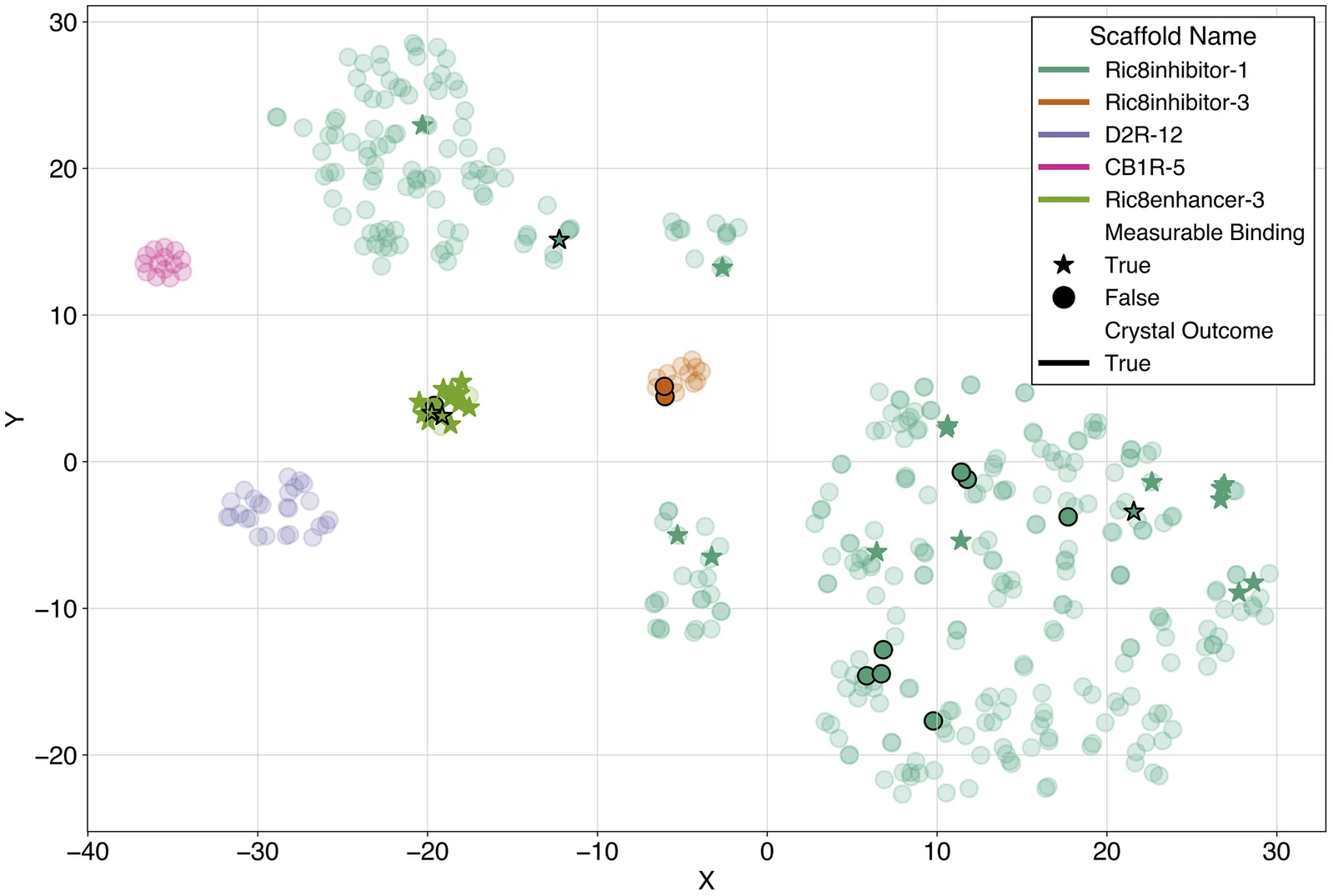
Exploration of the chemical space of the NCS-1 PPI through CAR elaborations. Chemical space of the 454 elaborations synthesized by CAR, where the Morgan fingerprints (2048 bit length, radius = 2) were reduced to two dimensions using the t-SNE algorithm. The color code is assigned to the different populations or clusters based on the common scaffold (base compound) they share: Ric8inhib-1 (teal), Ric8inhib-3 (orange), Ric8enhan-3 (light green), D2R-12 (purple), and CB1R-5 (pink). Compounds with at least one crystallographic structure are represented as dark circles with black outlines, compounds with measurable kinetics are represented as filled stars, and finally compounds with both crystallographic structures and measurable kinetics are represented as stars with black outlines. Compounds with neither data are represented as transparent circles.

The workflow presented here can be positioned against computational fragment follow-up strategies that, like ours, search for fragment analogs and use template-guided docking from crystallographically confirmed fragment poses. The closest catalog-based precedent is Frag4Lead,^19^ in which fragment analogs are retrieved from a vendor catalog and prioritized by template-guided docking for purchase and crystallographic validation. Applied to the rigid protease endothiapepsin, five fragments yielded 28 purchased compounds, ten of which bound (36%) with micromolar affinities. The same template logic has been extended into ultra-large on-demand chemical space by Müller *et al.*,^44^ who template-docked a 2.6-billion-compound REAL Space^45^ from four fragments bound to protein kinase A. They selected 106 compounds of which 93 were synthesized by Enamine. Here, compounds were prioritized by functional assay first, with forty determined active in at least one assay and six of the most promising were confirmed crystallographically, reaching submicromolar affinity. These approaches, together with ours, reflect a successful shared strategy of searching for fragment analogs using template-guided docking.

Our workflow differs from Frag4Lead^19^ and Müller *et al.*^44^ principally in sourcing compounds and throughput. Rather than only purchasing whole products, we purchase reactants and synthesize elaborations in-house by CAR, deliberately prioritizing throughput. For a total procurement cost of €11k, 252 compounds were synthesized and triaged directly from crude mixtures, nearly an order of magnitude more than in the Frag4Lead campaign and double what was tested by Muller *et al.* While our workflow had a lower crystallographic hit rate than Frag4Lead (5% *versus* 36%) and direct affinities could not be measured owing to the unknown concentrations in the crude reaction mixtures, these trade-offs are inherent to prioritizing throughput and lay the groundwork for a methodology that retains this high-throughput direct-to-biology approach while improving hit confirmation and affinity determination. The three-month cycle reported here and the nine-week cycle of Müller *et al.* show that these approaches operate on comparable timescales, and performing synthesis in-house facilitates direct-to-biology learnings for wider application for synchrotron users.

Finally, this work has identified certain limitations that merit consideration and inform future developments:

### Conformational flexibility of the target protein and fragment identification

For systems with significant conformational flexibility, the PanDDA algorithm is only effective if applied correctly. It is the new implementation (PanDDA2, https://github.com/ConorFWild/pandda_2_gemmi) that handles this correctly, whereas the scenario is not supported in the original program.^46^

### The experimental structure of hits *vs.* their parent fragments or the predicted binding poses

Structural divergence has been observed (SI Fig. 11–13). Based on our analysis, one contributing factor relates to how fragments are selected for merging or linking. When multiple binding poses are identified for a given fragment, it would be advisable to consider compound placement against all plausible fragment binding modes rather than a single specific pose. However, how best to integrate and interpret the resulting multiple solutions and how to score them effectively (such as incorporating docking scores) remain open questions. An additional source of the observed structural divergence is specific to this case study and reflects the inherent complexity and conformational plasticity of the large NCS-1 binding crevice. In this context, the conformational plasticity introduced to the compounds when merging or linking fragments deserves consideration.

### Crude reaction mixtures and the binding of hits to the target protein

The off-rate-based biophysical triage enables prioritization across hundreds of compounds, but crude reaction mixtures may contain residual reagents that compete for binding and thereby underestimate the binding response. Accordingly, the reported off-rate values (*k*_off_) from crude extracts should be considered indicative, not definitive.

### Soaking of hits *vs.* fragments

Soaking protocols established for fragments cannot be directly extrapolated to soakings from reaction extracts. In this context, estimation of product conversion is essential to redefine the protocol and determine whether extended soaking times or modified solubilization procedures are required. This would likely translate into the collection of a greater number of crystal structures for the newly developed scaffolds and, consequently, a more comprehensive understanding of how to advance these compounds towards hit-to-lead optimization.

### Structural follow-up hit rates

Improvements will require iterative optimization of the entire fragment progression pipeline rather than individual methods in isolation. Experimental feedback should be used to refine each stage of the workflow, including design (Fragmenstein), compound enumeration (Syndirella), prioritization (HIPPO), and synthesis (CAR), enabling progressive optimization across all intermediate decision-making steps. Notably, despite limited optimization of these individual components, the present workflow already achieved meaningful hit rates and on-scale potency, highlighting the considerable potential of this integrated approach for future synchrotron fragment-based drug discovery campaigns.

## Author contributions

D. M. R.: investigation, data analysis, writing – original draft, reviewing and editing. E. C.: investigation, data analysis and writing. K. K. F., M. W. and M. F.: data analysis and writing. S. P.-S., C. M. R., L. A., A. M. and M. G.: investigation and data analysis. P. G. M. and C. W. E. T: data collection. W. T. and D. F.: conceptualization, investigation, data analysis, writing and reviewing. F.vD.: conceptualization, reviewing, resources, funding acquisition, validation, supervision, and project administration. M. J. S. B.: conceptualization, investigation, data analysis, writing – original draft, reviewing and editing, resources, funding acquisition, validation, supervision, and project administration.

## Conflicts of interest

The authors declare no conflicts of interest.

## Supplementary Material

SC-017-D6SC02031C-s001

## Data Availability

The atomic coordinates and structure factors of NCS-1 in complex with the ligands have been deposited in the Protein Data Bank, https://www.pdb.org/. Structures containing fragment compounds are available under the group deposition ID G_1002352, with accession codes: 7IJ9, 7IKA, 7IKB, 7IKC, 7IKD, 7IKE, 7IKF, 7IKG, 7IKH, 7IKI, 7IKJ, 7IKK, 7IKL, 7IKM, 7IKN, 7IKO, 7IKP, 7IKQ, 7IKR, 7IKS, 7IKT, 7IKU, 7IKV, 7IKW, 7IKX, 7IKY, 7IKZ, 7IK0, 7IK1, 7IK2, 7IK3, 7IK4, 7IK5, 7IK6, 7IK7, 7IK8, 7IK9, 7ILA, 7ILB, 7ILC, 7ILD, 7ILE, 7ILF, 7ILG, 7ILH, 7ILI, 7ILJ, 7ILK, and 7ILL. Accession codes for structures containing fragment-based larger compounds are: 9QK7, 9QK8, 9QK9, 9QKA, 9QKB, 9QKC, 9QKD, 9QKE, 9QKF, 9QKG, 9QKH, 9QKI, 9QKJ, 9QKK, 9QKL and 9QKM. The authors will release the atomic coordinates upon article publication. Raw, processed and curated data files have been deposited on Zenodo https://zenodo.org/records/18876983. Open-source software used in this repository can be found in the public GitHub repositories; Fragmenstein https://github.com/matteoferla/Fragmenstein; Syndirella https://github.com/oxpig/syndirella; HIPPO https://github.com/mwinokan/HIPPO; MSCheck https://github.com/xchem/mscheck; and xchem-CAR https://github.com/Waztom/xchem-CAR. Additionally, the public GitHub repository https://github.com/xchem/NCS1-paper contains the historical version of HIPPO used in this work, as well as selected input and output data to reproduce calculations described in this manuscript. Data supporting this article are included as supplementary information (SI). Supplementary information: a detailed Methods section, SI Fig. S1–S15 and Tables S1–S3. Ref. 47–64 are cited in the SI provided. See DOI: https://doi.org/10.1039/d6sc02031c.

## References

[cit1] Burgoyne R. D., Helassa N., McCue H. V., Haynes L. P. (2019). Calcium Sensors in Neuronal Function and Dysfunction. Cold Spring Harbor Perspect. Biol..

[cit2] Romero-Pozuelo J., Dason J. S., Mansilla A., Banos-Mateos S., Sardina J. L., Chaves-Sanjuan A. (2014). *et al.*, The guanine-exchange factor Ric8a binds to the Ca(2)(+) sensor NCS-1 to regulate synapse number and neurotransmitter release. J. Cell Sci..

[cit3] Kabbani N., Negyessy L., Lin R., Goldman-Rakic P., Levenson R. (2002). Interaction with neuronal calcium sensor NCS-1 mediates desensitization of the D2 dopamine receptor. J. Neurosci..

[cit4] Schlecker C., Boehmerle W., Jeromin A., DeGray B., Varshney A., Sharma Y. (2006). *et al.*, Neuronal calcium sensor-1 enhancement of InsP3 receptor activity is inhibited by therapeutic levels of lithium. J. Clin. Invest..

[cit5] Muñoz-Reyes D., McClelland L. J., Arroyo-Urea S., Sánchez-Yepes S., Sabín J., Pérez-Suárez S. (2023). *et al.* [cited 2024 Aug 19]. The neuronal calcium sensor NCS-1 regulates the phosphorylation state and activity of the Gα chaperone and GEF Ric-8A. eLife.

[cit6] Muñoz-Reyes D., Aguado L., Arroyo-Urea S., Requena C., Pérez-Suárez S., Sánchez-Yepes S. (2025). *et al.*, FDA Drug Repurposing Uncovers Modulators of Dopamine D_2_ Receptor Localization via Disruption of the NCS-1 Interaction. J. Med. Chem..

[cit7] Angelats E., Requesens M., Aguinaga D., Kreutz M. R., Franco R., Navarro G. (2018). Neuronal Calcium and cAMP Cross-Talk Mediated by Cannabinoid CB1 Receptor and EF-Hand Calcium Sensor Interactions. Front. Cell Dev. Biol..

[cit8] Stadel R., Ahn K. H., Kendall D. A. (2011). The cannabinoid type-1 receptor carboxyl-terminus, more than just a tail. J. Neurochem..

[cit9] Mansilla A., Chaves-Sanjuan A., Campillo N. E., Semelidou O., Martinez-Gonzalez L., Infantes L. (2017). *et al.*, Interference of the complex between NCS-1 and Ric8a with phenothiazines regulates synaptic function and is an approach for fragile X syndrome. Proc. Natl. Acad. Sci. U. S. A..

[cit10] Roca C., Martinez-Gonzalez L., Daniel-Mozo M., Sastre J., Infantes L., Mansilla A. (2018). *et al.*, Deciphering the inhibition of the neuronal calcium sensor 1 and the guanine exchange factor Ric8a with a small phenothiazine molecule for the rational generation of therapeutic synapse function regulators. J. Med. Chem..

[cit11] McCue H. V., Haynes L. P., Burgoyne R. D. (2010). The diversity of calcium sensor proteins in the regulation of neuronal function. Cold Spring Harbor Perspect. Biol..

[cit12] Canal-Martin A., Sastre J., Sanchez-Barrena M. J., Canales A., Baldominos S., Pascual N. (2019). *et al.*, Insights into real-time chemical processes in a calcium sensor protein-directed dynamic library. Nat. Commun..

[cit13] Cogram P., Fernández-Beltrán L. C., Casarejos M. J., Sánchez-Yepes S., Rodríguez-Martín E., García-Rubia A. (2022). *et al.*, The inhibition of NCS-1 binding to Ric8a rescues fragile X syndrome mice model phenotypes. Front. Neurosci..

[cit14] Pandalaneni S., Karuppiah V., Saleem M., Haynes L. P., Burgoyne R. D., Mayans O. (2015). *et al.*, Neuronal Calcium Sensor-1 Binds the D2 Dopamine Receptor and G-protein-coupled Receptor Kinase 1 (GRK1) Peptides Using Different Modes of Interactions. J. Biol. Chem..

[cit15] Jumper J., Evans R., Pritzel A., Green T., Figurnov M., Ronneberger O. (2021). *et al.*, Highly accurate protein structure prediction with AlphaFold. Nature.

[cit16] Evans R. , O’Neill M., Pritzel A., Antropova N., Senior A. and Green T., *et al.*, Protein complex prediction with AlphaFold-Multimer, bioRxiv, 2021, preprint, 10.1101/2021.10.04.463034

[cit17] Douangamath A., Powell A., Fearon D., Collins P. M., Talon R., Krojer T. (2021). *et al.*, Achieving Efficient Fragment Screening at XChem Facility at Diamond Light Source. J. Visualized Exp..

[cit18] Fearon D., Powell A., Douangamath A., Dias A., Tomlinson C. W. E., Balcomb B. H. (2025). *et al.*, Accelerating Drug Discovery With High-Throughput Crystallographic Fragment Screening and Structural Enablement. Appl. Res..

[cit19] Metz A., Wollenhaupt J., Glöckner S., Messini N., Huber S., Barthel T. (2021). *et al.*, Frag4Lead: growing crystallographic fragment hits by catalog using fragment-guided template docking. Acta Crystallogr., Sect. D: Struct. Biol..

[cit20] Zhang Y., Zhang Z., Ke D., Pan X., Wang X., Xiao X. (2024). *et al.*, FragGrow: A Web Server for Structure-Based Drug Design by Fragment Growing within Constraints. J. Chem. Inf. Model..

[cit21] Grosjean H., Biggin P. C. (2026). Developments and challenges in hit progression within fragment-based drug discovery. Nat. Commun..

[cit22] Baker L. M., Aimon A., Murray J. B., Surgenor A. E., Matassova N., Roughley S. D. (2020). *et al.*, Rapid optimisation of fragments and hits to lead compounds from screening of crude reaction mixtures. Commun. Chem..

[cit23] Grosjean H., Aimon A., Hassell-Hart S., Thompson W., Koekemoer L., Bennett J. (2025). *et al.*, Binding-Site Purification of Actives (B-SPA) Enables Efficient Large-Scale Progression of Fragment Hits by Combining Multi-Step Array Synthesis With HT Crystallography. Angew. Chem., Int. Ed..

[cit24] Ferla M. P. , Sánchez-García R., Skyner R. E., Gahbauer S., Taylor J. C. and Von Delft F., *et al.*, Fragmenstein: predicting protein-ligand structures of compounds derived from known crystallographic fragment hits using a strict conserved-binding–based methodology [Internet], chemrxiv, 2024, preprint, 10.26434/chemrxiv-2024-17w01, https://chemrxiv.org/engage/chemrxiv/article-details/65d751ab9138d23161b7ea38 PMC11731148 39806443

[cit25] Fieseler K. K. , Winokan M., Morrone J. A., Deane C. M., Von Delft F. and Thompson W.. Syndirella: Synthesis-directed fragment elaboration enables extensive binding site exploration beyond catalog compounds [Internet], chemrxiv, 2025, preprint, 10.26434/chemrxiv-2025-0jd39-v2 PMC13656017 42866252

[cit26] Bourne Y., Dannenberg J., Pollmann V., Marchot P., Pongs O. (2001). Immunocytochemical localization and crystal structure of human frequenin (neuronal calcium sensor 1). J. Biol. Chem..

[cit27] Cox O. B., Krojer T., Collins P., Monteiro O., Talon R., Bradley A. (2016). *et al.*, A poised fragment library enables rapid synthetic expansion yielding the first reported inhibitors of PHIP(2), an atypical bromodomain. Chem. Sci..

[cit28] Downes T. D., Jones S. P., Klein H. F., Wheldon M. C., Atobe M., Bond P. S. (2020). *et al.*, Design and Synthesis of 56 Shape-Diverse 3D Fragments. Chem –Eur. J..

[cit29] Wood D. J., Lopez-Fernandez J. D., Knight L. E., Al-Khawaldeh I., Gai C., Lin S. (2019). *et al.*, FragLites—Minimal, Halogenated Fragments Displaying Pharmacophore Doublets. An Efficient Approach to Druggability Assessment and Hit Generation. J. Med. Chem..

[cit30] O'Reilly M., Cleasby A., Davies T. G., Hall R. J., Ludlow R. F., Murray C. W. (2019). *et al.*, Crystallographic screening using ultra-low-molecular-weight ligands to guide drug design. Drug Discovery Today.

[cit31] Pearce N. M., Bradley A. R., Krojer T., Marsden B. D., Deane C. M., von Delft F. (2017). Partial-occupancy binders identified by the Pan-Dataset Density Analysis method offer new chemical opportunities and reveal cryptic binding sites. Struct. Dyn..

[cit32] Gahbauer S., Correy G. J., Schuller M., Ferla M. P., Doruk Y. U., Rachman M. (2023). *et al.*, Iterative computational design and crystallographic screening identifies potent inhibitors targeting the Nsp3 macrodomain of SARS-CoV-2. Proc. Natl. Acad. Sci. U. S. A..

[cit33] Thompson W. , Waztom/mscheck: Pip install version, Zenodo, 2022, https://zenodo.org/record/7018551, 10.5281/ZENODO.7018551

[cit34] Murray J. B., Roughley S. D., Matassova N., Brough P. A. (2014). Off-Rate Screening (ORS) By Surface Plasmon Resonance. An Efficient Method to Kinetically Sample Hit to Lead Chemical Space from Unpurified Reaction Products. J. Med. Chem..

[cit35] Brough P. A., Baker L., Bedford S., Brown K., Chavda S., Chell V. (2017). *et al.*, Application of Off-Rate Screening in the Identification of Novel Pan-Isoform Inhibitors of Pyruvate Dehydrogenase Kinase. J. Med. Chem..

[cit36] Krissinel E., Henrick K. (2007). Inference of Macromolecular Assemblies from Crystalline State. J. Mol. Biol..

[cit37] Winn M. D., Ballard C. C., Cowtan K. D., Dodson E. J., Emsley P., Evans P. R. (2011). *et al.*, Overview of the CCP4 suite and current developments. Acta Crystallogr., Sect. D: Biol. Crystallogr..

[cit38] Laskowski R. A., Swindells M. B. (2011). LigPlot+: Multiple Ligand–Protein Interaction Diagrams for Drug Discovery. J. Chem. Inf. Model..

[cit39] Bouysset C., Fiorucci S. (2021). ProLIF: a library to encode molecular interactions as fingerprints. J. Cheminf..

[cit40] Alam M. S. , Proximity Ligation Assay (PLA), in Immunohistochemistry and Immunocytochemistry, ed. Del Valle L., Springer US, New York, NY, 2022, pp. 191–201, 10.1007/978-1-0716-1948-3_13

[cit41] González-García E., Miró-Rodríguez C., Ulzurrun E., Toledano Ó., Pérez-Suarez S., Aguado L. (2026). *et al.*, An AI-based approach accelerates the discovery of protein–protein interaction modulators targeting NCS-1. Eur. J. Med. Chem..

[cit42] Wu D., Chen Q., Chen X., Han F., Chen Z., Wang Y. (2023). The blood–brain barrier: structure, regulation, and drug delivery. Signal Transduction Targeted Ther..

[cit43] Bandura J., Feng Z. P. (2019). Current Understanding of the Role of Neuronal Calcium Sensor 1 in Neurological Disorders. Mol. Neurobiol..

[cit44] Müller J., Klein R., Tarkhanova O., Gryniukova A., Borysko P., Merkl S. (2022). *et al.*, Magnet for the Needle in Haystack: “Crystal Structure First” Fragment Hits Unlock Active Chemical Matter Using Targeted Exploration of Vast Chemical Spaces. J. Med. Chem..

[cit45] Enamine , REAL Database, Enamine, available from: https://enamine.net/compound-collections/real-compounds/real-database

[cit46] Pearce N. M., Krojer T., Bradley A. R., Collins P., Nowak R. P., Talon R. (2017). *et al.*, A multi-crystal method for extracting obscured crystallographic states from conventionally uninterpretable electron density. Nat. Commun..

[cit47] Baños-Mateos S., Chaves-Sanjuán A., Mansilla A., Ferrús A., Sánchez-Barrena M. J. (2014). Frq2 from Dsrosophila melanogaster: cloning, expression, purification, crystallization and preliminary X-ray analysis. Acta Crystallogr., Sect. F: Struct. Biol. Commun..

[cit48] Ng J. T., Dekker C., Kroemer M., Osborne M., von Delft F. (2014). Using textons to rank crystallization droplets by the likely presence of crystals. Acta Crystallogr., Sect. D: Biol. Crystallogr..

[cit49] Erlanson D. A., Burley S. K., Fearon D., Fraser J. S., Kreitler D., Nonato M. C. (2025). *et al.*, Where and how to house big data on small fragments. Nat. Commun..

[cit50] Krojer T., Talon R., Pearce N., Collins P., Douangamath A., Brandao-Neto J. (2017). *et al.*, The XChemExplorer graphical workflow tool for routine or large-scale protein–ligand structure determination. Acta Crystallogr., Sect. D: Biol. Crystallogr..

[cit51] Vonrhein C., Flensburg C., Keller P., Sharff A., Smart O., Paciorek W. (2011). *et al.*, Data processing and analysis with the autoPROC toolbox. Acta Crystallogr., Sect. D: Biol. Crystallogr..

[cit52] Winter G., Lobley C. M. C., Prince S. M. (2013). Decision making in xia2. Acta Crystallogr., Sect. D: Biol. Crystallogr..

[cit53] Winter G., Waterman D. G., Parkhurst J. M., Brewster A. S., Gildea R. J., Gerstel M. (2018). *et al.*, DIALS: implementation and evaluation of a new integration package. Acta Crystallogr., Sect. D: Biol. Crystallogr..

[cit54] Wojdyr M., Keegan R., Winter G., Ashton A. (2013). DIMPLE - a pipeline for the rapid generation of difference maps from protein crystals with putatively bound ligands. Acta Crystallogr., Sect. A: Found. Crystallogr..

[cit55] Long F., Nicholls R. A., Emsley P., Gražulis S., Merkys A., Vaitkus A. (2017). *et al.*, AceDRG: a stereochemical description generator for ligands. Acta Crystallogr., Sect. D: Struct. Biol..

[cit56] Emsley P., Cowtan K. (2004). Coot: model-building tools for molecular graphics. Acta Crystallogr., Sect. D: Biol. Crystallogr..

[cit57] Adams P. D., Afonine P. V., Bunkóczi G., Chen V. B., Davis I. W., Echols N. (2010). *et al.*, PHENIX: a comprehensive Python-based system for macromolecular structure solution. Acta Crystallogr., Sect. D: Struct. Biol..

[cit58] Williams C. J., Headd J. J., Moriarty N. W., Prisant M. G., Videau L. L., Deis L. N. (2018). *et al.*, MolProbity:
More and better reference data for improved all-atom structure validation. Protein Sci..

[cit59] Bruno I. J., Cole J. C., Kessler M., Luo J., Motherwell W. D. S., Purkis L. H. (2004). *et al.*, Retrieval of Crystallographically-Derived Molecular Geometry Information. J. Chem. Inf. Comput. Sci..

[cit60] Irwin J. J., Tang K. G., Young J., Dandarchuluun C., Wong B. R., Khurelbaatar M. (2020). *et al.*, ZINC20—A Free Ultralarge-Scale Chemical Database for Ligand Discovery. J. Chem. Inf. Model..

[cit61] Adasme M. F., Linnemann K. L., Bolz S. N., Kaiser F., Salentin S., Haupt V. J. (2021). *et al.*, PLIP 2021: expanding the scope of the protein–ligand interaction profiler to DNA and RNA. Nucleic Acids Res..

[cit62] Grygorenko O. O. (2021). Enamine Ltd.: The Science and Business of Organic Chemistry and Beyond. Eur. J. Org. Chem..

[cit63] Dolinsky T. J., Czodrowski P., Li H., Nielsen J. E., Jensen J. H., Klebe G. (2007). *et al.*, PDB2PQR: expanding and upgrading automated preparation of biomolecular structures for molecular simulations. Nucleic Acids Res..

[cit64] Dolinsky T. J., Nielsen J. E., McCammon J. A., Baker N. A. (2004). PDB2PQR: an automated pipeline for the setup of Poisson-Boltzmann electrostatics calculations. Nucleic Acids Res..

