# Supplementary material for: A resource-efficient structure-based workflow for fragment progression enables parallel hit discovery and validation of functionally diverse modulators of NCS-1 protein–protein interactions
Source: Chem Sci. 2026 Sep 2;17(41):20097–117. doi: 10.1039/d6sc02031c (PMC13575873; doi:10.1039/d6sc02031c)
Supplement: Supplementary file 1 [file SC-017-D6SC02031C-s001.pdf]

Supplementary Information (SI) for Chemical Science.
This journal is © The Royal Society of Chemistry 2026

Electronic Supporting Information

A resource-efficient structure-based workflow for fragment progression enables parallel hit discovery and validation of functionally diverse modulators of NCS-1 protein–protein interactions

Daniel Muñoz-Reyes[1], Kate K. Fieseler[2,3,4], Max Winokan[3,5], Mathew Golding[3,5], Eda Capkin[3,5], Matteo Ferla[2,4], Sara Pérez-Suárez[1], Charlie W. E. Tomlinson[3,5], Peter, G. Marples[3,5], Celia Miró-Rodríguez[1], Lorena Aguado[6], Alicia Mansilla[6,7], Daren Fearon[3,5], Warren Thompson[3,5], Frank von Delft[3,4,5,8]*, Sánchez-Barrena MJ[1]*

[1]Department of Crystallography and Structural Biology. Institute of Physical-Chemistry "Blas Cabrera", CSIC, Serrano 119, Madrid 28006, Spain.
[2]Department of Statistics, University of Oxford, Oxford OX1 3LB, United Kingdom.
[3]Research Complex at Harwell, Harwell Science and Innovation Campus, OX11 0FA, Didcot, United Kingdom.
[4]Centre for Medicines Discovery, University of Oxford, Oxford OX3 7DQ, United Kingdom.
[5]Diamond Light Source Ltd, Harwell Science and Innovation Campus, OX11 0QX, Didcot, United Kingdom.
[6]Department of Neurobiology, Instituto Ramón y Cajal de Investigación Sanitaria, Hospital Universitario Ramón y Cajal, Madrid, Spain.
[7] Department of Systems Biology, Universidad de Alcalá, Madrid, Spain.
[8]Department of Biochemistry, University of Johannesburg, Johannesburg 2006, South Africa.

*To whom correspondence may be addressed. E-mail: xmjose@iqf.csic.es and frank.von-delft@diamond.ac.uk

List of supplementary information

1. Materials and Methods

2. Supplementary figures

Figure S1: Fragments binding to the upper region of the NCS-1 cavity (Sites 1 and 2).
Figure S2: Fragments binding to the middle region of the NCS-1 cavity.
Figure S3: Fragments binding to the lower region of the NCS-1 cavity (Sites 4-6).
Figure S4: Fragments binding to the lower region of the NCS-1 cavity (Sites 7-10).
Figure S5: Predicted interactions of pure compounds.
Figure S6: Base compounds selected as potential modulators of the interaction between NCS-1 and dopamine D_2 or CB_1 receptors for synthetic elaboration via CAR.
Figure S7: Base compounds selected as potential modulators of the interaction between NCS-1 and Ric-8A for synthetic elaboration via CAR.
Figure S8: Overview of the crystallographic structures of the bigger compounds.
Figure S9: Structures of elaborations derived from fragments.
Figure S10: The binding of larger, commercially available compounds to NCS-1 using tryptophan emission fluorescence.

Figure S11: Superimposition of crystallographic structures of Ric8inhib-1 elaborations with poses predicted by Fragmenstein.

Figure S12: Superimposition of crystallographic structures of various elaborations relative to placements predicted by Fragmenstein.

Figure S13: Superimposition of crystallographic structures of various elaborations relative to crystallographic structures of their inspiration fragments.

Figure S14: RMSD of experimentally resolved poses to the predicted pose by compound.

Figure S15: Superimposition of the crystallographic structures of the NCS-1/Ric-8A PPI enhancer 3b and the pure compound E14.

3. Supplementary tables

Table S1: Selected pure catalog compounds.

Table S2: Comparison of affinity results obtained via GCI and Trp fluorescence emission techniques.

Table S3: Summary of the different steps during GCI for NCS-1 capture on the NTA surface.

1. Materials and Methods

Crystallization of NCS-1

Crystals of the full-length wild-type protein were obtained as previously reported (12). The purified protein (12,47) was dialyzed into a solution containing 20 mM sodium acetate pH 5.5, 0.5 mM $CaCl_2$, and 0.5 mM DTT (2 changes, 4 and 16 h), and concentrated to 10 mg/ml (Vivaspin). Crystals grew in a crystallization condition containing 0.1 M sodium cacodylate trihydrate pH 6.5, 0.2 M sodium acetate trihydrate, 30% (v/v) polyethylene glycol 8000 (solution 28, Crystal Screen I, Hampton Research) at 4ºC (12). Crystallization was optimized using an Oryx robot (Douglas Instrument), refining the protein:precipitant ratio, to obtain higher diffraction quality and reproducibility. A final ratio of 200:300 nl produced larger and thicker plates in two days in 90% of the experiments. For fragment screening, crystallization experiments were conducted at the DCyBE (IQF-CSIC) while for lead-like compound screening, crystals were produced at XChem.

High-throughput X-ray fragment screening

Four fragment libraries were selected: DSi-Poised (27), York 3D (28), FragLites (29), and MiniFrags (30). The 1,093 fragments were prepared in 100% DMSO at a concentration of 100 mM, 500 mM or 1 M. Crystallization plates were imaged using a Rock Imager (Formulatrix) at 4°C. The sites for dispensing the fragment-containing solution were selected with TexRank (48), and site coordinates were recorded in SoakDB. Before screening, a DMSO tolerance test was conducted to assess crystal survival and diffraction quality. Incubation times ranged from 1-3 hours with solvent concentrations between 5-30%. After incubation, diffraction data were acquired. The optimal DMSO concentration and soaking time selected were 10% (v/v) and 2 hours at 4ºC. Fragments were dispensed directly to the crystallization drops using the ECHO Liquid Handler (Labcyte), adjusted to a final DMSO concentration of 10% (v/v). Crystals

were incubated for two hours at 4°C, then mounted on nylon loops (MiTeGen) using the Crystal Shifter (Oxford Lab Technologies), and flash-frozen in liquid nitrogen.

Data collection and processing, structural resolution, refinement, and model validation

Diffraction data were collected at 100 K and 0.91507 Å wavelength at the beamline I04-1 (Diamond Light Source, UK) (17,49). Large-scale analysis of protein-fragment complexes was performed with XChemExplorer (XCE) (50). Diffraction data were automatically processed using AutoPROC (51) and XIA2dials (52,53). Initial map calculations were performed with Dimple (54). As search model, the previously solved NCS-1 structure from the same crystal form was used (PDB: 6QI4;(12)) with some modifications. Concretely, the dynamic N- and C-terminal ends were removed, and the search model only contained the region starting at EF-1 and ending at EF-4. A resolution limit of 3.5 Å was set to discard poorly diffracting datasets. Ligand restraint dictionaries were generated using aceDRG (55). The ground-state model, representing an average model of the protein structure without a ligand, was built(31) and used as a reference. An initial hit identification was performed with PanDDA (31). Model building was carried out with Coot (56) and structural refinement with Phenix (57). Structures were validated using MolProbity (58) and Mogul (59). The coordinates and structure factors were deposited in Fragalysis (fragalysis.diamond.ac.uk/) under the project code "NCS1".

Data collection of fragment-based molecules was performed as described above for the fragment-screening campaign. The structure of hNCS-1 (PDB: 6QI4) was used as search model. Given the conformational flexibility of the C-terminal helix H10, this region was omitted from the initial search model and rebuilt manually during model building. Model building and refinement of all structures was performed with Coot (56) and Phenix (57).

Supplementary File 1 and 2 show the statistics related to data processing and refinement for both fragments and fragment-based compounds. The coordinates and structure factors for fragments have been deposited in the Protein Data Bank (PDB) under the group deposition ID G_1002352. The structures of NCS-1 in complex with the fragment-based compounds have been deposited individually in the PDB. All interactions were studied using LigPlot+ (38) and the PISA server (36).

Progression of fragments: Linking and merging of fragments

Fragmenstein (https://github.com/oxpig/Fragmenstein) (32,24) was used to elaborate the fragment-hits in a three-step pipeline: merging/linking, analogue-by-catalogue search, and placement. This algorithm is centered around strict obedience to the 3D position of the parent compounds, based on the premise that analogues with similar structures have a conserved binding mode, making ideal for crystallography-driven fragment-based drug discovery campaign. One fragment binding mode was selected to place each compound even if multiple binding modes were measured. The workflow consisted of: (i) stitching together (link and merge) adjacent fragments or sub-fragments and energy minimizing in place with Fragmenstein via PyRosetta; (ii) searching for through catalogues using NextMove Software SmallWorld hosted at https://sw.docking.org (60) to find commercially available analogs from Enamine, MCule and other suppliers within a graph edit distance of 5 from the merged compound; (iii)

placing the candidate compounds with Fragmenstein and analysing the number of interactions, which are internally determined using the protein−ligand interaction profiler (PLIP) (61). The virtual compounds were subset based on desired and undesirable interactions with key residues, and the ranked compounds filtered by eye. Due to dynamic nature of NCS-1, three templates were used. Additionally, some hypothesis-driven expansions were also enumerated using Next Move Software Arthor, hosted at https://arthor/docking.org (60) and placed with Fragmenstein.

Data dissemination and compound selection using Fragalysis

Fragalysis is a web application developed at XChem to facilitate structural data dissemination, provide GUI-based tools for data curation and annotation, and enable quality assessment by visually comparing the conformational and chemical features of in-silico follow-up designs (displayed on the right-hand-side) with experimental structural data (displayed on the left-hand-side). It also serves as a launch pad for structural biologists, computational chemists and medicinal chemists to collaborate, via shareable web links, with the ultimate endpoint of deciding how project resources should be allocated to progress fragments. In this work, NCS-1 structural data were uploaded to Fragalysis, key fragments were identified and subsequently used as inputs, with the experimental data standardized in Fragalysis, for computational efforts and compound merging via Fragmentstein. Critically, Fragalysis was used by the project team to review and select designs for further elaboration via the Syndirella and HIPPO workflow, for final building block purchase and CAR synthesis.

Design and synthesis of new compounds using CAR

Compound design and selection for synthesis via CAR: Base compounds for further elaboration through digitized chemical synthesis were selected using Syndirella (https://github.com/kate-fie/syndirella). This algorithmic tool produces elaborations from the base compounds that are restricted to specific synthetic routes. First, a retrosynthesis analysis was performed using the tool Manifold (PostEra Inc., https://app.postera.ai/manifold/). Then a catalogue search for superstructures (molecules which contain the query molecule as a maximum common substructure) of each reactant was performed using Manifold. The catalogues selected were Enamine BB, Enamine REAL, Enamine MADE (62), MCule and MCule Ultimate. Elaborated products were produced by performing a cartesian combination of reactants and reacted together *in-silico* with SMARTS patterns. Finally, the elaborations were filtered using Fragmenstein, to discard elaborations that would result in steric clashes with the protein after minimization to the inspiration fragments. Only elaborations using single step synthesis routes were selected. Syndirella elaborations were verified to have <0 kcal/mol $\Delta\Delta G_{bind}$ values and <1 Å RMSDs to the inspiration fragments, showing the selected mergers share a significant three-dimensional space with their inspiration fragments (Table 1). However, the Tanimoto similarity coefficient was relatively low, indicating the introduction of atoms or chemical groups different from those in the inspiration fragments. Despite the low coefficient, the mergers were expected to recapitulate the interactions of the inspiration fragments (Table 1, Supplementary Figure 6 and Supplementary Figure 7).

Classification of compounds for potential PPI modulation of NCS-1 with target proteins: Compounds were classified as potential PPI modulators of NCS-1 PPI with

D_2R, Ric-8A and CB_1R by comparing the overlap between protein-ligand and protein-protein interactions (14,5). In the case of D_2R and Ric-8A, prior knowledge on PPI modulation was considered (9,12,6).

Starting material collections: HIPPO algorithm (Hit Interaction Profiling for Procurement Optimisation) (https://github.com/mwinokan/HIPPO) was used to select the best collection of elaborated products to maximize the use of reagents, synthesizing the highest number of compounds with the minimum number of reagents. Starting material combinations were also considered to increase chemical diversity and explore new interactions. All starting materials were sourced through MCule.

Robotic array synthesis: Synthesis of the compounds was performed using CAR (Chemist Assisted Robotics), a digitized and database driven chemistry platform that facilitates multi-step high-throughput synthesis. The process involved: (i) Reviewing the synthetic routes proposed by Syndirella; (ii) automated generation of Python scripts and starting materials plate preparation for synthesis using an OpenTrons II liquid-handling robot; (iii) the execution of the chemical synthesis using an OpenTrons OT-2 robot. See amidation and sulfonamide reaction as the digitized protocols used in section "Digitised chemistry protocols used". Control reactions validated the generated scripts. Starting materials (building blocks acquired from MCule) were dissolved in 100% DMF at a final concentration of 0.5 M and reactions were designed to yield the final product at a 100 mM concentration. Reactions were carried out in a 384-ECHO-LDV plate at a final volume of 10 µl, within a fume hood at 23°C. For amidation reactions, HATU was used as an activator and DIPEA as the base. Amidation reactions were performed in three different batches. Sulfonamidation reactions (Schotten-Baumann reactions with amines) used DIPEA as the base and were performed in a single batch. The reaction mixtures were sonicated for 12 hours at room temperature with agitation. Post-reaction, the mixtures were concentrated using SPE-Dry and solvent exchanged from DMF to DMSO by drying the ECHO plate for 30 minutes, then adding 10 µl of DMSO to reach a final concentration of 100 mM. 454 reactions were completed over four days.

Digitised chemistry protocols used: The two digitized reaction protocols used for this work are shown below.


```
{"Amidation": {
    "intramolecular": True,
    "recipes": {
      "HATU Amidation": {
        "yield": 85,
        "reactionSMARTS": [
          "[#6:1](=[#8:2])-[#8;H1].[#7;H3,H2,H1:3]>>[#6:1](=[#8:2])-[#7:3]"
        ],
        "references": None,
        "actionsessions": [
          {
            "type": "reaction",
            "driver": "robot",
            "sessionnumber": 1,
            "intermolecular": {
              "actions": [
                {
                  "type": "add",
                  "actionnumber": 1,
                  "content": {
                    "plates": {
                      "fromplatetype": "startingmaterial",
                      "toplatetype": "reaction",
                    },
                    "material": {
                      "SMARTS": "[#6:1](=[#8:2])-[#8;H1]",
                      "SMILES": None,
                      "quantity": {"value": 1.0, "unit": "moleq"},
                      "solvent": "DMF",
                      "concentration": 0.5,
                    },
                  },
                },
                {
                  "type": "add",
                  "actionnumber": 2,
                  "content": {
```

```
                    "plates": {
                        "fromplatetype": "startingmaterial",
                        "toplatetype": "reaction",
                    },
                    "material": {
                        "SMARTS": None,
                        "SMILES": "CN(C)C(=[N+](C)C)N1C2=C(N=CC=C2)[N+](=N1)[O-].F[P-](F)(F)(F)(F)F",
                        "quantity": {"value": 1.1, "unit": "moleq"},
                        "solvent": "DMF",
                        "concentration": 0.5,
                    },
                },
            },
            {
                "type": "add",
                "actionnumber": 3,
                "content": {
                    "plates": {
                        "fromplatetype": "startingmaterial",
                        "toplatetype": "reaction",
                    },
                    "material": {
                        "SMARTS": None,
                        "SMILES": "CCN(C(C)C)C(C)C",
                        "quantity": {"value": 3.0, "unit": "moleq"},
                        "solvent": "DMF",
                        "concentration": 10,
                    },
                },
            },
        ],
    },
    "Intramolecular": {
        "actions": [
            {
                "type": "add",
                "actionnumber": 4,
                "content": {
                    "plates": {
                        "fromplatetype": "startingmaterial",
                        "toplatetype": "reaction",
                    },
                    "material": {
                        "SMARTS": "[#6:1](=[#8:2])-[#8;H1]",
                        "SMILES": None,
                        "quantity": {"value": 1.0, "unit": "moleq"},
                        "solvent": "DMF",
                        "concentration": 0.5,
                    },
                },
            },
            {
                "type": "add",
                "actionnumber": 5,
                "content": {
                    "plates": {
                        "fromplatetype": "startingmaterial",
                        "toplatetype": "reaction",
                    },
                    "material": {
                        "SMARTS": None,
                        "SMILES": "CN(C)C(=[N+](C)C)N1C2=C(N=CC=C2)[N+](=N1)[O-].F[P-](F)(F)(F)(F)F",
                        "quantity": {"value": 1.1, "unit": "moleq"},
                        "solvent": "DMF",
                        "concentration": 0.5,
                    },
                },
            },
            {
                "type": "add",
                "actionnumber": 6,
                "content": {
                    "plates": {
                        "fromplatetype": "startingmaterial",
                        "toplatetype": "reaction",
                    },
                    "material": {
                        "SMARTS": None,
                        "SMILES": "CCN(C(C)C)C(C)C",
                        "quantity": {"value": 3.0, "unit": "moleq"},
                        "solvent": "DMF",
                        "concentration": 10,
                    },
                },
            },
        ],
    },
},
{
    "type": "reaction",
    "driver": "robot",
    "sessionnumber": 2,
    "continuation": True,
    "intermolecular": {
        "actions": [
            {
                "type": "add",
                "actionnumber": 7,
                "content": {
                    "plates": {
                        "fromplatetype": "startingmaterial",
                        "toplatetype": "reaction",
                    },
                    "material": {
                        "SMARTS": "[#7;H3,H2,H1]",
                        "SMILES": None,
                        "quantity": {"value": 1.0, "unit": "moleq"},
                        "solvent": "DMF",
                        "concentration": 0.5,
                    },
```

```
                    },
                },
            ],
        },
    },
    {
        "type": "stir",
        "driver": "human",
        "sessionnumber": 3,
        "actions": [
            {
                "type": "stir",
                "actionnumber": 8,
                "content": {
                    "platetype": "reaction",
                    "temperature": {"value": 25, "unit": "degC"},
                    "duration": {"value": 12, "unit": "hours"},
                },
            },
        ],
    },
    ],
  },
 },
},
"Sulfonamide Schotten-Baumann with amine": {
    "intramolecular": True,
    "recipes": {
        "DMF": {
            "yield": 75,
            "reactionSMARTS": [
                "[#16:5](=[#8])(=[#8:7])-[#17].[#7;H1,H2:2]>>[#16:5](=[#8])(=[#8:7])-[#7:2]"
            ],
            "references": None,
            "actionsessions": [
                {
                    "type": "reaction",
                    "driver": "robot",
                    "sessionnumber": 1,
                    "intermolecular": {
                        "actions": [
                            {
                                "type": "add",
                                "actionnumber": 1,
                                "content": {
                                    "plates": {
                                        "fromplatetype": "startingmaterial",
                                        "toplatetype": "reaction",
                                    },
                                    "material": {
                                        "SMARTS": "[#16:5](=[#8])(=[#8:7])-[#17]",
                                        "SMILES": None,
                                        "quantity": {"value": 1.0, "unit": "moleq"},
                                        "solvent": "DMF",
                                        "concentration": 0.5,
                                    },
                                },
                            },
                            {
                                "type": "add",
                                "actionnumber": 2,
                                "content": {
                                    "plates": {
                                        "fromplatetype": "startingmaterial",
                                        "toplatetype": "reaction",
                                    },
                                    "material": {
                                        "SMARTS": "[#6]-[#7;H2,H1:2]",
                                        "SMILES": None,
                                        "quantity": {"value": 1.0, "unit": "moleq"},
                                        "solvent": "DMF",
                                        "concentration": 0.5,
                                    },
                                },
                            },
                            {
                                "type": "add",
                                "actionnumber": 3,
                                "content": {
                                    "plates": {
                                        "fromplatetype": "startingmaterial",
                                        "toplatetype": "reaction",
                                    },
                                    "material": {
                                        "SMARTS": None,
                                        "SMILES": "CCN(C(C)C)C(C)C",
                                        "quantity": {"value": 2.4, "unit": "moleq"},
                                        "solvent": None,
                                        "concentration": None,
                                        "density": 0.74,
                                    },
                                },
                            },
                        ],
                    },
                    "intramolecular": {
                        "actions": [
                            {
                                "type": "add",
                                "actionnumber": 4,
                                "content": {
                                    "plates": {
                                        "fromplatetype": "startingmaterial",
                                        "toplatetype": "reaction",
                                    },
                                    "material": {
                                        "SMARTS": "[#16:5](=[#8])(=[#8:7])-[#17]",
                                        "SMILES": None,
                                        "quantity": {"value": 1.0, "unit": "moleq"},
                                        "solvent": "DMF",
```

```
                    "concentration": 0.5,
                  },
                },
              },
              {
                "type": "add",
                "actionnumber": 5,
                "content": {
                  "plates": {
                    "fromplatetype": "startingmaterial",
                    "toplatetype": "reaction",
                  },
                  "material": {
                    "SMARTS": None,
                    "SMILES": "CCN(C(C)C)C(C)C",
                    "quantity": {"value": 2.4, "unit": "moleq"},
                    "solvent": None,
                    "concentration": None,
                    "density": 0.74,
                  },
                },
              },
            ]
          },
        },
        {
          "type": "stir",
          "driver": "human",
          "sessionnumber": 2,
          "actions": [
            {
              "type": "stir",
              "actionnumber": 6,
              "content": {
                "platetype": "reaction",
                "temperature": {"value": 25, "unit": "degC"},
                "duration": {"value": 12, "unit": "hours"},
              },
            },
          ],
        },
      ],
    },
  },
},
}
```


Product analysis via LC-MS: 0.5 µl of each reaction mixture was dissolved in 100 µl of acetonitrile. LC-MS data were collected using an Agilent 1260 HPLC system coupled with an electrospray ionization mass spectrometer G6120 LC-MS Single Quad and a Supelco Ascentis C18 column. The mobile phase consisted of 0.1% formic acid in water (Phase A) and acetonitrile (Phase B). Synthesized products were identified using MSCheck (33), a semi-automated LC-MS data analysis tool that detects $M+H^+$ and $M+Na^+$ ion pairs from chromatogram peaks, where M is the molecular mass of the synthesis product. A manual identification of smaller synthesis peaks was also conducted to detect lower conversion rates. The final products were not purified.

SAR by catalog

In addition to CAR synthesis, compounds from commercial catalogs were selected to expand the chemical diversity of mergers obtained through Fragmenstein. These compounds were purchased from Enamine (Supplementary Table 1).

High-throughput screening of crude reaction mixtures and pure compounds via X-ray crystallography

As with the fragments, incubations and diffraction data collection were performed on synthesized compounds using crude reaction mixtures from CAR and pure available compounds. An initial round of screening involved all synthesized compounds with 2-hour incubations. A second round focused only on synthesis products detected via LC-MS, using incubation times of 8 and 18 hours per reaction mixture.

Screening of compounds in solution via grating-coupled interferometry (GCI)

The Creoptix WAVEdelta system (Creoptix AG) equipment was used to measure binding affinities. Data analysis was carried out using software integrated with the equipment, which performed corrections based on DMSO calibration curves, using blank injections (FC1) as a reference. For pure compounds, kinetic parameters R_{max}, k_{on}, k_{off}, and K_d were automatically calculated by the software through fitting to a 1:1 equilibrium model, using the RAPID assay, which depends on the ligand concentration. For reaction mixtures, an adjustment was made based on the dissociation rate constant (k_{off}), as the exact concentration of products in the reaction mixtures is unknown (22). Evaluation criteria include a R_{max} limit (15 pg/mm^2) based on the control E17 compound, the binding profile of samples on both active and blank channels and checking the non-specific binding to the blank channel. After the fitting, the dissociation curves and the k_d values were reviewed to exclude the false positive hits. The off-rate screening assay was performed in duplicate, and the binders were chosen based on the binding response and k_d values in both runs. The control E17 sample was repeatedly included in the assay to monitor the NCS-1 activity and binding response over the run.

Interaction overlap analysis

Apo structures were protonated with PDB2PQR (63,64) (PROPKA at pH 7.0, AMBER forcefield) to add explicit hydrogens on the protein for reliable hydrogen-bond detection. The resulting PQR files were converted back to PDB before analysis. Protein–ligand interaction fingerprints were computed with ProLIF (39) (Protein–Ligand Interaction Fingerprints) on backbone-aligned holo structures, using protein and ligand from the same PDB. ProLIF's default interaction types (van der Waals, hydrogen bonding, hydrophobic, and π-stacking) were used with a 6.0 Å residue vicinity cutoff. Fingerprints were generated for fragment crystal structures, predicted poses, and experimental crystal structures for each compound. Pairwise overlap between interaction sets (fragment vs predicted, predicted vs experimental, fragment vs experimental) was used to quantify agreement across structure types.

Tryptophan emission fluorescence

NCS-1 emits intrinsic fluorescence when excited at 295 nm, due to the presence of Trp and Tyr residues located at the hydrophobic crevice. The binding of compounds to the crevice changes the 3D environment of these residues and allows the estimation of an apparent affinity constant (9). The fluorescence intensity was measured at 330 nm in a nano-DSF apparatus (nanoTemper) at increasing concentrations of the pure compounds at 35ºC. Protein concentration was set to 5 µM and ligands, from 25 µM to 250 µM in a final buffer containing 50 mM Tris pH 8.0, 125 mM NaCl, 5 µM $CaCl_2$ and 5% DMSO. Three independent experiments were performed with each compound. The apparent dissociation constant, K_d was calculated using a least squares algorithm to fit the recorded data to the equation previously described (9).

Analysis of interactions between NCS-1 and fragment-based molecules

Interactions between NCS-1 and fragment-based molecules were analyzed using NCONT from the CCP4 package with a 4.2 Å distance cutoff (37), LigPlot+ (38) and the PISA server (36). The number of contacts and interacting residues reported in Table 2 were obtained from the NCONT analysis and inspected with LigPlot+. Buried surface area, BSA, and the estimated solvation free-energy gain upon complex formation, ΔG,

reported in Table 2 were calculated using the PISA server. The ΔG values are computational estimates derived from the crystal structures and do not correspond to experimentally measured binding free energies.

Protein-protein interaction binding assays

Co-immunoprecipitations: hNCS-1 and V5-tagged hRic-8A constructs were co-transfected into HEK293 (ATCC) using Lipofectamine 2000 (Invitrogen). 24 h after transfection, DMSO alone or compound E14 dissolved in DMSO (20 μM) were added to the culture cells. Then, 24 h after cells were lysed in buffer 150 mM NaCl, 1.0% Nonidet P-40, 50 mM Tris pH 8.0 in the presence of the compounds (20 μM), whose concentration was maintained throughout the immunoprecipitation assay. Lysates were first incubated for 12 h at 4°C with mouse anti-NCS-1 (1:500; Cell Signaling) and next, for 2 h with Protein-G-Sepharose (Sigma-Aldrich). Samples were washed and eluted from the Sepharose for analysis by Western blot, following standard procedures. 10% of the lysate before immunoprecipitation was run as input. For Western blot, mouse anti-V5 (1:5000; Thermo) and rabbit anti-NCS-1 (1:2000; Cell Signaling) antibodies were used. The immunoprecipitation blot was incubated with anti-mouse TrueBlot (Rockland) as secondary antibody to avoid heavy-/light-chain antibody interference. Input blots were incubated with anti-mouse or anti-rabbit HRP-conjugated antibodies (Sigma 1:5000). HRP activity from the secondary antibodies was revealed with ECL (Promega) and pictures were taken with Chemidoc (Bio-Rad).

Proximity ligation assay: To better quantify the interaction, a Proximity Ligation Assay (PLA) followed by flow cytometry was performed. Cells treated in the same conditions as for the co-IP were detached and dissociated with trypsin. Cells were then fixed for 20 minutes with Fixation reagent and were permeabilized with Permeabilization reagent, all from the Intracellular Fixation and Permeabilization buffer set (eBioscience, Thermo). This set allows a mild fixation and adequate permeabilization, which is indicated to carry out flow cytometry experiments. A mixture of the primary antibodies: Mouse anti-V5 (1:2,000; Thermo) and rabbit anti–NCS-1 (1:1,000; Cell Signaling) diluted in the permeabilization reagent, was used as a first step. Secondly, cells were incubated with the Duolink PLA anti-rabbit MINUS and anti-mouse PLUS probes (Sigma), negative controls without primary antibodies are included in the experiment. Finally, interactions were detected with Duolink PLA Flow Citometry Green Detection Kit (Sigma) following manufacturer instructions. Cells were analyzed in FACS Canto II (BD Bioscience) with BD FACS Diva software.

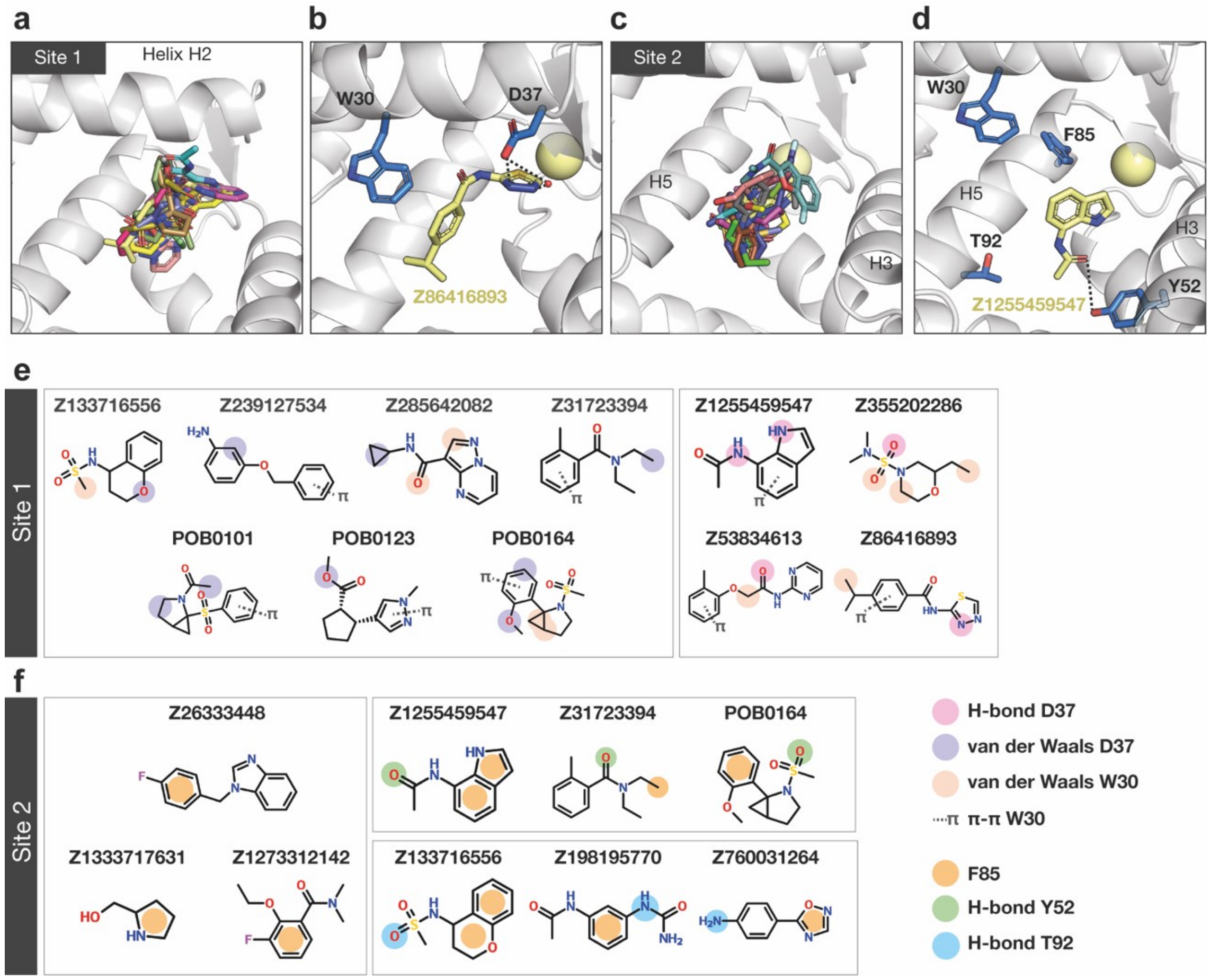

Figure S1: Fragments binding to the upper region of the NCS-1 cavity (Sites 1 and 2). (**a**) Superposition of fragments interacting with helix H2 (Site 1). NCS-1 is shown in ribbon form, and the fragments are displayed as sticks in different colors. (**b**) Representative figure of the interactions of the fragments at Site 1. Only key residues, W30 and D37, are shown as blue sticks, while the fragment Z86416893 is displayed as yellow sticks. (**c**) Superposition of fragments interacting with F85 and residues from helices H3 and H5 (Site 2). (**d**) Representative figure of the interactions of fragments binding at Site 2. Key interacting residues W30, Y52, F85, and T92 are displayed. (**e**) and (**f**) 2D structures of unique fragments and their interactions with NCS-1 at Site 1 (E) and Site 2 (F). Hydrogen bonds with D37 (pink), van der Waals interactions with D37 (light purple), van der Waals interactions with W30 (light orange), and π-π interactions with W30 are indicated. Additionally, hydrophobic and π-π interactions with F85 (orange), hydrogen bonds with Y52 (green), and hydrogen bonds with T92 (light blue) are represented.

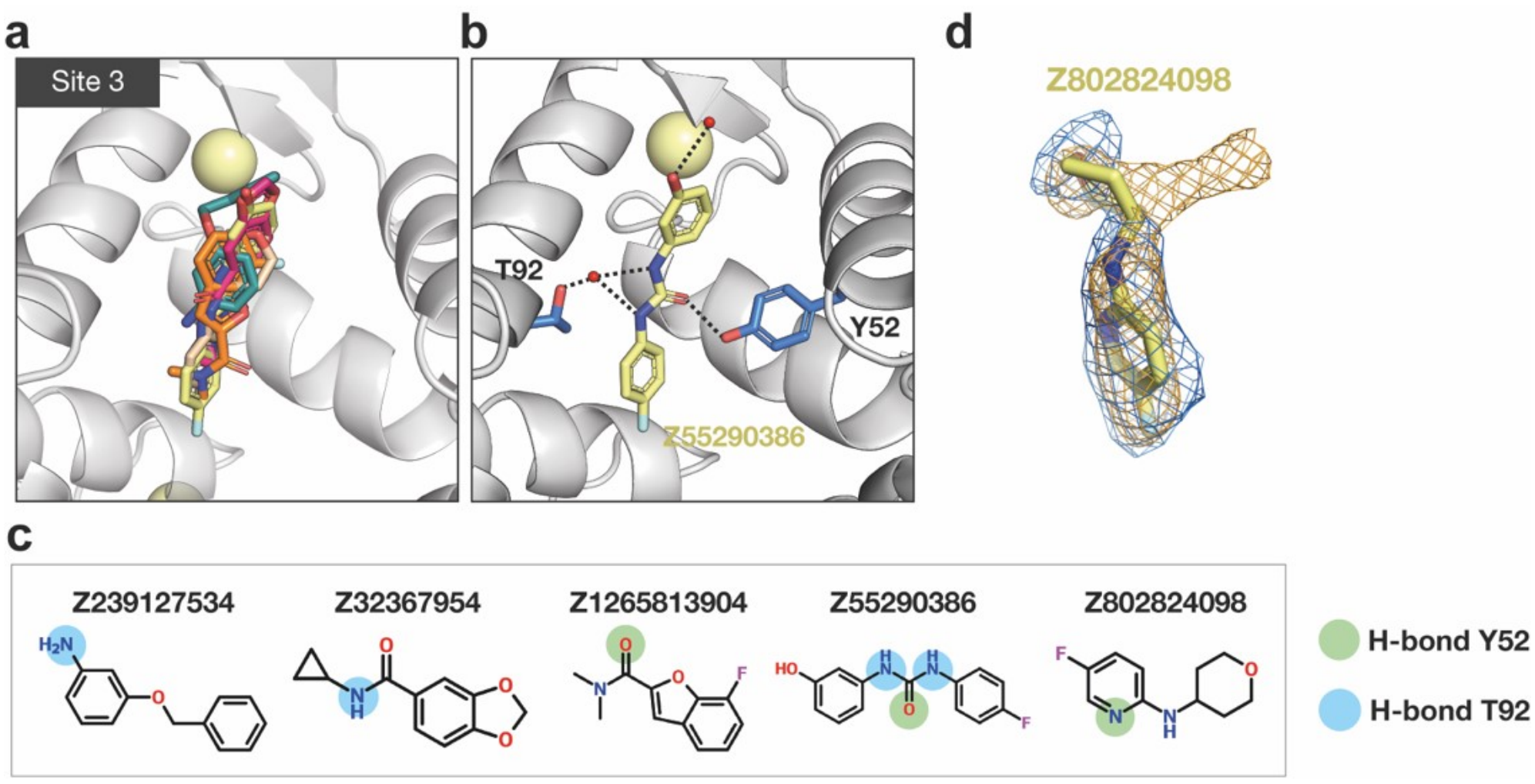

Figure S2: Fragments binding to the middle region of the NCS-1 cavity. (**a**) Superposition of fragments located at Site 3. (**b**) Representative figure of the interactions occurring at Site 3. The fragment Z55290386 forms hydrogen bonds with Y42 and T92. (**c**) 2D structures of unique fragments at Site 3. Hydrogen bonds with Y52 (green) and T92 (blue) are represented. (**d**) Despite some ambiguity in the electron density around the tetrahydropyran ring of fragment Z802824098, the binding pose of the pyridine ring provided valuable insight for follow-up design. The PanDDA event map (orange) suggested two possible conformations for the tetrahydropyran ring, both of which were initially modeled and refined. However, the 2Fo–Fc map (blue) indicated that one conformation was predominant, leading to the removal of the low-occupancy alternative.

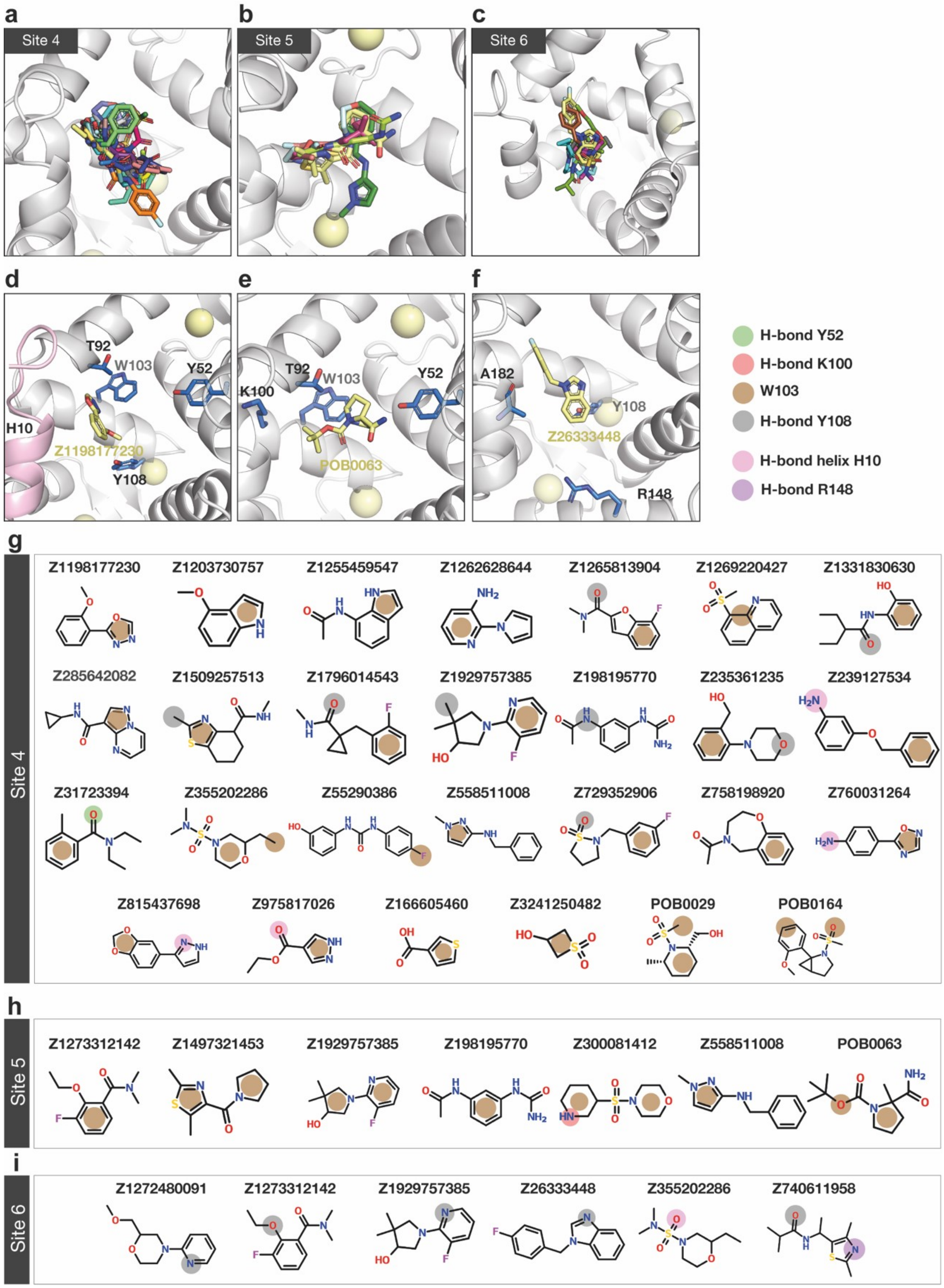

Figure S3: Fragments binding to the lower region of the NCS-1 cavity (Sites 4-6). (**a**), (**b**), (**c**) Superposition of fragments binding at Sites 4, 5, and 6, respectively. (**d**), (**e**), (**f**) Representative figures of the interactions occurring at Sites 4, 5, and 6, respectively. Fragments Z1198177230 (Site 4), POB0063 (Site 5), and Z26333448 (Site 6) are displayed as yellow sticks. The relevant NCS-1 side chains involved in recognition are shown as blue sticks. (**g**), (**h**), (**i**) 2D structures of the fragments and interactions with NCS-1. Hydrogen bonds with Y52 (green), K100 (red), Y108 (gray), residues from helix

H10 (pink), and R148 (purple) are represented. Additionally, van der Waals contacts with W103 (brown) are shown.

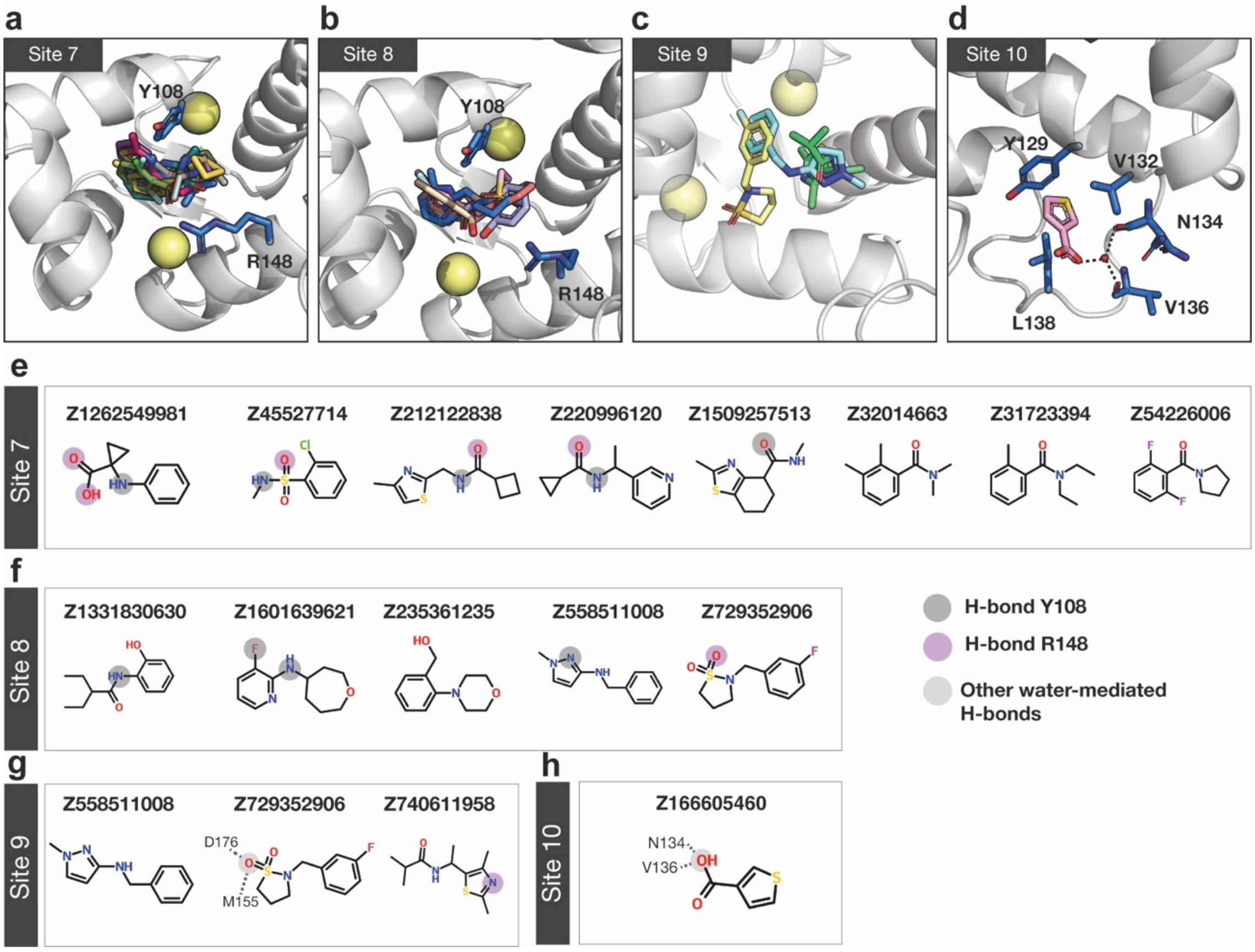

Figure S4: Fragments binding to the lower region of the NCS-1 cavity (Sites 7-10). (**a**), (**b**), (**c**), (**d**) Superposition of fragments binding at Sites 7, 8, 9, and 10, respectively. (**e**), (**f**), (**g**), (**h**) 2D structures of the unique fragments. Only hydrogen bonds with Y108 (gray), R148 (purple), and other bonds mediated by water molecules (light gray) are represented.

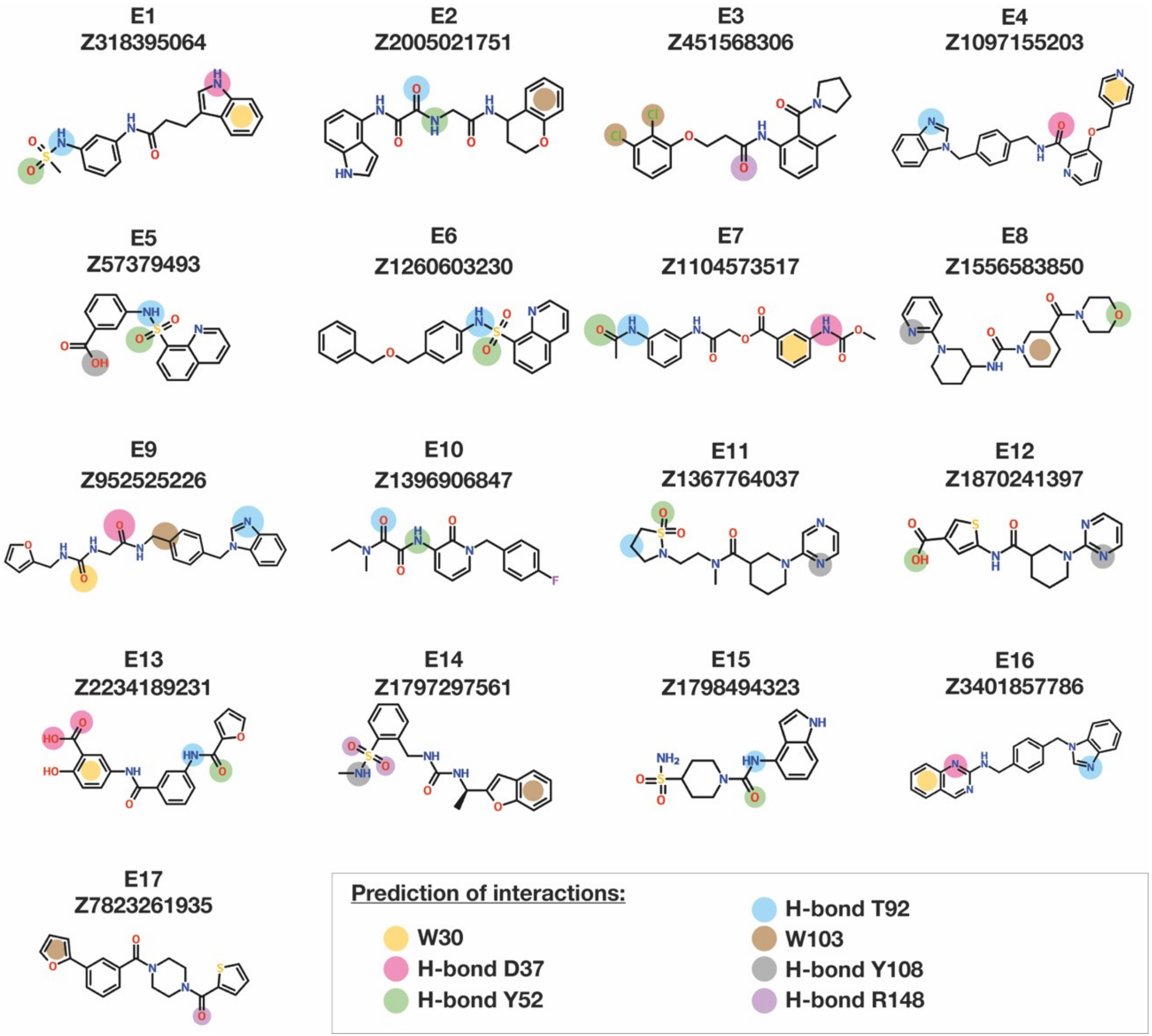

Figure S5: Predicted interactions of pure compounds. The 2D structure of each compound is shown, along with the assigned code (E1-E17) and the commercial code from Enamine. The predicted interactions are represented by circles of different colors.

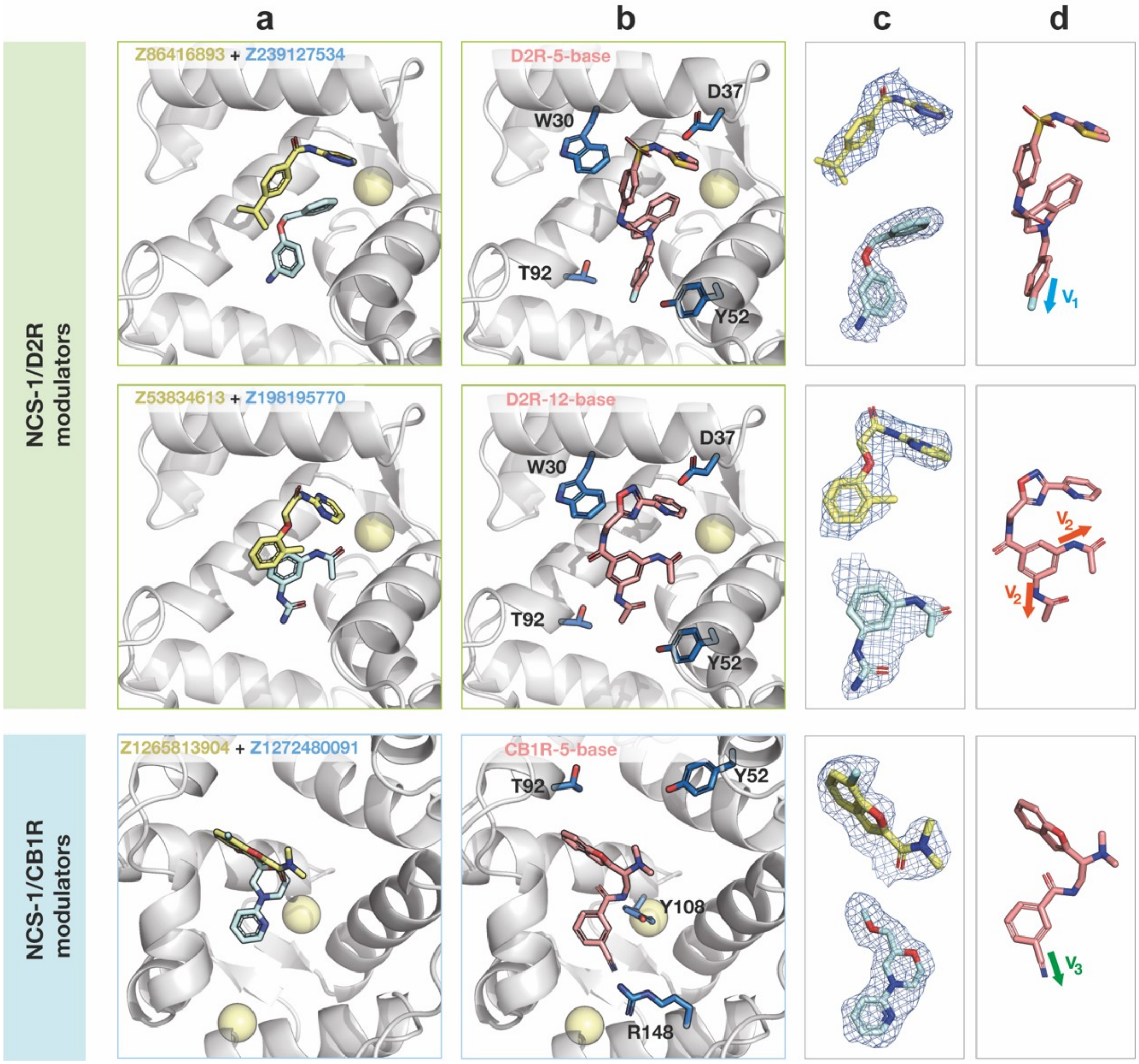

Figure S6: Base compounds selected as potential modulators of the interaction between NCS-1 and dopamine D_2 or CB_1 receptors for synthetic elaboration via CAR. (**a**) The crystallographic structure of the fragments (blue and yellow) that inspired the creation of the merged compounds is shown. (**b**) Representation of the placement using Fragmenstein. The key NCS-1 residues (in dark blue) and the theoretical three-dimensional structure of the mergers (in salmon color) are shown. (**c**) Electron density map 2Fo-Fc of the inspiration fragments (contour at 1.0 σ). (**d**) Vectors (Vx) detected in the theoretical structures of the base compounds for molecules elaborations.

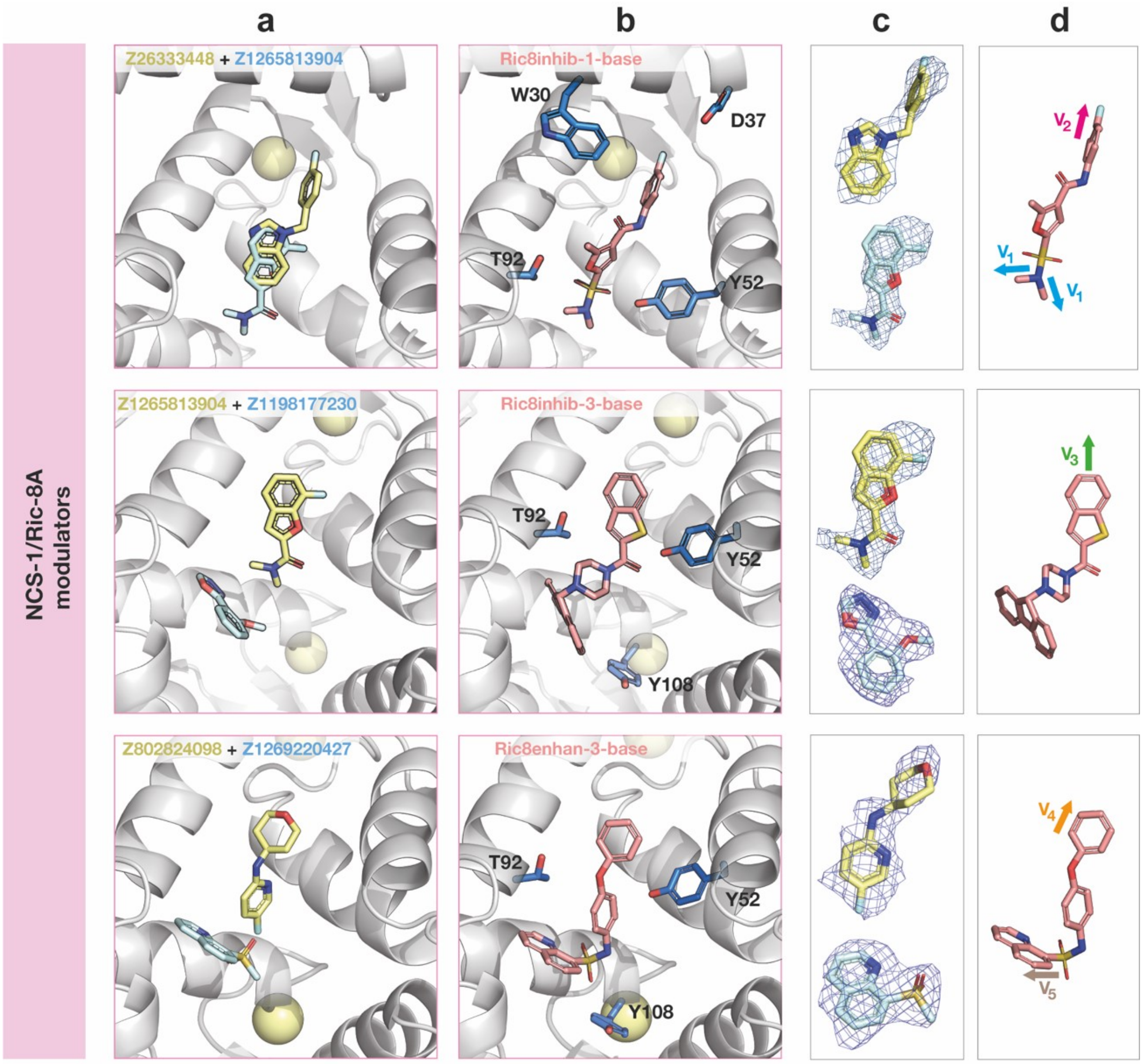

Figure S7: Base compounds selected as potential modulators of the interaction between NCS-1 and Ric-8A for synthetic elaboration via CAR. (**a**) Crystallographic structure of the inspiration fragments. Fragments are shown as yellow and blue sticks. (**b**) Placement representation using Fragmenstein in the NCS-1 crevice (theoretical structures). The key interaction residues and the theoretical three-dimensional structure of the merger (salmon) are shown. (**c**) Electron density map 2Fo-Fc of the inspiration fragments (contour at 1.0 σ) in dark blue. (**d**) Visual vectors detected in the theoretical structures of the base compounds for progression via elaboration.

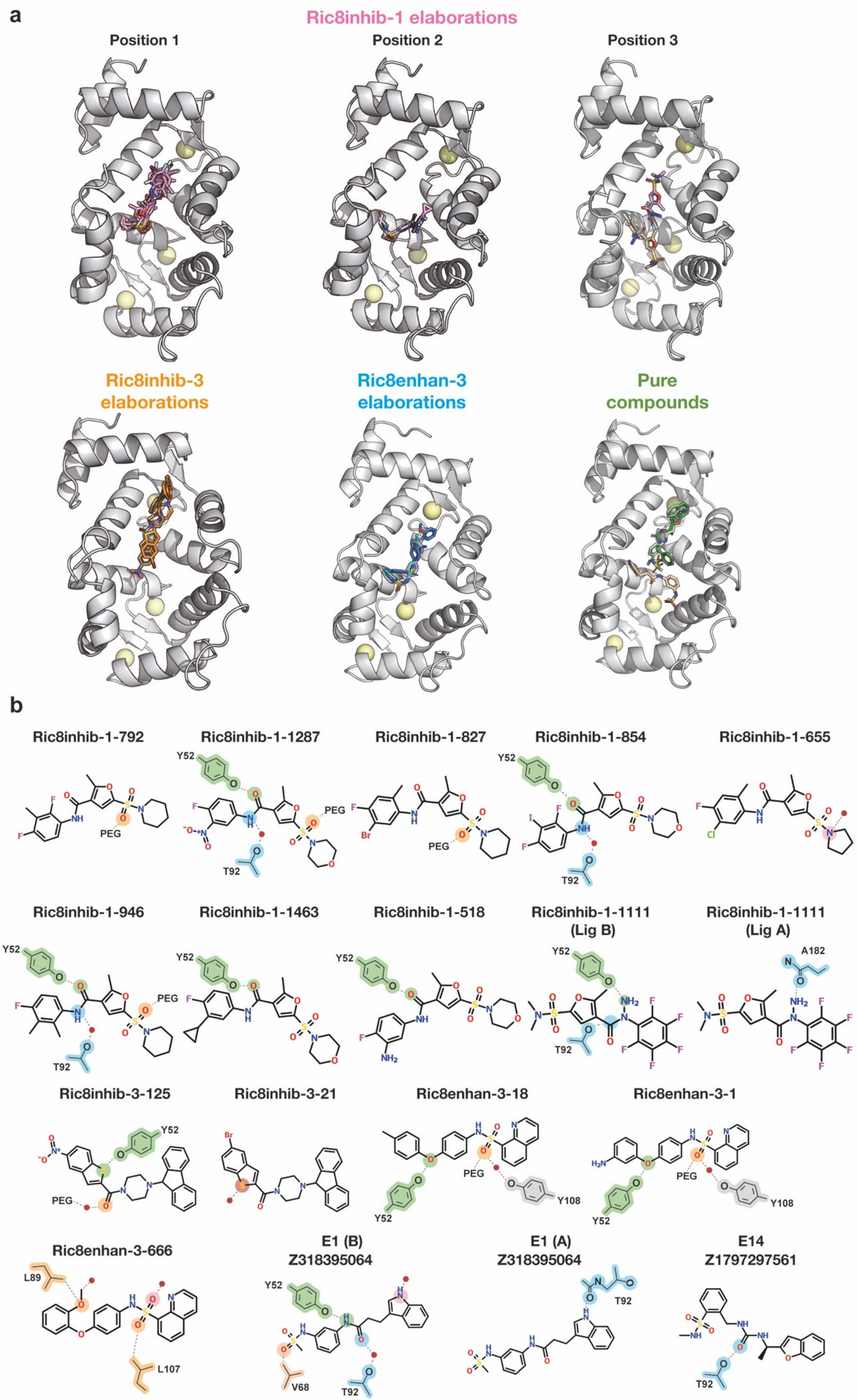
a
Ric8inhib-1 elaborations
Position 1
Position 2
Position 3
Ric8inhib-3 elaborations
Ric8enhan-3 elaborations
Pure compounds
b
Ric8inhib-1-792
Ric8inhib-1-1287
Ric8inhib-1-827
Ric8inhib-1-854
Ric8inhib-1-655
Ric8inhib-1-946
Ric8inhib-1-1463
Ric8inhib-1-518
Ric8inhib-1-1111 (Lig B)
Ric8inhib-1-1111 (Lig A)
Ric8inhib-3-125
Ric8inhib-3-21
Ric8enhan-3-18
Ric8enhan-3-1
Ric8enhan-3-666
E1 (B) Z318395064
E1 (A) Z318395064
E14 Z1797297561
Y52
T92
PEG
A182
Y108
L89
L107
V68

Figure S8: Overview of the crystallographic structures of the bigger compounds. (**a**) Superposition of the different crystallized elaborations related to base compounds Ric8inhib-1 (pink), which have been classified in three different positions (Position 1, 2, and 3), Ric8inhib-3 (orange), Ric8enhan-3 (blue) and pure Enamine compounds (green). Ca^{2+} ions are depicted as yellow spheres. Ric8inhib-1-1111 and E1 show two binding events. Those found in NCS-1 chain A (Lig A) are depicted in wheat color. (**b**) 2D structure of the crystallized elaborations showing H-bond interactions (dashed line) with NCS-1.

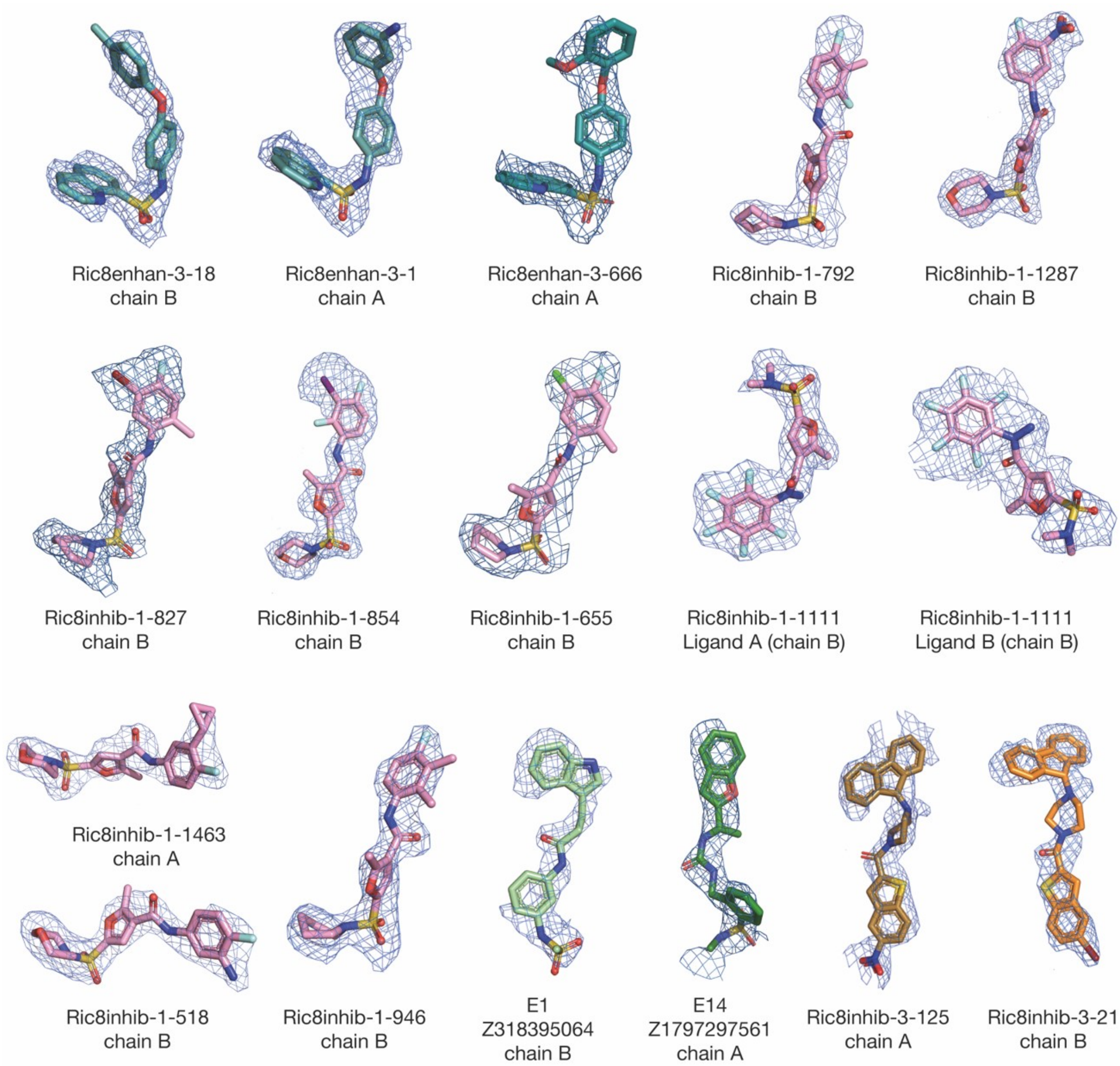

Figure S9: Structures of elaborations derived from fragments. The 2Fo-Fc electron density map is shown in dark blue, contoured at 1σ. The Ric8inhib-3-125 map is contoured at 0.8σ. The protein chain where the ligand is located is indicated (chain A or B). 2 molecules of Ric8inhib-1-1111 bind to the NCS-1 crevice, as specified.

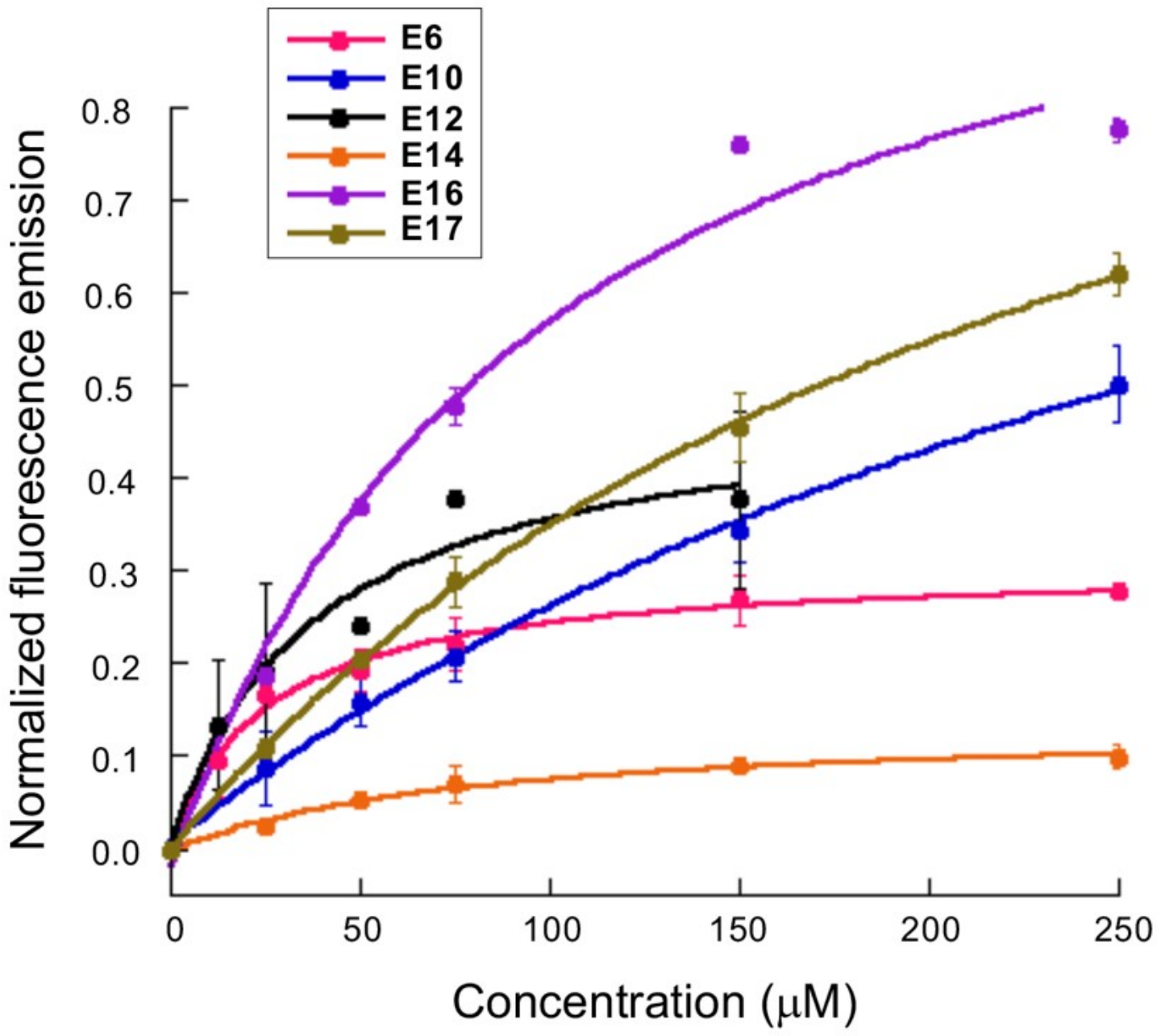

Figure S10: The binding of larger, commercially available compounds to NCS-1 using tryptophan emission fluorescence. Normalized intrinsic fluorescence emission of NCS-1 (mean + SEM, n=3) at increasing concentrations of the pure compounds. Curves correspond to the least squares fitting of the recorded data to a 1:1 stoichiometry equilibrium.

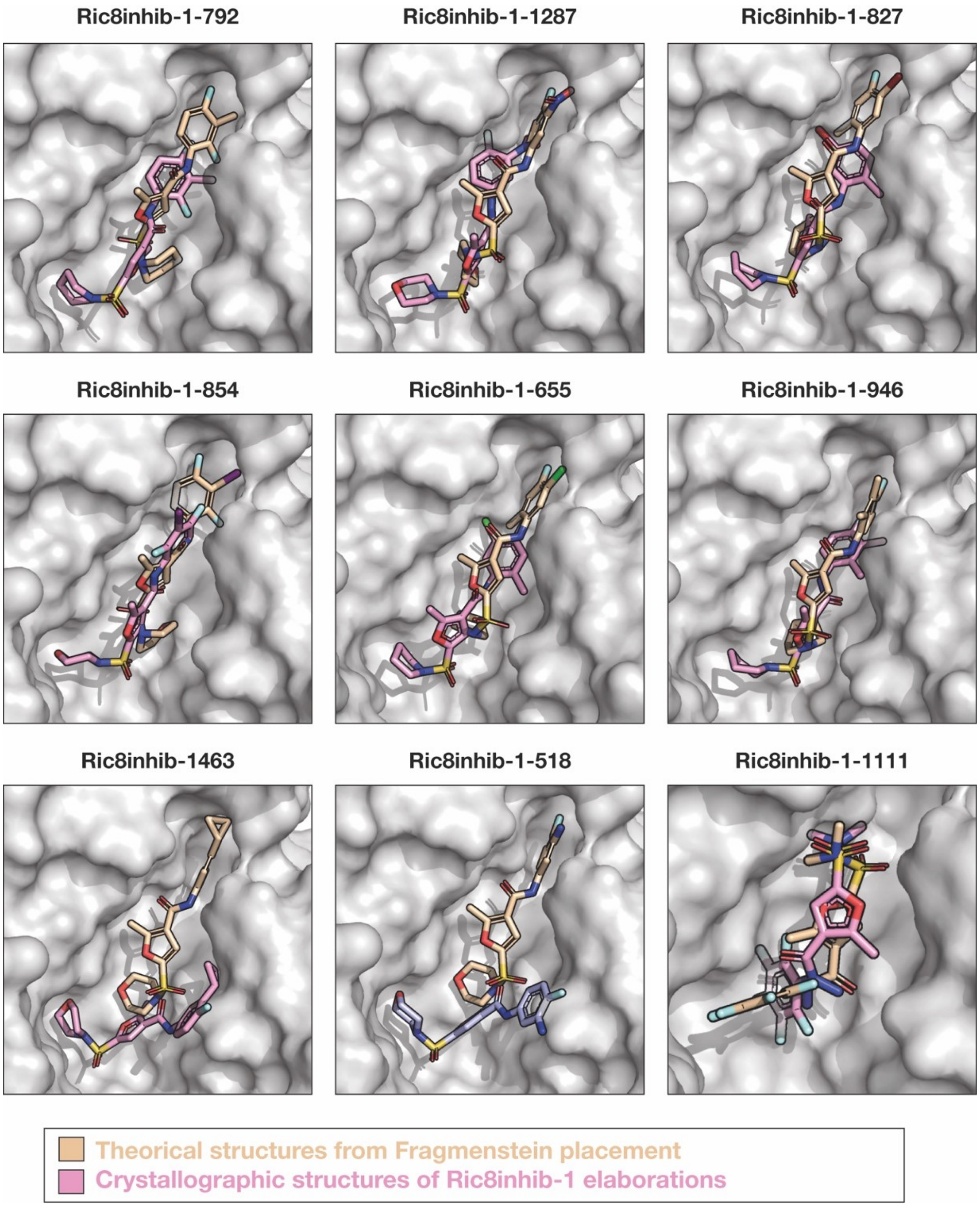

Figure S11. Superimposition of crystallographic structures of Ric8inhib-1 elaborations with poses predicted by Fragmenstein. The proposed placements by Fragmenstein, based on fragment structures, are shown as light orange sticks. The crystallographic structures of Ric8inhib-1 elaborations are displayed as pink sticks.

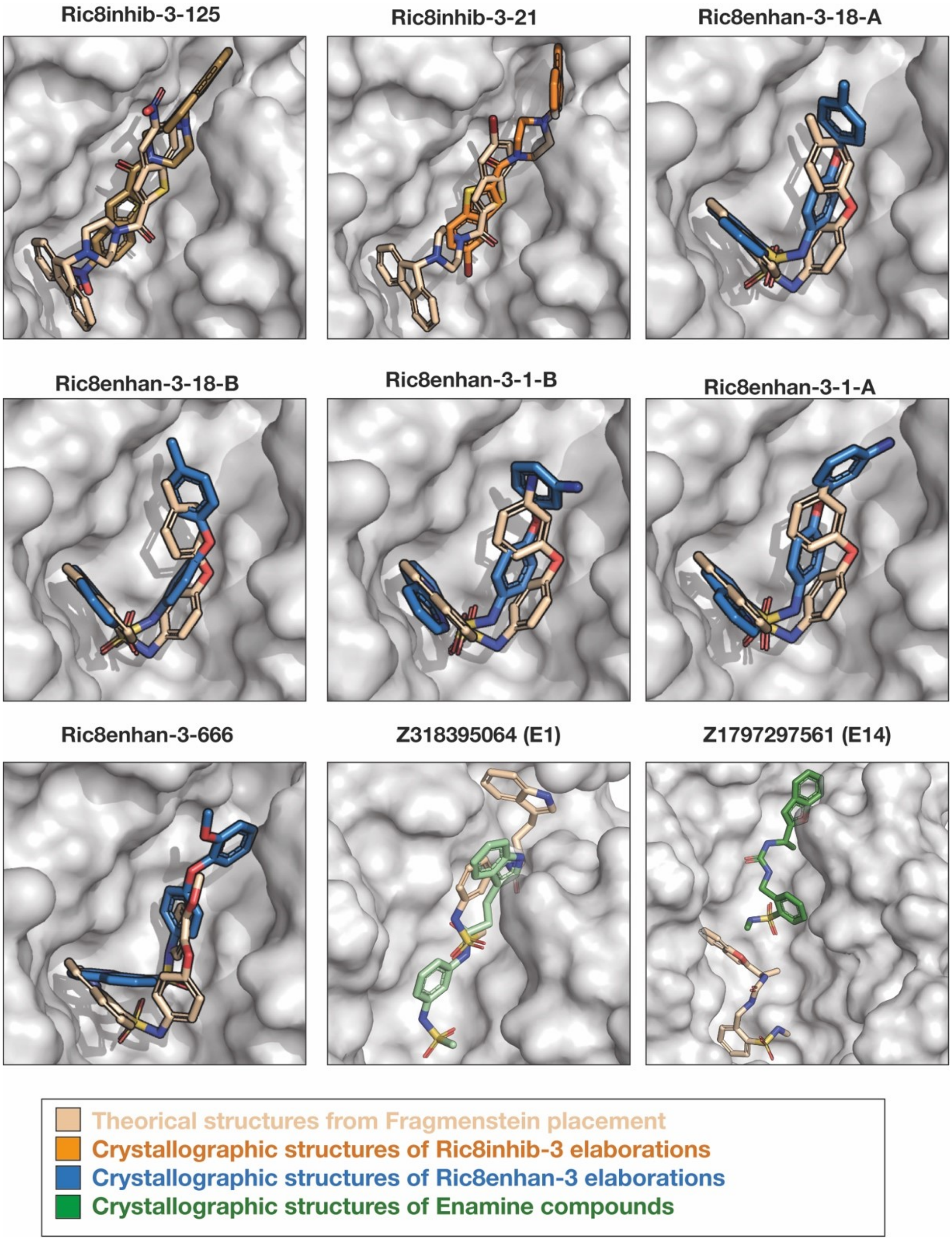

Figure S12. Superimposition of crystallographic structures of various elaborations relative to placements predicted by Fragmenstein. The placements proposed by Fragmenstein, based on fragment structures, are shown as light orange sticks. The crystallographic structures of Ric8inhib-3 elaborations are displayed as orange sticks, Ric8enhan-3 elaborations as blue sticks, and catalog compounds as green sticks.

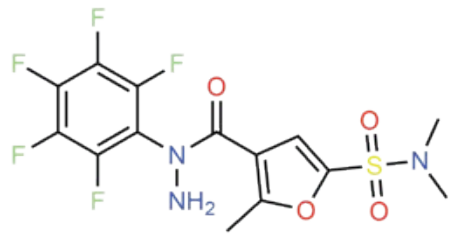

Ric8A-inhib-1-1111
NCS1-x1888
Ligand A – Chain A

Fragments: x0119-2B x0311-1A RMSD: 3.42 Å

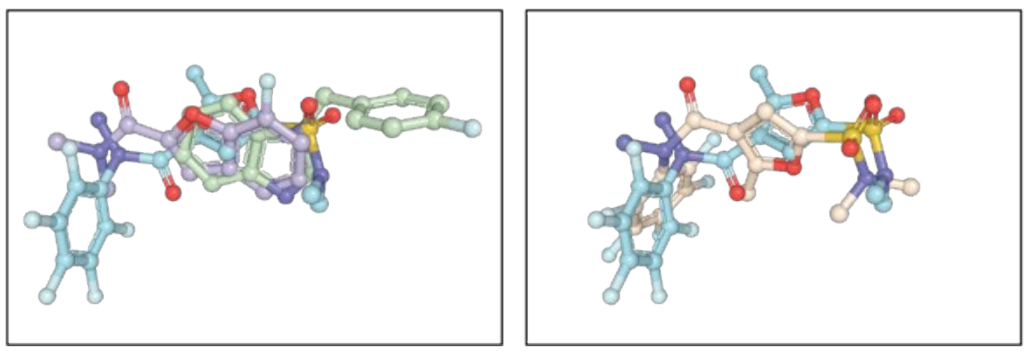

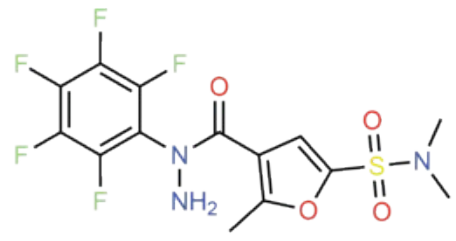

Ric8A-inhib-1-1111
NCS1-x1888
Ligand B – Chain A

Fragments: x0119-2B x0311-1A RMSD: 8.29 Å

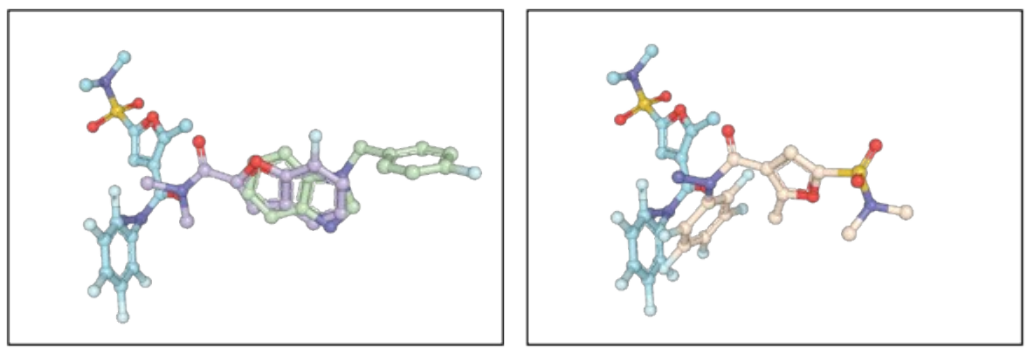

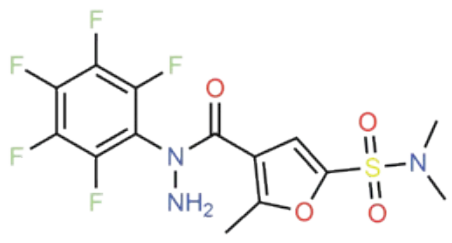

Ric8A-inhib-1-1111
NCS1-x1888
Ligand A – Chain B

Fragments: x0119-2B x0311-1A RMSD: 8.23 Å

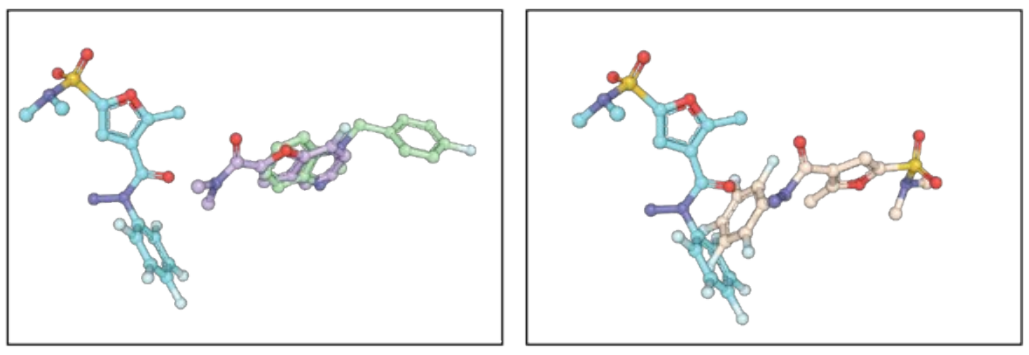

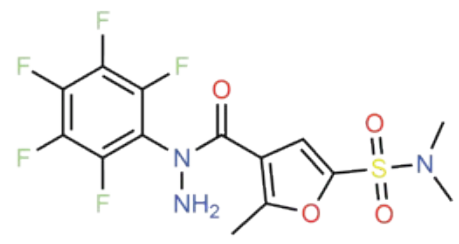

Ric8A-inhib-1-1111
NCS1-x1888
Ligand B – Chain B

Fragments: x0119-2B x0311-1A RMSD: 3.80 Å

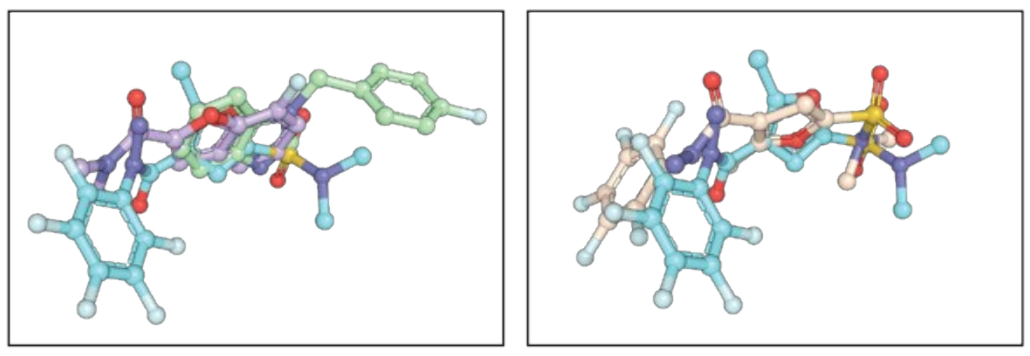

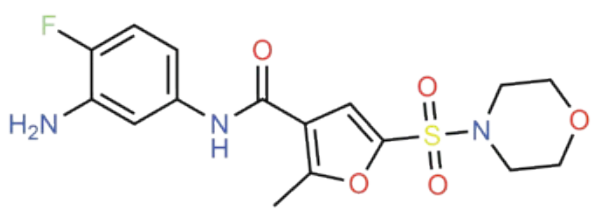

Ric8A-inhib-1-518
NCS1-x2005
Chain B

Fragments: x0119-2B x0311-1A RMSD: 8.38 Å

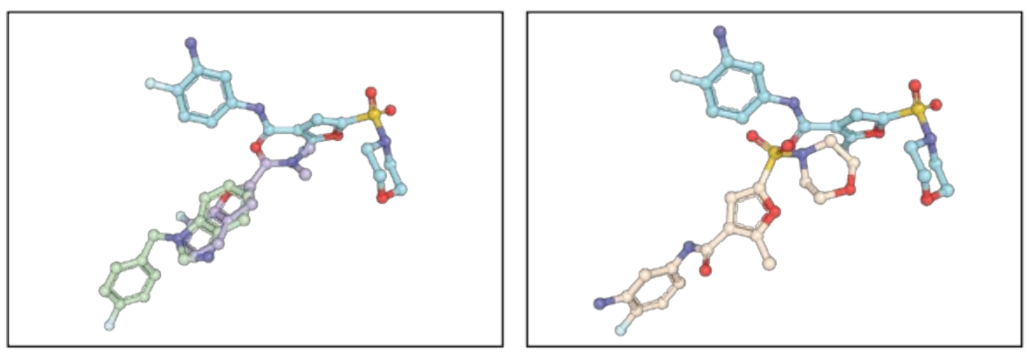

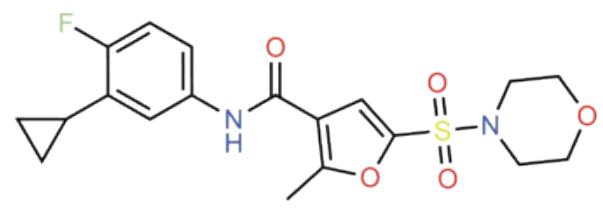

Ric8A-inhib-1-1463
NCS1-x1740
Chain B

Fragments: x0119-2B x0311-1A RMSD: 8.76 Å

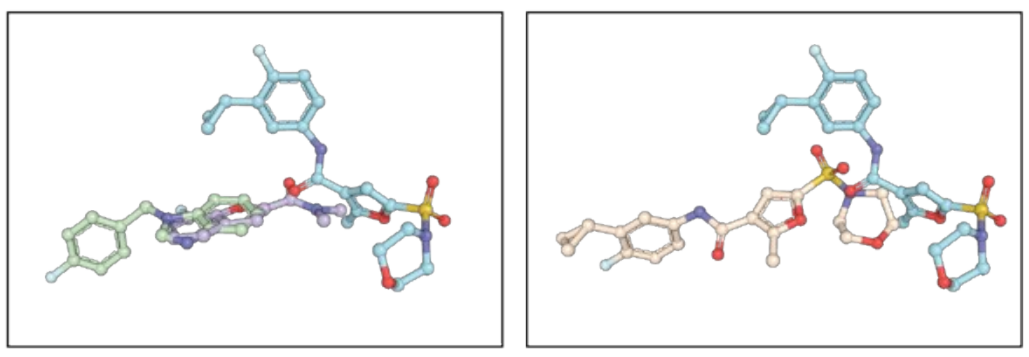

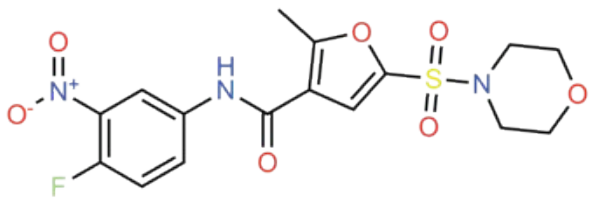

Ric8A-inhib-1-1287
NCS1-x1696
Chain A

Fragments: x0119-2B x0311-1A RMSD: 4.23 Å

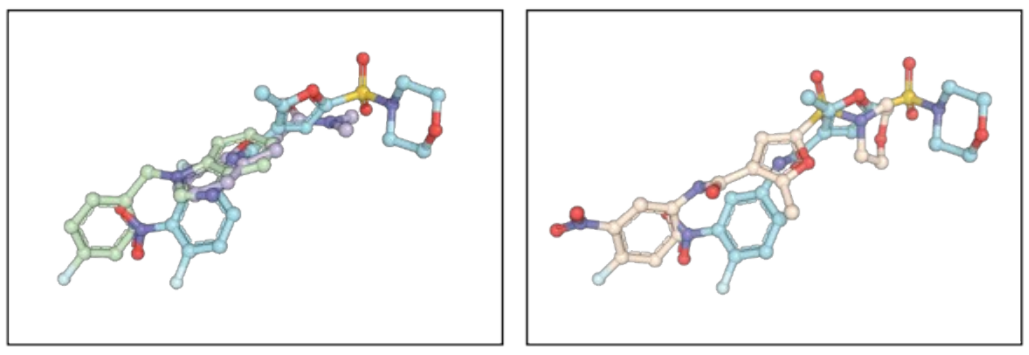

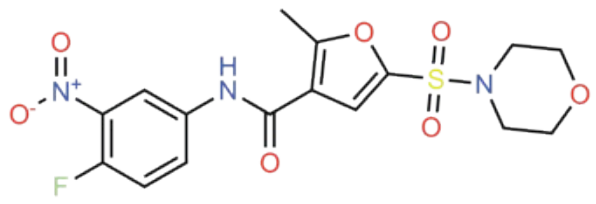

Ric8A-inhib-1-1287
NCS1-x1696
Chain B

Fragments: x0119-2B x0311-1A RMSD: 4.34 Å

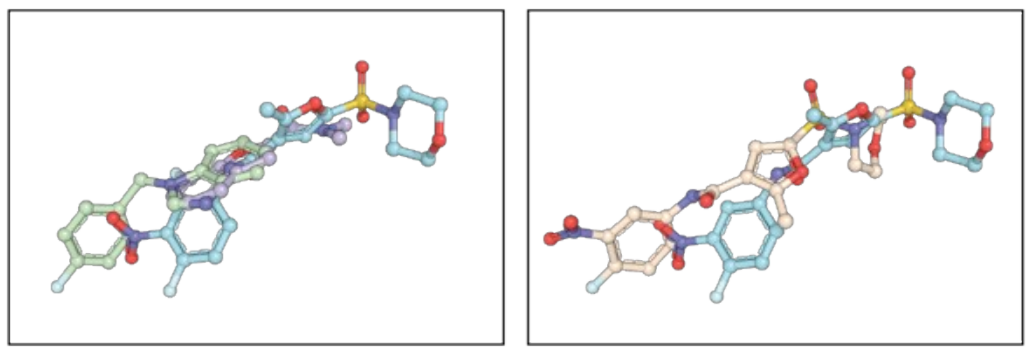

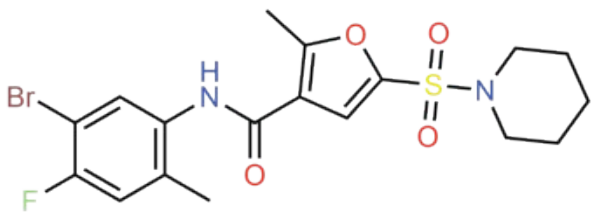

Ric8A-inhib-1-827
NCS1-x1943
Chain A

Fragments: x0119-2B x0311-1A RMSD: 4.60 Å

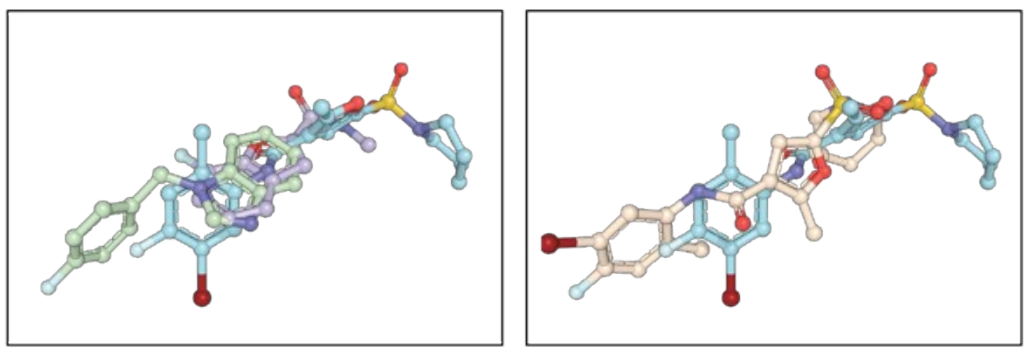

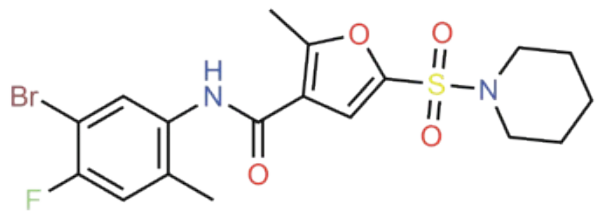

Ric8A-inhib-1-827
NCS1-x1943
Chain B

Fragments: x0119-2B x0311-1A RMSD: 4.47 Å

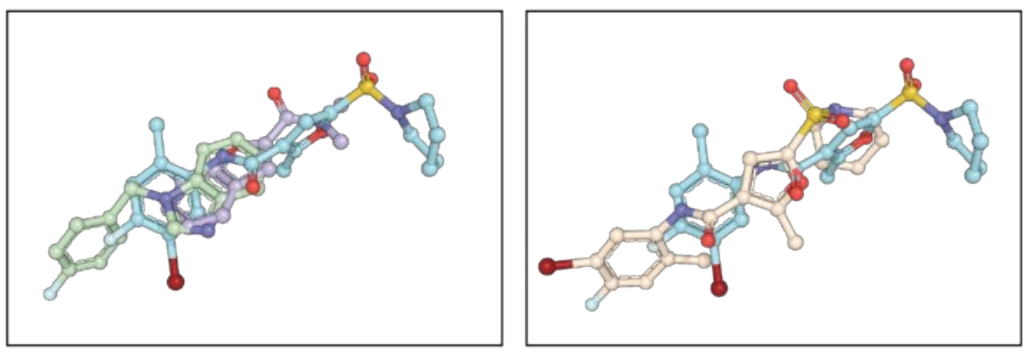

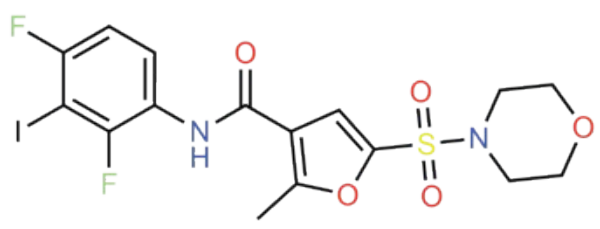

Ric8A-inhib-1-854
NCS1-x1726
Chain B

Fragments: x0119-2B x0311-1A RMSD: 4.47 Å

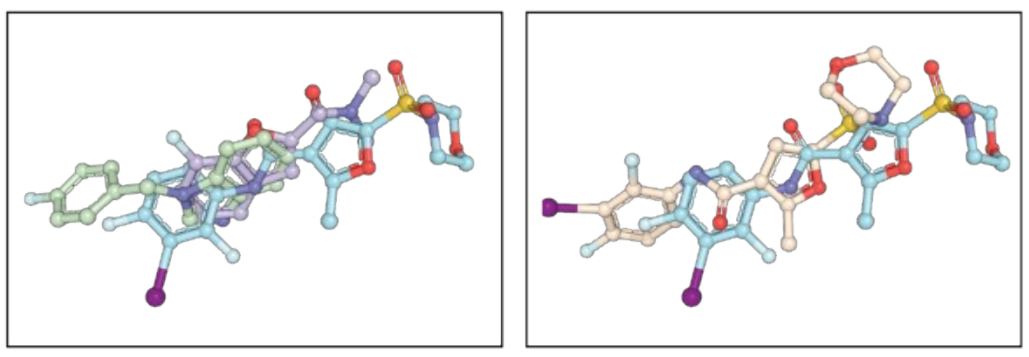

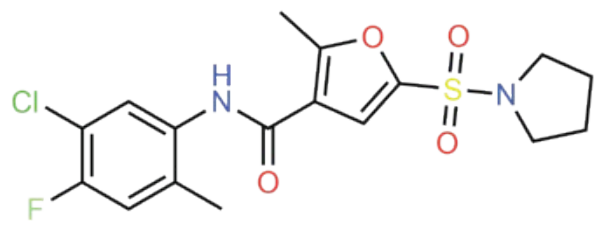

Ric8A-inhib-1-655
NCS1-x1894
Chain A

Fragments: x0119-2B x0311-1A RMSD: 5.19 Å

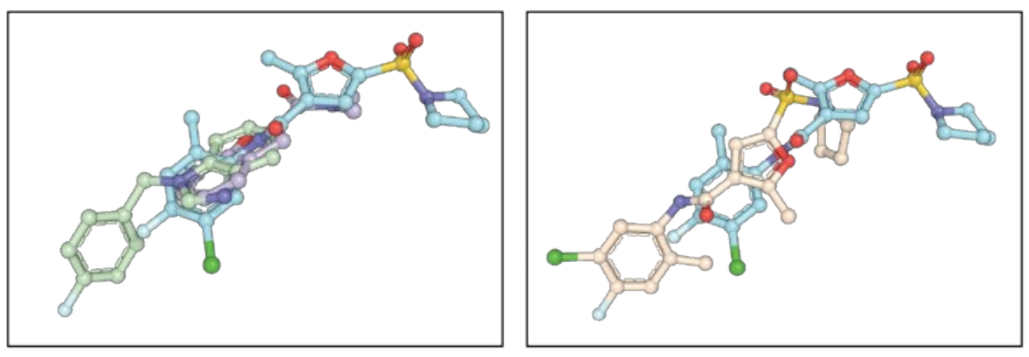

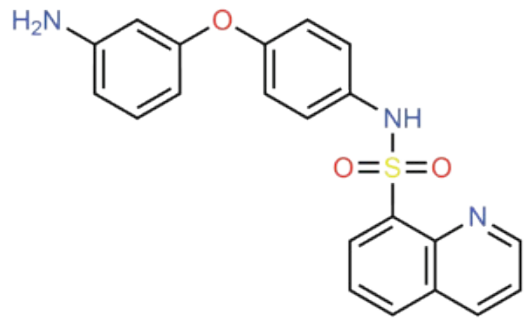

Ric8A-enhan-3-1
NCS1-x0975
Chain A

Fragments: x0119-2B, x0311-1A — RMSD: 2.76 Å

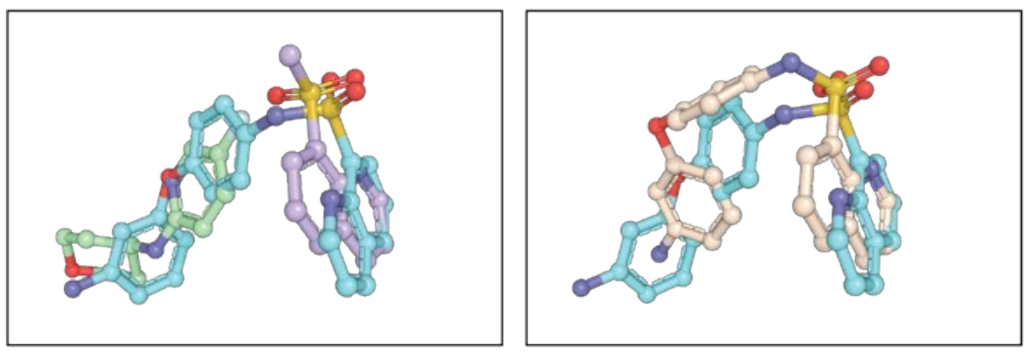

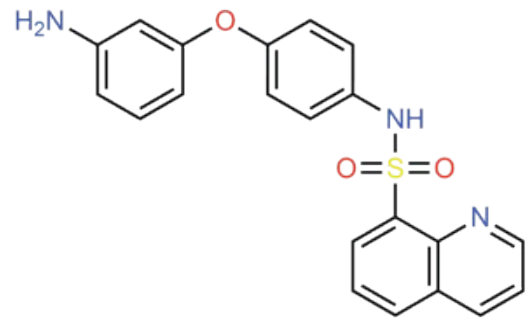

Ric8A-enhan-3-1
NCS1-x0975
Chain B

Fragments: x0119-2B, x0311-1A — RMSD: 2.93 Å

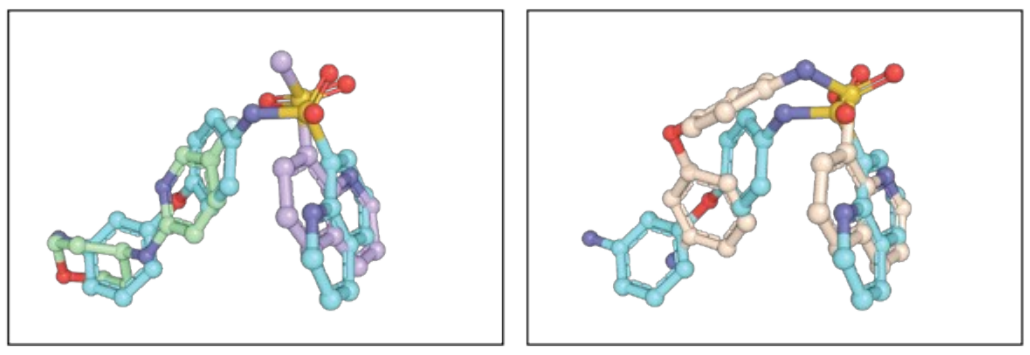

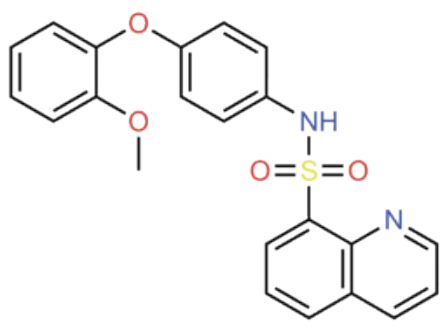

Ric8A-enhan-3-666
NCS1-x0981
Chain A

Fragments: x0071-0B, x0119-2B — RMSD: 3.26 Å

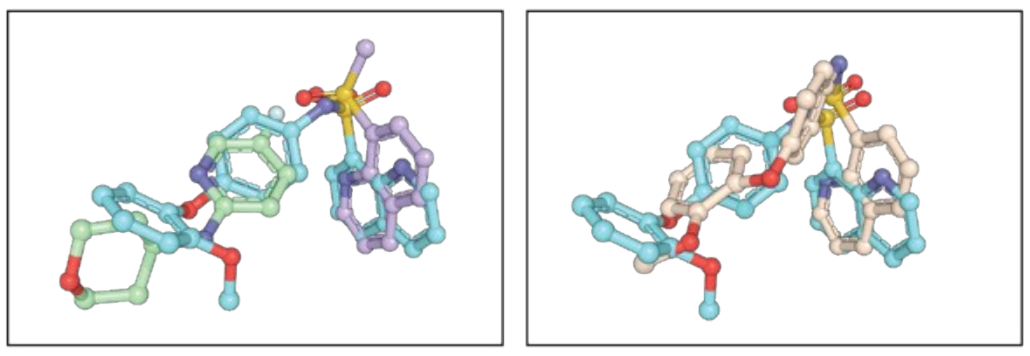

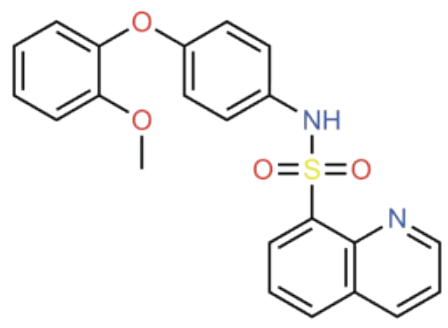

Ric8A-enhan-3-666
NCS1-x0981
Chain B

Fragments: x0071-0B, x0119-2B — RMSD: 4.22 Å

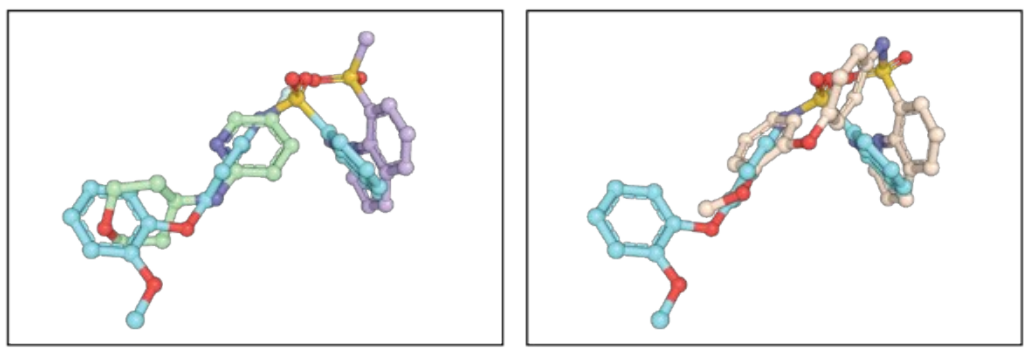

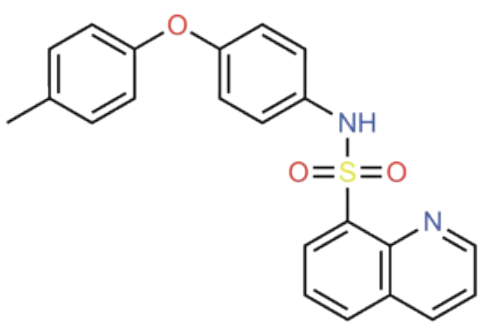

Ric8A-enhan-3-18
NCS1-x0974
Chain B

Fragments: x0125-0A, x0580-0A — RMSD: 2.52 Å

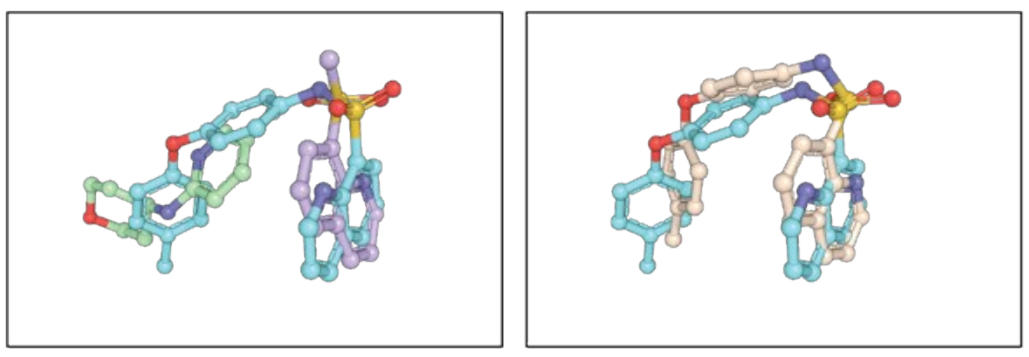

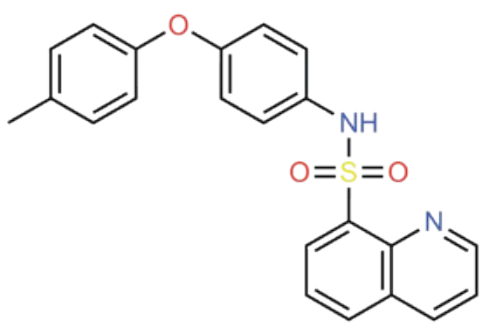

Ric8A-enhan-3-18
NCS1-x0974
Chain A

Fragments: x0125-0A, x0580-0A — RMSD: 2.90 Å

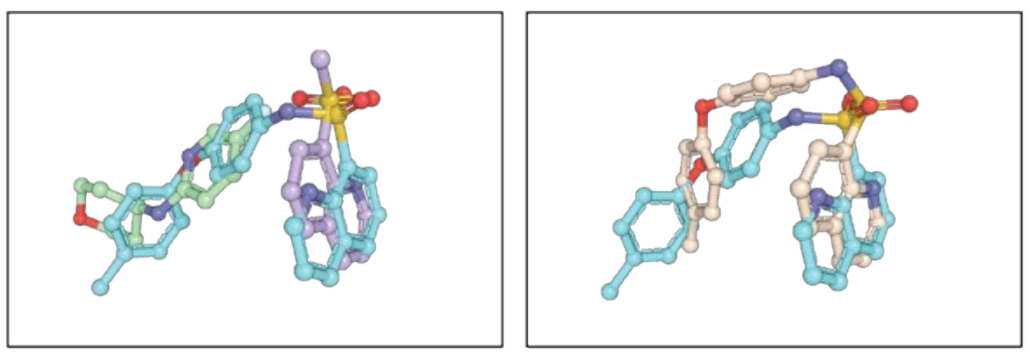

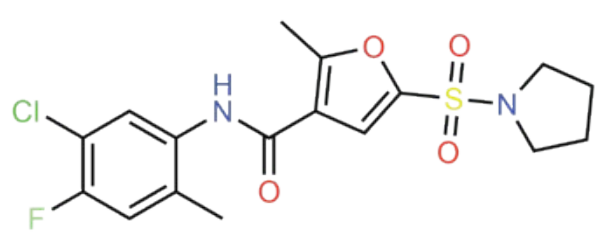

Ric8A-inhib-1-655
NCS1-x1894
Chain B

Fragments: x0119-2B x0311-1A RMSD: 4.97 Å

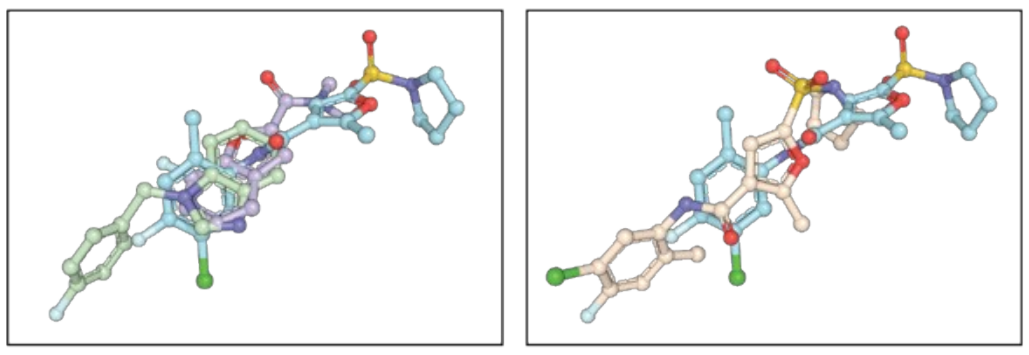

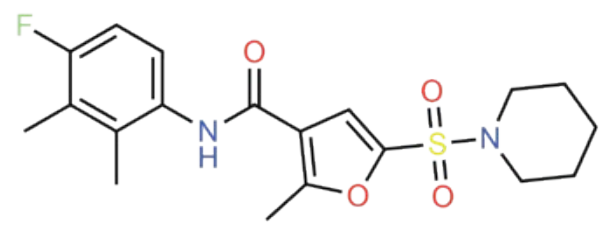

Ric8A-inhib-1-946
NCS1-x1942
Chain A

Fragments: x0119-2B x0311-1A RMSD: 3.82 Å

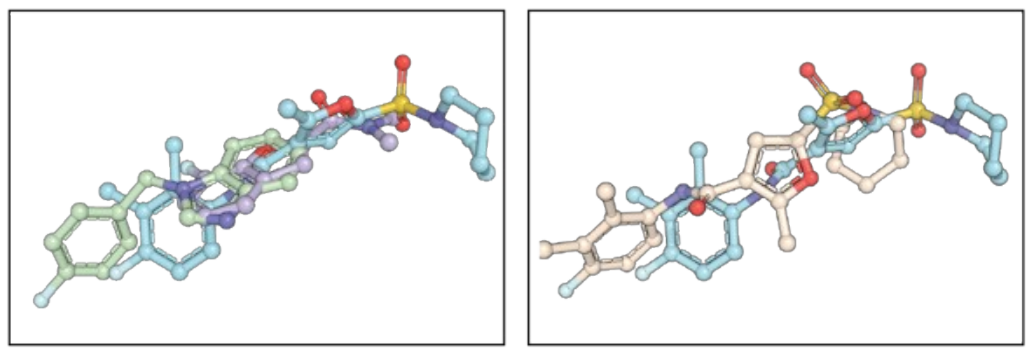

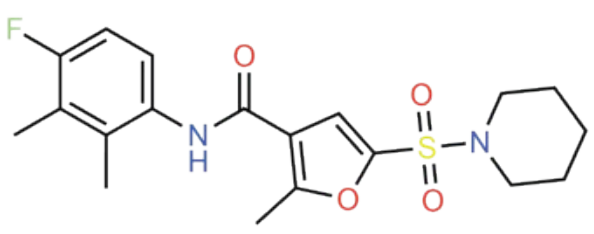

Ric8A-inhib-1-946
NCS1-x1942
Chain B

Fragments: x0119-2B x0311-1A RMSD: 4.16 Å

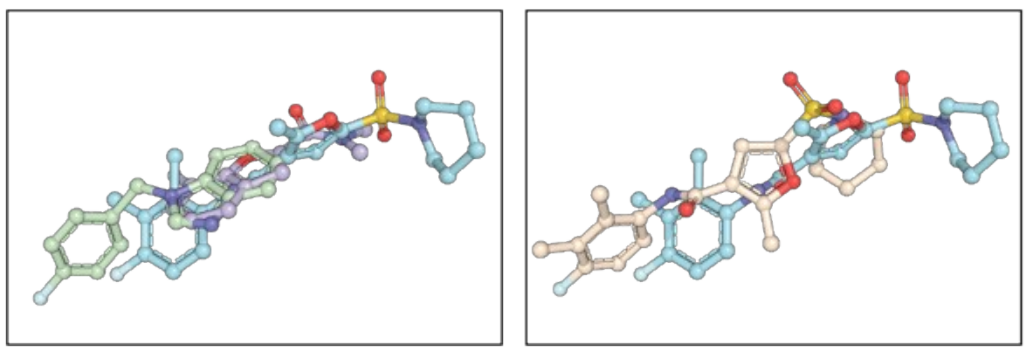

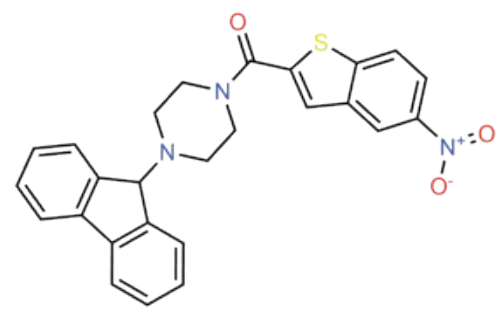

Ric8A-inhib-3-125
NCS1-x1698
Chain A

Fragments: x0071-0B x0119-2B RMSD: 10.94 Å

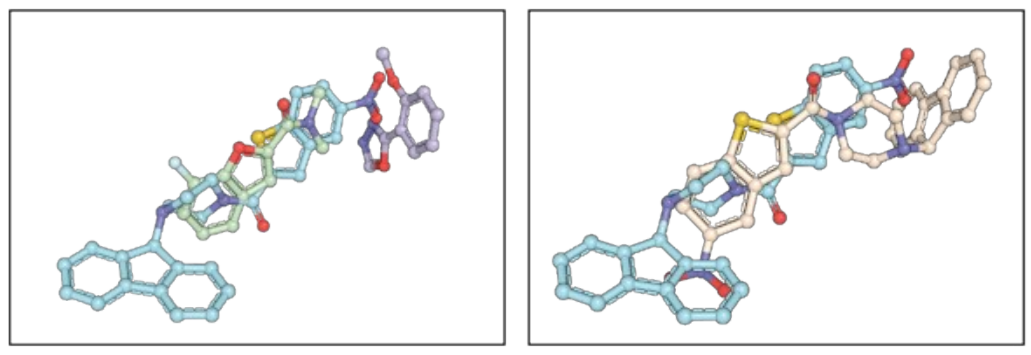

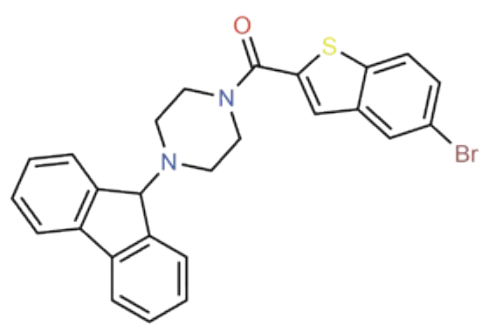

Ric8A-inhib-3-21
NCS1-x1699
Chain B

Fragments: x0119-2B x0071-0B RMSD: 12.16 Å

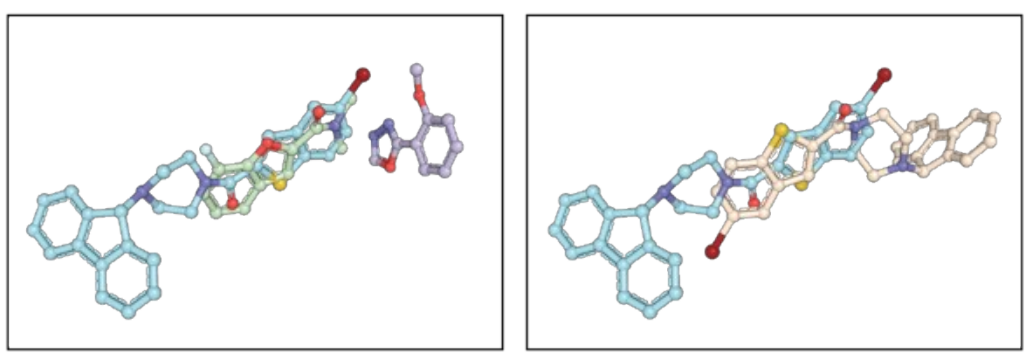

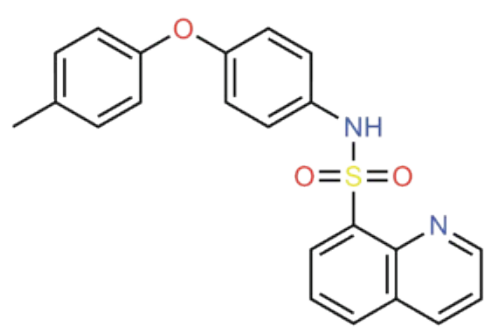

Ric8A-enhan-3-18
NCS1-x0974
Chain A

Fragments: x0125-0A x0580-0A RMSD: 2.90 Å

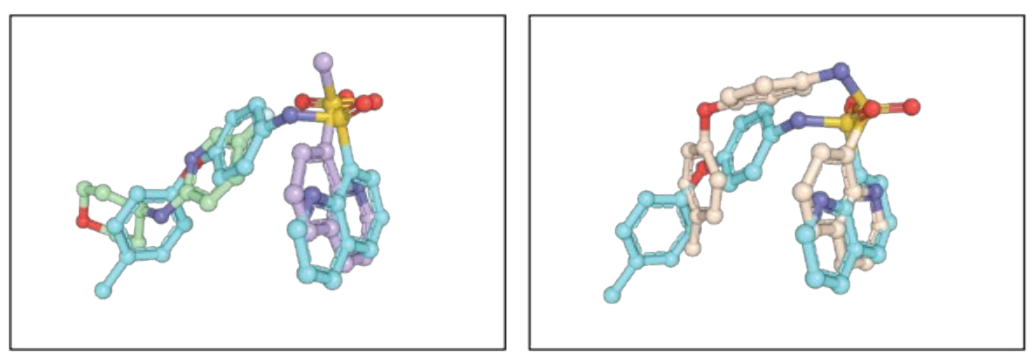

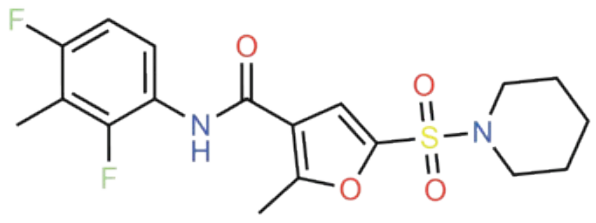

Ric8A-inhib-1-792
NCS1-x1238
Chain A

Fragments: x0119-2B x0311-1A RMSD: 5.49 Å

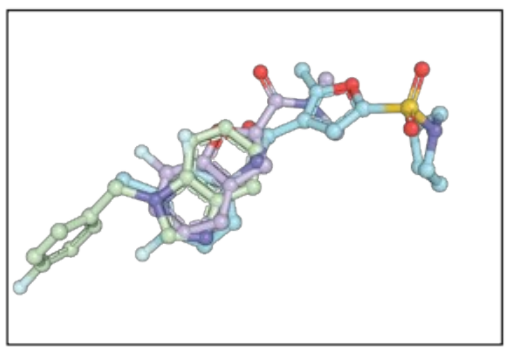

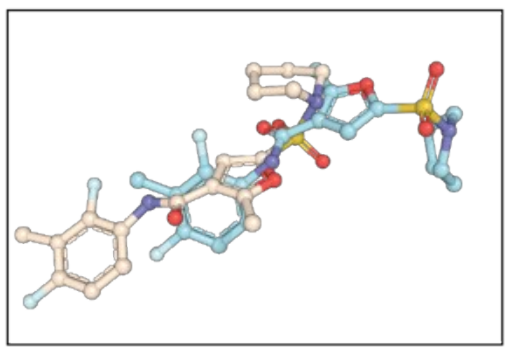

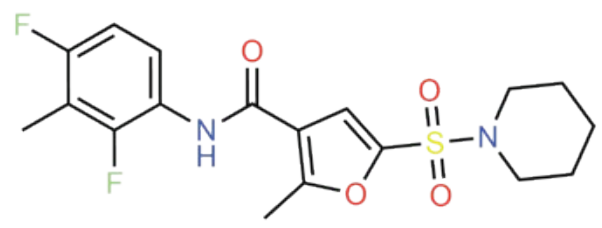

Ric8A-inhib-1-792
NCS1-x1238
Chain B

Fragments: x0119-2B x0311-1A RMSD: 4.85 Å

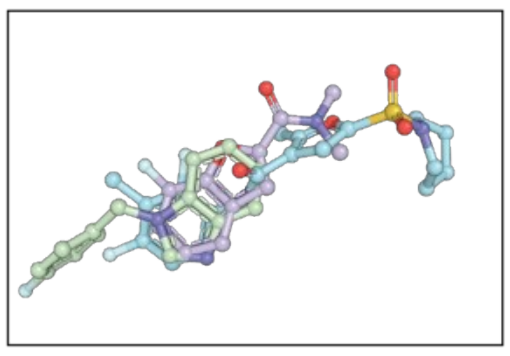

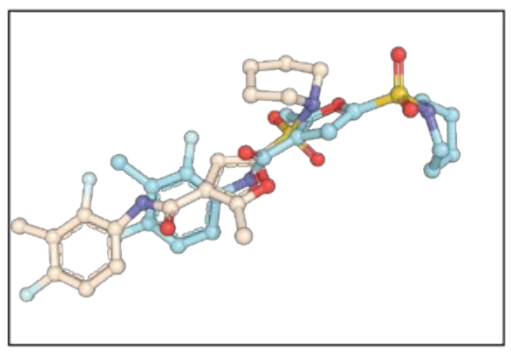

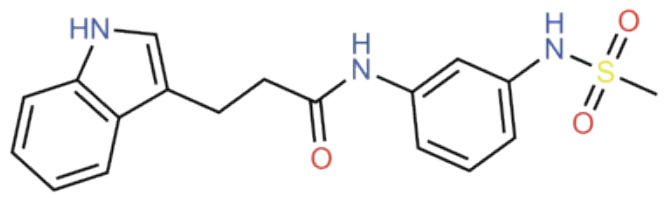

E1 (Z318395064)
NCS1-x1748
Chain A

Fragments: x0110-2B x0252-2B RMSD: 15.57 Å

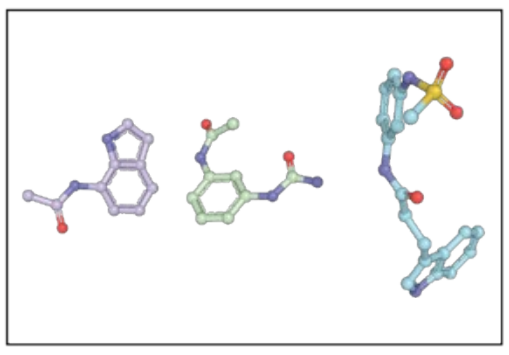

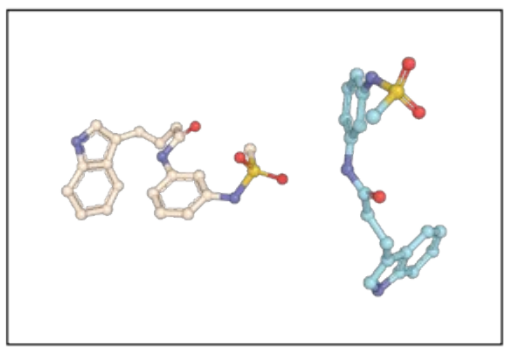

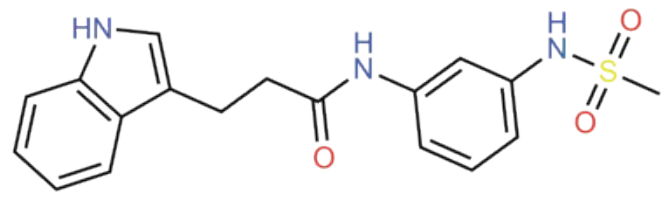

E1 (Z318395064)
NCS1-x1748
Chain B

Fragments: x0110-2B x0252-2B RMSD: 5.87 Å

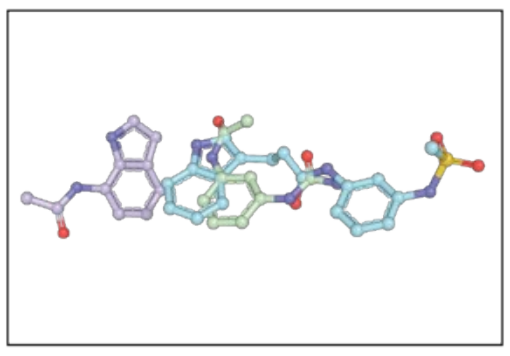

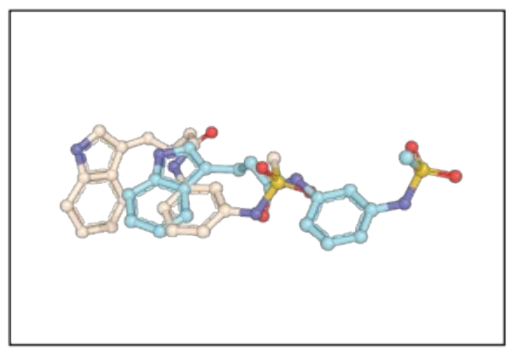

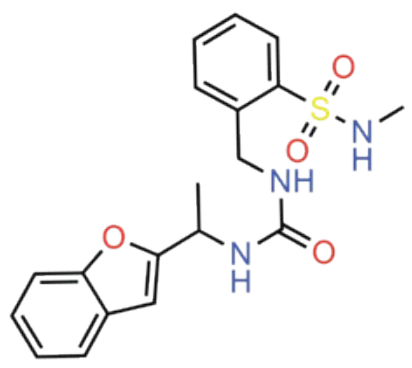

E14 (Z1797297561)
NCS1-x1777
Chain A

Fragments: x0119-0B x0469-0B RMSD: 12.20 Å

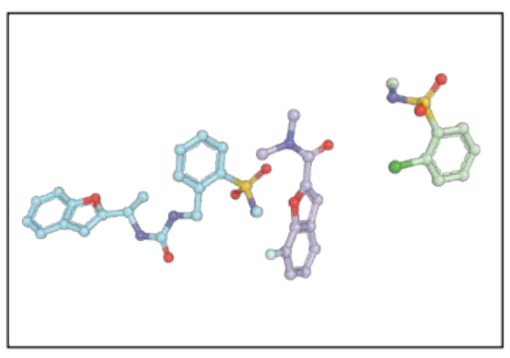

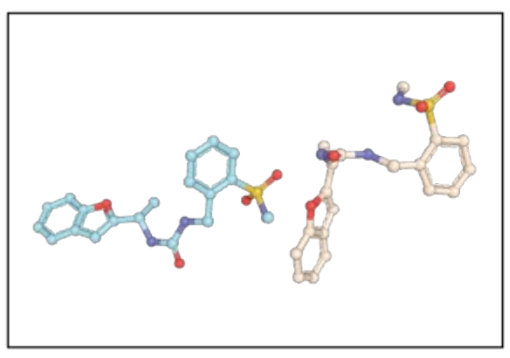

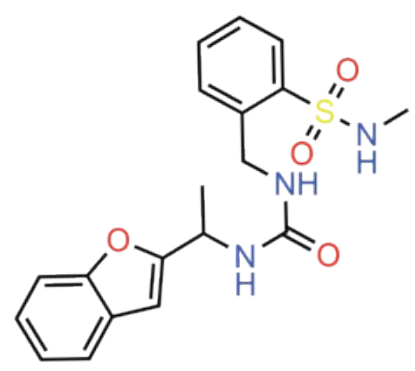

E14 (Z1797297561)
NCS1-x1777
Chain B

Fragments: x0119-0B x0469-0B RMSD: 12.55 Å

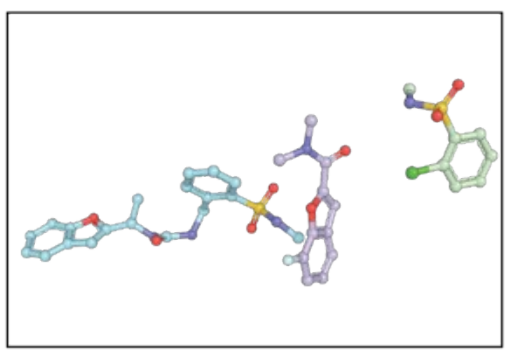

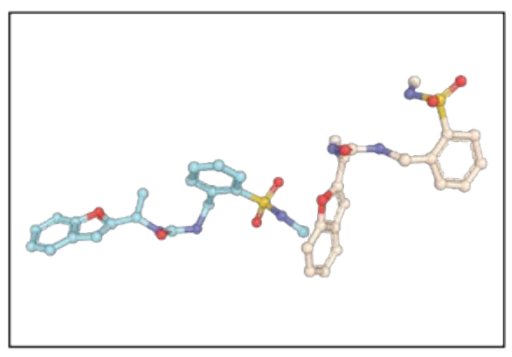

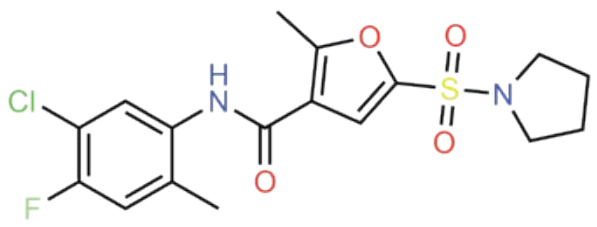

Ric8A-inhib-1-655
NCS1-x1894
Chain B

Fragments: x0119-2B x0311-1A RMSD: 4.97 Å

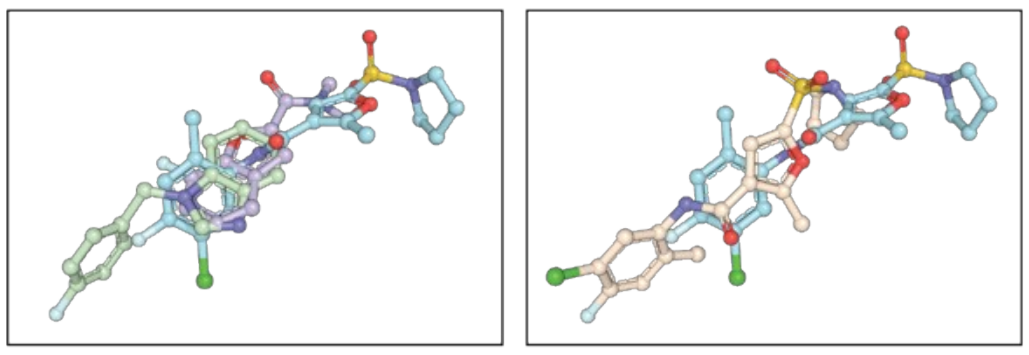

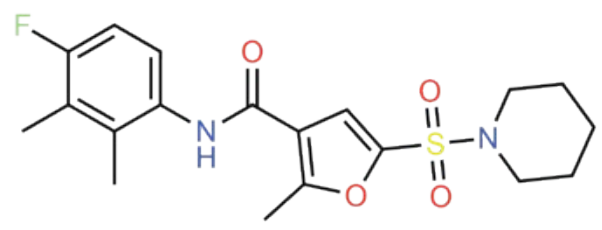

Ric8A-inhib-1-946
NCS1-x1942
Chain A

Fragments: x0119-2B x0311-1A RMSD: 3.82 Å

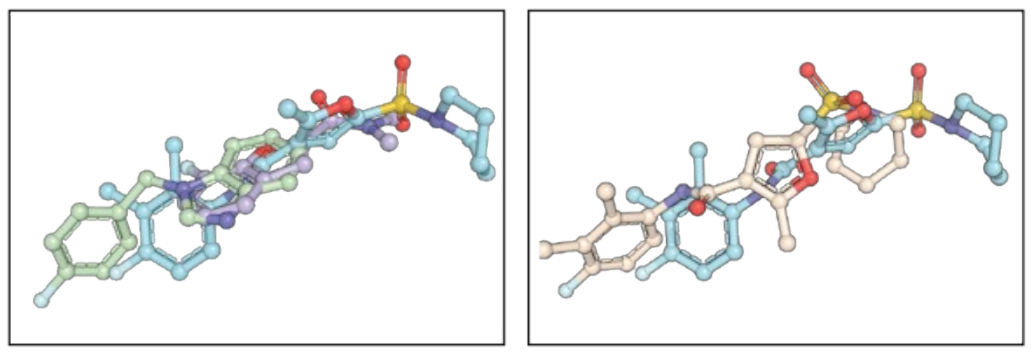

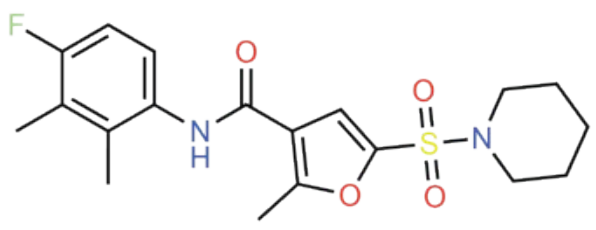

Ric8A-inhib-1-946
NCS1-x1942
Chain B

Fragments: x0119-2B x0311-1A RMSD: 4.16 Å

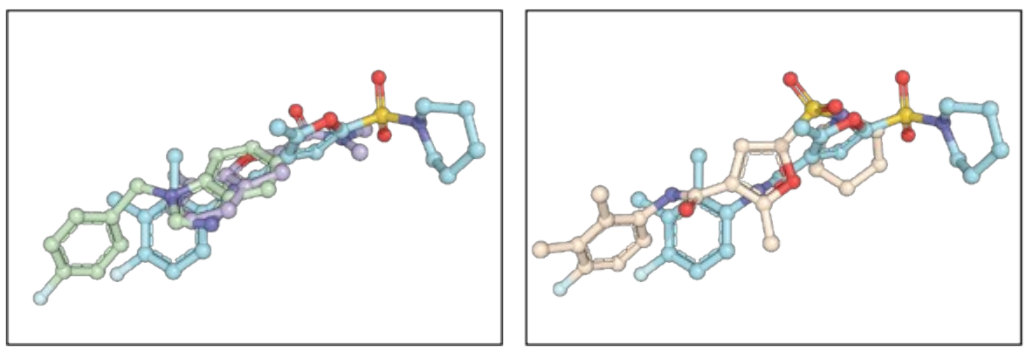

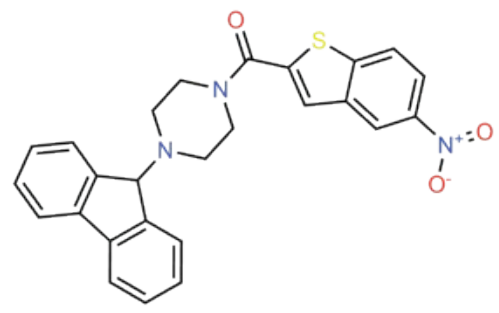

Ric8A-inhib-3-125
NCS1-x1698
Chain A

Fragments: x0071-0B x0119-2B RMSD: 10.94 Å

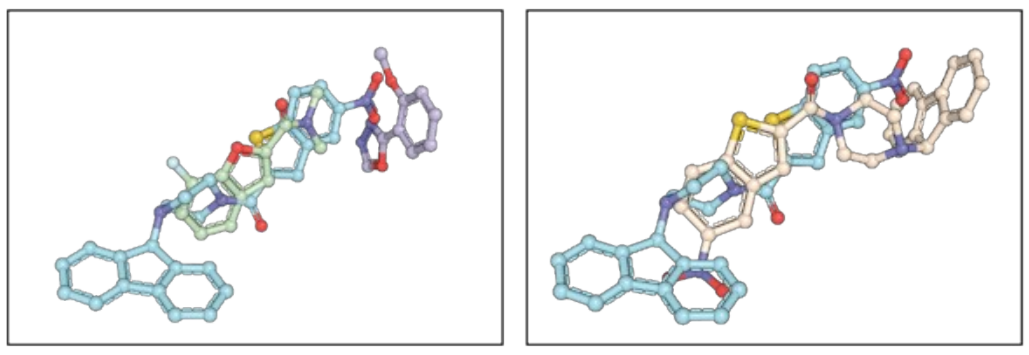

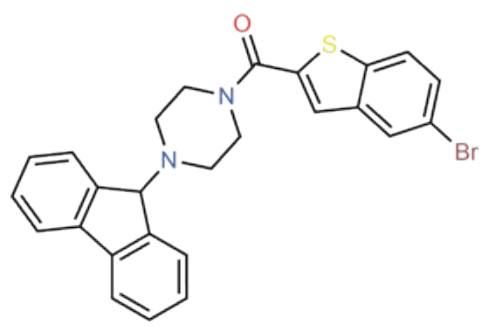

Ric8A-inhib-3-21
NCS1-x1699
Chain B

Fragments: x0119-2B x0071-0B RMSD: 12.16 Å

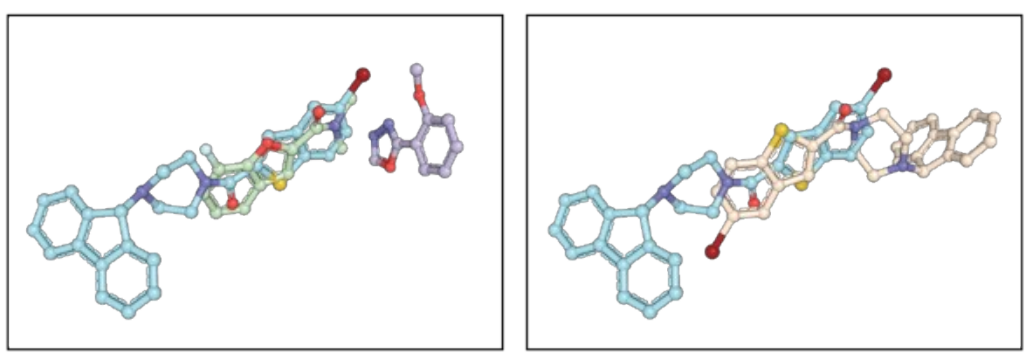

Figure S13. Superimposition of crystallographic structures of various elaborations relative to crystallographic structures of their inspiration fragments. For each compound resolved, the 2D structure, name, Fragalysis name, and chain are labeled. The inspiration fragments for the design are listed and the resolved structures of the fragments (purple and green) are overlaid with the resolved follow-up compound

(blue). The RMSD to the predicted pose (cream) is listed and is shown in relation to the resolved structure (blue).

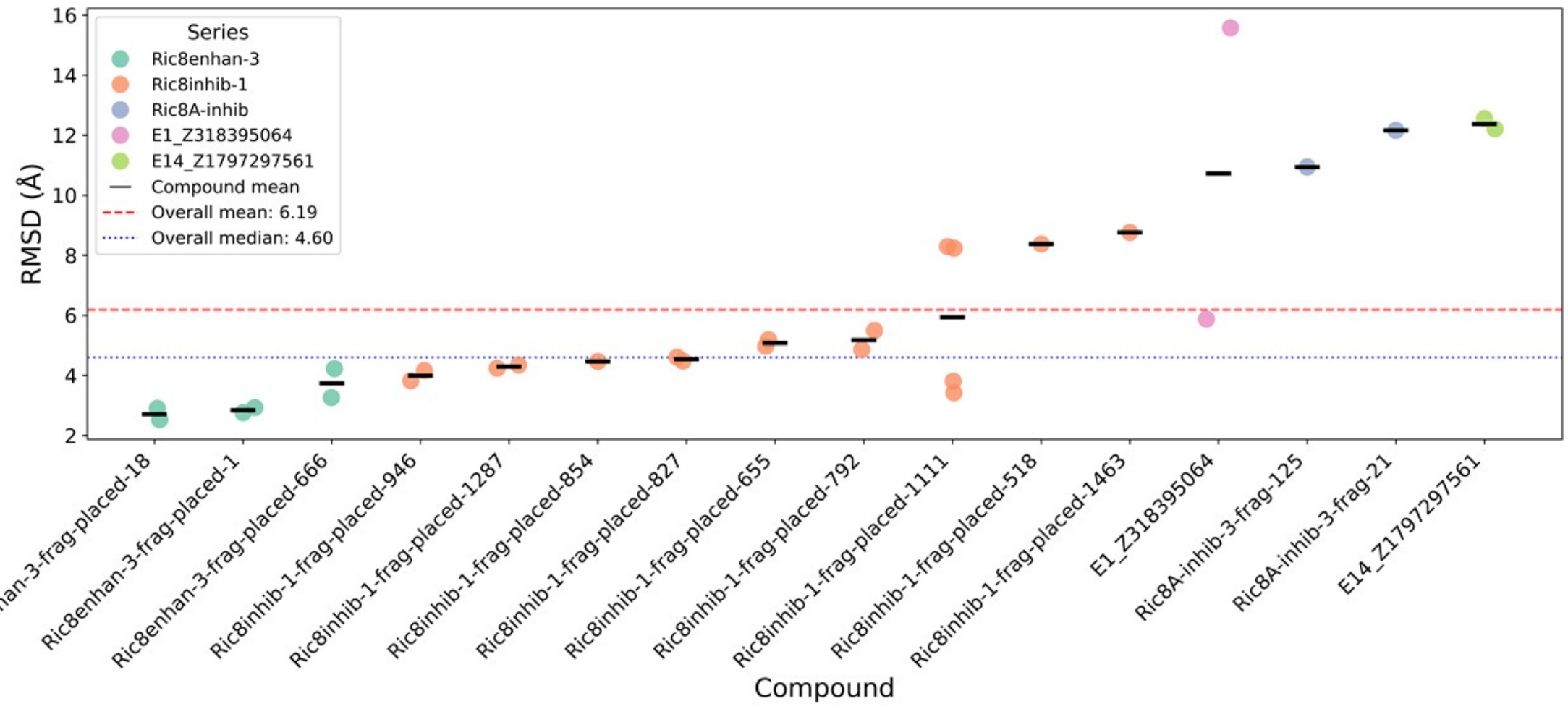

Figure S14. RMSD of experimentally resolved poses to the predicted pose by compound. Each point is one RMSD measurement; black horizontal bars show the compound mean. Compounds are sorted by mean RMSD and colored by series. Red dashed and blue dotted lines indicate the overall mean and median RMSD across all compounds, respectively.

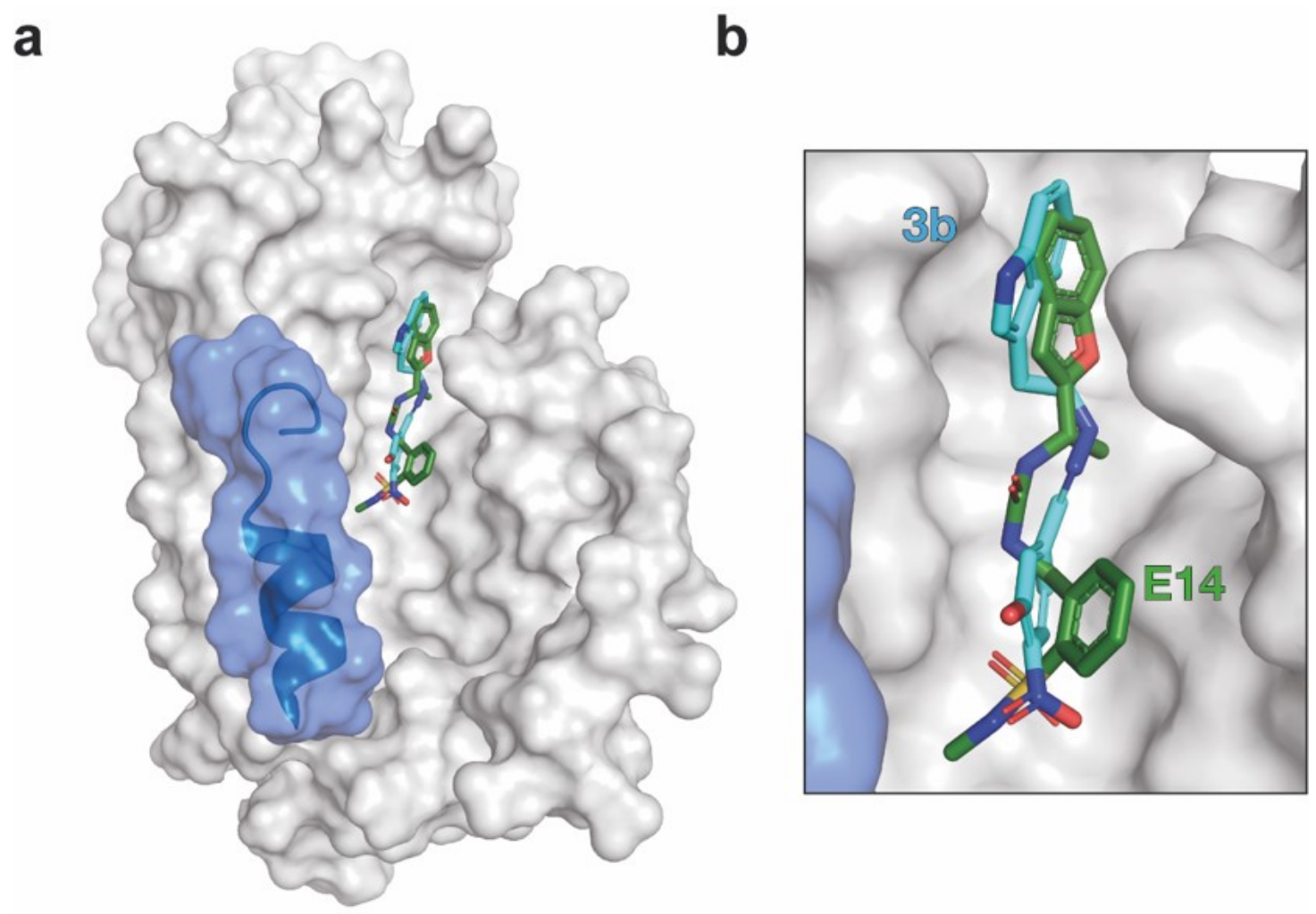

Figure S15. Superimposition of the crystallographic structures of the NCS-1/Ric-8A PPI enhancer 3b and the pure compound E14. (a) NCS-1 is shown as white molecular surface, the C-terminal H10 helix is shown in blue, and the compounds 3b (cyan, PDB: 6QI4, (12)) and E14 (green) are shown as sticks. **(b)** Magnified view of the ligand-binding region, showing the overlap between 3b and E14 within the NCS-1 crevice.

3. Supplementary tables

Table S1: Selected pure catalog compounds. (*) Indicates the Site (S) where the inspiration fragments were found in the fragment screening. (**) Hydrogen bonds, π-π interactions, and hydrophobic interactions were considered.

		Fragmenstein			Predicted key interactions (**)							
**Pure compounds**	**Fragments (*)**	**ΔG (kcal/mol)**	**RMSD (Å)**	**Tanimoto**	**W30 (**)**	**D37**	**Y52**	**T92**	**K100**	**W103 (**)**	**Y108**	**R148**
Z318395064 (E1)	Z1255459547 (S1) + Z198195770 (S2)	-10.1	0.78	0.36	X	X	X	X				
Z2005021751 (E2)	Z1255459547 (S1) + Z133716556 (S2)	-10.9	0.83	0.42			X	X		X		
Z451568306 (E3)	Z1203730757 (S4) + Z54226006 (S7)	-9.7	0.44	0.32								X
Z1097155203 (E4)	Z239127534 (S1) + Z26333448 (S2)	-8.0	0.50	0.45	X	X		X				
Z57379493 (E5)	Z55290386 (S3) + Z1269220427 (S4)	-12.0	0.88	0.48			X	X		X		
Z1260603230 (E6)	Z552903867 (S3) + Z126922042 (S4)	-12.0	0.88	0.48			X	X		X		
Z1104573517 (E7)	Z1255459547 (S1) + Z198195770 (S2)	-12.0	0.79	0.33	X	X	X					
Z1556583850 (E8)	Z300081412 (S5) + Z1272480091 (S6)	-9.5	0.68	0.38			X			X	X	
Z952525226 (E9)	Z29692148 (S1) + Z26333448(S2)	-7.2	0.64	0.58	X	X						
Z1396906847 (E10)	Z26333448 (S2) + Z1265813904 (S3)	-9.6	0.99	0.28			X	X				
Z1367764037 (E11)	POB0063 (S5) + Z1272480091 (S6)	-11.4	0.99	0.34				X				
Z1870241397 (E12)	Z166605460 (S4) + Z1272480091(S6)	-11.0	0.99	0.24			X		X		X	
Z2234189231 (E13)	Z31723394 (S2) + Z55290386 (S3)	-9.7	0.92	0.23	X	X	X	X		X		
Z2234189231 (E14)	Z1265813904 (S4) + Z45527714 (S7)	-12.8	0.99	0.38			X	X				
Z1798494323 (E15)	Z1255459547 (S4) + Z300081412 (S5)	-11.1	0.85	0.35			X					
Z3401857786 (E16)	Z1255459547 (S1) + Z26333448 (S2)	-7.5	0.96	0.62	X	X		X				
Z7823261935 (E17)	Z1198177230 (S4) + Z32327641 (S5)	-9.3	0.61	0.33						X		X

Table S2: Comparison of affinity results obtained via GCI and Trp fluorescence emission techniques.

Pure compound	K_d, (µM) GCI	K_d, (µM) Trp fluorescence emission
Z1260603230 (E6)	63.71	27.5 ± 5.1
Z1396906847 (E10)	97.31	398.5 ± 98.6
Z1870241397 (E12)	103.74	34.5 ± 15.0
Z2234189231 (E14)	102.82	77.62 ± 0.02
Z3401857786 (E16)	110.23	98.5 ± 5.6
Z7823261935 (E17)	141.45	263.4 ± 44.8

Table S3: Summary of the different steps during GCI for NCS-1 capture on the NTA surface.

Step	Solution	Concentration	Time (s)	Flux (µl/min)
Activation	$NiCl_2$	500 µM	60	30
Wash	EDTA	3 mM	60	30
Capture	His-NCS-1	15 µg/ml	60	10
Sample	Pure compounds	100 µM	25	100
Regeneration	EDTA	350 mM	60	30
